# Image-Based Prediction of Food Weight and Nutritional Composition in Bowl-Served Meals Using Semantic Segmentation and Multi-View 3D Reconstruction

**DOI:** 10.3390/nu18132119

**Published:** 2026-06-30

**Authors:** Xu Ji, Yiran Feng, Haolin Lu, Dongming Chu, Qiaosheng Han

**Affiliations:** 1 Department of Mechanical Engineering and Automation, Dalian Polytechnic University, Dalian 116034, China; 240420855000425@xy.dlpu.edu.cn (X.J.); 15542557209@163.com (H.L.); 250440855000457@xy.dlpu.edu.cn (D.C.); 2 SKL of Marine Food Processing & Safety Control, National Engineering Research Center of Seafood, Dalian Polytechnic University, Dalian 116034, China; 3 Department of Key Laboratory of Marine Food Processing Technology and Equipment of Liaoning Province, Dalian Polytechnic University, Dalian 116034, China; 4 College of Arts & Information Engineering, Dalian Polytechnic University, Dalian 116499, China

**Keywords:** semantic segmentation, 3D reconstruction, height-field integration, nutritional composition prediction

## Abstract

**Background:** Image-based dietary assessment provides a more intuitive approach for nutritional monitoring and health management. However, in multi-category bowl-based meals, food boundary adhesion, spatial stacking, and staple-food occlusion by upper-layer dishes still affect the accuracy of volume, weight, and nutritional composition prediction. **Methods:** This study proposes a nutrition prediction method for bowl-based foods by integrating semantic segmentation, multi-view three-dimensional reconstruction, and occlusion compensation. The improved DBP-FDSNet was used to extract food-category masks from top-view RGB images, while detail enhancement, boundary-assisted supervision, and spatial position encoding were incorporated to improve the segmentation quality of food boundaries and adhesion regions. The visible food surface inside the bowl was reconstructed using a bowl instance model and RGB-TSDF-based multi-view fusion, and the two-dimensional semantic results were mapped into the height-field parameter domain for category-level volume integration. For partially occluded, severely occluded, or completely invisible staple foods, a layered compensation strategy was introduced to reduce staple-food volume prediction errors and the erroneous assignment of upper-layer food volume. Food weight and whole-bowl Calories, Fat, Carbohydrate, and Protein were finally predicted using food density and a nutritional composition database. **Results:** DBP-FDSNet achieved a *mean* *Intersection* *over* *Union* (mIoU) of 80.51% and a *Boundary* *F*1 *Score* (bF1) of 85.73%. At the whole-bowl level, the *Mean* *Absolute* *Percentage* *Error* (MAPE) values for Calories, Fat, Carbohydrate, Protein, and total food mass were 13.23%, 18.51%, 14.18%, 13.35%, and 10.85%, respectively. **Conclusions:** The method improves the stability of category-level volume and nutritional composition prediction in complex bowl-based meal scenarios, providing a feasible solution for image-based dietary assessment and intelligent nutrition management.

## 1. Introduction

With the development of image recognition and deep learning, image-based dietary assessment has gradually become an important research direction in nutritional monitoring and health management. Compared with traditional methods such as manual weighing, dietary records, and questionnaire surveys, image-based methods can reduce reliance on users’ subjective memory and provide a more intuitive technical approach for food category recognition, intake estimation, and nutritional composition prediction [1]. However, existing image-based dietary assessment methods still face several challenges when applied to bowl-based meal scenarios [2,3]. For example, the geometry of bowls imposes clear constraints, while foods are usually stacked inside the bowl, and boundary adhesion as well as mutual occlusion often occur among different ingredients. Therefore, developing a nutritional composition prediction method for bowl-based meals that can jointly use semantic information, three-dimensional structural information, and container geometric constraints has important research value and practical significance.

**Figure 1:**
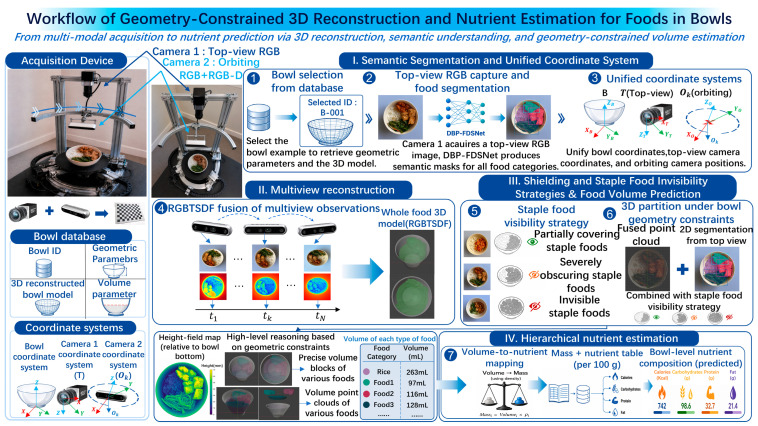
Overall flowchart for predicting the nutritional components of food in bowls.

The method consists of semantic segmentation, three-dimensional volume estimation, and nutritional composition mapping. Considering the instability of volume prediction in existing methods under conditions such as food boundary adhesion, vertical occlusion between upper and lower layers, and the invisibility of staple foods, a staged prediction framework was constructed. In this framework, DBP-FDSNet is first used as the basis for food category recognition, after which the bowl instance model and RGB-TSDF-based multi-view reconstruction are combined to recover the three-dimensional structure of the food. Category-level volume prediction is then performed through height-field volume integration, while a layered compensation strategy for staple foods is introduced to improve estimation stability under occlusion. The overall workflow of this method is shown in Figure 1.

The main contribution of this study lies in the construction of an image-based nutritional composition prediction pipeline for multi-category bowl-based foods. First, a food semantic segmentation model based on DBP-FDSNet was proposed, in which a detail-enhanced shallow branch, boundary-assisted supervision, and spatial position encoding were introduced into the original model to improve its ability to recognize food boundaries and adhesion regions. The experimental results show that the mIoU, bF1, and mean pixel classification accuracy (mAcc)of DBP-FDSNet reached 80.51%, 85.73%, and 90.06%, respectively, indicating that more accurate semantic segmentation masks can be provided for subsequent three-dimensional volume prediction. In addition, geometric constraints were provided in a unified bowl coordinate system through the bowl instance model, while the visible food surface inside the bowl was reconstructed using RGB-TSDF-based multi-view fusion. After height-field mapping and local correction, the two-dimensional semantic results could be stably mapped onto the three-dimensional food surface, with back-projection mIoU, *mean Dice coefficient* (mDice), and bF1 values of 86.9%, 92.8%, and 91.9%, respectively. Meanwhile, a layered volume estimation strategy based on the visible proportion of staple foods was proposed, and underestimation of staple-food volume as well as erroneous volume assignment to upper-layer foods was reduced through bowl-model constraints, staple-layer height completion, and supporting lower-boundary correction. For samples containing staple foods, the MAE and MAPE of staple-food weight prediction were 20.7 g and 9.64%, respectively. Finally, a prediction process from category-level volume prediction to mass conversion and then to whole-bowl nutritional composition prediction was established, in which food density and a nutritional composition database were used to calculate the contents of Calories, Fat, Carbohydrate, and Protein. The experimental results show that the MAPE values for whole-bowl Calories, Fat, Carbohydrate, and Protein prediction were 13.23%, 18.51%, 14.18%, and 13.35%, respectively.

Therefore, the contribution of this study should be understood as a scenario-oriented extension and structured improvement of the existing image-based dietary assessment pipeline. On the one hand, a comprehensive method is developed by combining bowl instance-based geometric constraints with multi-view RGB-TSDF reconstruction and category-level height fields, so that volume prediction no longer relies only on visible surfaces or global information [4]. In addition, considering that staple-food occlusion is particularly common in bowl-based meal scenarios, a layered compensation strategy is introduced to reduce the possibility that staple-food volume is underestimated or incorrectly assigned to upper-layer foods. Overall, the proposed method not only presents its improvements over existing methods more clearly, but also better reflects practical dining scenarios.

## 2. Research Status and Related Work of This Study

### 2.1. Current Status of Related Research

In recent years, related studies have mainly developed along two relatively distinct directions. The first line of research focuses on volume prediction based on container geometric modeling and visible-surface inference. In such methods, the tableware or bowl itself is usually used as a scale reference, while information such as the bowl rim contour, the overall food shape, and the visible food area is used to convert observable features in two-dimensional images into estimated food volume information. Yang et al. proposed a smartphone-based markerless method for food portion estimation, in which the motion sensors embedded in the phone and the imaging geometry in real scenes were used to recover scale information for both the tableware and the food [5]. Jia et al. further extended this research direction to circular bowl-shaped food scenarios and predicted the weight of food in a bowl using a single image [6]. In their subsequent study, a mathematical model of the bowl-shaped structure was constructed, and the relationship between the *Food area ratio (FAR)* and the filling amount inside the bowl was established, allowing the food volume in the bowl to be accurately predicted [7]. These methods are relatively interpretable and depend less on multi-source data, but when food is occluded by the bowl wall or covered by upper-layer ingredients, their volume prediction accuracy often decreases.

The second line of research relies on deep learning and related models for nutritional composition prediction, including multimodal fusion and end-to-end nutrition regression, in which nutritional components are predicted directly from images through techniques such as image segmentation, depth prediction, and vision-language contrastive learning [8]. Han et al. proposed the DPF-Nutrition model, in which an estimated depth map was generated by a depth prediction module and then combined with an RGB-D fusion module for nutritional composition prediction [9]. Zhao et al. adopted a two-stage framework based on segmentation and regression, where a U-Net was used to extract food regions before a regression network was applied to predict the contents of multiple nutritional components [10]. Feng et al. further introduced RGB-D information and combined it with a FLAVA-based contrastive learning model, achieving a lower *Percentage Mean Absolute Error (*PMAE*)* on the Nutrition5k dataset [11]. Meanwhile, Lo et al. proposed a deep learning-based single-depth-view synthesis method, in which food point clouds were reconstructed by predicting depth maps from different viewpoints, providing a new idea for three-dimensional volume estimation under occlusion conditions [12].

Different from the work of Han et al. [9], in which overall nutritional prediction was mainly performed through depth estimation and RGB-D fusion, this study further introduces a bowl instance model as a container-level geometric constraint for the subsequent prediction process. Meanwhile, the two-dimensional semantic results are mapped into the height-field parameter domain to support category-level volume integration. Compared with the method proposed by Lo et al. [12], where partial three-dimensional structures are recovered through single-depth-view synthesis, this study focuses on the more common problems of vertical occlusion and staple-food invisibility in multi-category bowl-based meals, and different compensation procedures are designed for three scenarios with different degrees of staple-food occlusion. Therefore, the main innovation of this study lies in the modeling of complex occlusion scenarios in bowl-based meals, rather than merely in the use of segmentation or three-dimensional reconstruction models.

### 2.2. Related Work of This Study

#### 2.2.1. Overall Workflow

The overall workflow is divided into several stages, including data acquisition, semantic segmentation, three-dimensional reconstruction, category-level volume estimation, and nutritional composition mapping and prediction [13]. First, the top-mounted RGB camera captures top-view images of bowl-served food, while the surrounding RGB-D camera on the slide rail continuously acquires RGB-D and RGB images from different viewpoints and records the corresponding camera poses during image acquisition. The specific model information of the equipment used in the experiments, together with the corresponding software details, will be provided in Section 4. DBP-FDSNet is applied to the top-view images to generate two-dimensional masks for different food categories, and the foods are further divided into three groups, namely staple foods, single-ingredient foods, and multi-ingredient foods. By using the bowl instance model and establishing a unified bowl coordinate system, the visible three-dimensional food surface inside the bowl is reconstructed through RGB-TSDF-based multi-view fusion. The two-dimensional semantic labels are then mapped into the height-field parameter domain, while staple-food layer compensation and supporting lower-boundary correction are used to handle cases in which staple foods are partially occluded, severely occluded, or completely invisible. The volume of each food category is predicted through category-level height-field integration. Finally, the estimated volume is converted into mass according to food density, and the whole-bowl Calories, Carbohydrate, Protein, and Fat contents are predicted using a nutritional composition database.

#### 2.2.2. Semantic Segmentation of Food Images

In this study, DBP-FDSNet was developed on the basis of the original FDSNet [14]. In this model, a detail-enhanced shallow branch was added to combine the original image with high-frequency residuals and gradient-based edge information, allowing food contours and local texture variations to be captured more effectively. At the same time, a boundary-assisted supervision branch was newly introduced to reduce boundary ambiguity and adhesion between adjacent food items. In addition, spatial position encoding was incorporated so that the model could better use the relatively stable spatial distribution patterns in bowl-based food scenes. In the experiments, DBP-FDSNet achieved an mIoU of 80.51%, a bF1 of 85.73%, and an mAcc of 90.06%. After pretraining on the UFP (UEC Food Pix Complete) dataset [15], the mIoU was further improved to nearly 83%, indicating that more accurate semantic masks can be provided for three-dimensional food volume prediction.

#### 2.2.3. The Concealment Strategies for Staple Foods and the 3D Reconstruction of Food Items

First, a unified bowl coordinate system is established by integrating the top-mounted RGB camera with the surrounding RGB-D cameras, and the reconstruction range is constrained using the bowl instance model. An RGB-TSDF-based multi-view fusion method [16] is adopted to reconstruct the visible three-dimensional food surface inside the bowl, and the reconstructed surface is further converted into a height-field representation in the bowl-mouth plane parameter domain. For cases in which staple foods are occluded or even invisible, the staple-food state is divided into three categories according to the visible proportion: partial occlusion, severe occlusion, and invisibility [17]. Meanwhile, staple-layer height completion and supporting lower-boundary correction are introduced to prevent the volume of upper-layer foods from being overestimated. The experimental results show that the best reconstruction performance is obtained when the voxel size is set to 2 mm, the truncation distance is set to 4 V, and 100% image-ratio input is used. After the two-dimensional semantic results are back-projected onto the three-dimensional surface, the back-projection mIoU is 86.9%, the mDice is 92.8%, and the bF1 is 91.9%. For samples containing staple foods, the MAPE of staple-food weight is 9.64%, indicating that the proposed reconstruction and layered compensation strategy can provide a basis for subsequent volume and nutritional composition prediction.

#### 2.2.4. Height-Field-Based Volume Segmentation by Food Category

A height-field-based volume segmentation method was developed to partition the volume at the food-category level. First, the surface reconstructed by RGB-TSDF was projected onto the bowl-mouth plane parameter domain, forming a height field that describes the uppermost visible food surface, while the bowl instance model provided the corresponding reference height of the bowl bottom or inner wall within the same parameter domain. By mapping the top-view semantic labels onto this height field, the occupied regions of different food categories could be determined within a unified geometric representation. For the staple-food layer and the upper-layer foods, the upper and lower boundaries were defined separately according to their spatial relationships, and the volume of each category was then calculated through category-level height-field integration [18]. The experimental results show that, after the two-dimensional semantic results were mapped onto the three-dimensional surface, mIoU, mDice, and bF1 reached 86.9%, 92.8%, and 91.9%, respectively. For single-ingredient and multi-ingredient foods, the MAPE values of the corresponding weights obtained from volume estimation were 9.9% and 11.0%, respectively.

#### 2.2.5. Prediction of Nutritional Composition in Food

The category-level volume estimates were converted into mass values according to the density parameters of the corresponding food categories, and these mass values were then combined with a nutritional composition table per unit mass to calculate the Calories, Fat, Carbohydrate, and Protein contents of each food category [19]. The experimental results show that the MAPE values for whole-bowl Calories, Fat, Carbohydrate, and Protein prediction were 13.23%, 18.51%, 14.18%, and 13.35%, respectively. These results indicate that the proposed method can effectively bridge volume estimation and nutritional composition prediction, providing a feasible approach for nutritional analysis of multi-category bowl-based foods.

## 3. Methods

### 3.1. Food Semantic Segmentation Method

#### 3.1.1. Overall Introduction

**Figure 2:**
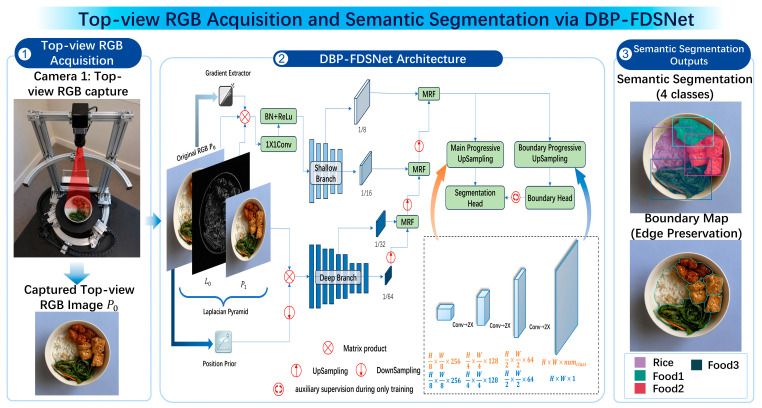
Food semantic segmentation workflow and DBP-FDSNet model architecture diagram.

In this study, we develop an improved DBP-FDSNet for food semantic segmentation on the basis of the original FDSNet. The original FDSNet combines a Laplacian pyramid, a deep–shallow dual-branch backbone, a multi-scale relationship-aware feature fusion module (MRF), and a progressive upsampling structure, which enables the model to improve food image segmentation accuracy while maintaining computational efficiency. However, in the top-view bowl-served food scenario considered in this study, food boundaries are often prone to adhesion, local texture details may be insufficient, and the spatial distribution of food regions is influenced by the bowl structure. To better adapt the model to these characteristics, a detail-enhanced shallow branch, a boundary-assisted supervision branch, and a position encoding module were introduced. These components strengthen the model’s ability to represent fine-grained contours, category boundary regions, and scene-level structural information, thereby providing more accurate semantic masks for subsequent 3D volume estimation. The food semantic segmentation workflow and the model structure of DBP-FDSNet are shown in Figure 2.

#### 3.1.2. Original FDSNet Model

The original FDSNet is a deep–shallow dual-branch model designed for food image semantic segmentation [20]. It aims to reduce computational cost by using lower-resolution input images while still maintaining reliable segmentation accuracy. This model, through Laplacian pyramid decomposition, separates the input image into a downsampled RGB image and a high-frequency residual image. The deep backbone branch takes the low-resolution RGB image as its input and mainly extracts high-level semantic information, whereas the shallow backbone branch uses the high-frequency residual image to supplement local spatial details such as food edges and textures. With this dual-branch design, FDSNet can combine semantic representation with fine-grained detail information, which helps preserve segmentation accuracy while keeping the model computationally efficient.

**Figure 3:**
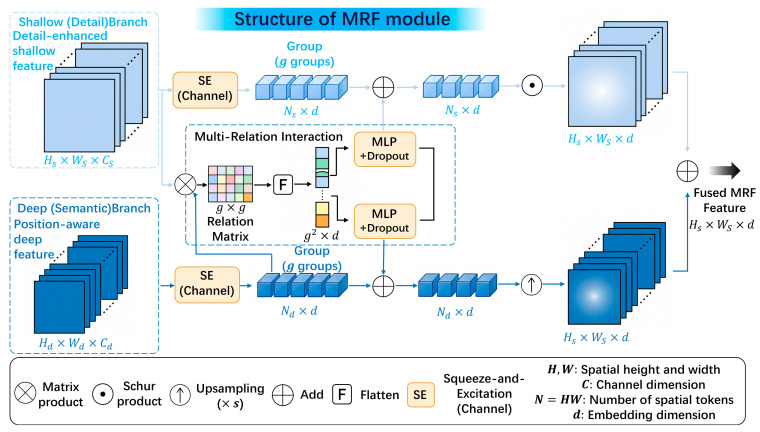
Structural diagram of the MRF module.

In the feature fusion stage, the model uses the MRF module to integrate semantic features from different scales and hierarchical levels. The module applies channel attention to learn channel-wise weights from both high-resolution and low-resolution features, and then strengthens the interaction between deep semantic information and shallow detail information through grouping operations and interactive mapping. In this way, it enables more effective multi-scale feature fusion while preserving the complementary information provided by the two branches. The fused high-resolution features are then gradually restored to the original image size through progressive upsampling, which helps reduce the noise and instability that may be introduced by one-step large-scale upsampling. The structural diagram of the MRF module is shown in Figure 3.

However, when applied to the fixed top-view food segmentation task considered in this study, the original FDSNet still shows limitations in several aspects, particularly in fine-grained boundary fusion and discrimination, the effective use of spatial information, and its compatibility with subsequent 3D food volume prediction. These limitations become more evident in bowl-served food scenarios, where adjacent food regions often adhere to each other, and the spatial distribution of food is strongly affected by the container structure. Therefore, on the basis of FDSNet, we introduce detail-enhanced input, boundary-assisted supervision, and position encoding to construct the improved DBP-FDSNet model [21].

#### 3.1.3. DBP-FDSNet

When the original FDSNet model is directly applied to the semantic segmentation of top-view images of bowl-based foods, its adaptability to this specific scenario remains limited. In bowl-based meals, different food categories often come into contact with one another, overlap, or adhere around their boundaries, and fine-grained contours become even more difficult to distinguish accurately when the colors and textures of adjacent foods are similar. If only the high-frequency residual information obtained from the original Laplacian pyramid is used, local edge variations and weak boundary structures cannot be sufficiently described. At the same time, the original model is mainly optimized for pixel-level semantic classification, while insufficient constraints are imposed on food contours and category transition regions, which can easily lead to blurred boundaries or mask adhesion between neighboring food items. In this study, however, a relatively fixed top-view acquisition setting is adopted, and a bowl model is further used in the subsequent processing stage. Under this setting, the food region, bowl rim, and background region exhibit certain spatial regularities in the image, yet these spatial distribution characteristics and container-structure cues are not fully exploited by the original model.

To address the above issues, DBP-FDSNet was developed in this study on the basis of the dual-branch structure and multi-scale feature fusion framework of FDSNet, with several improvements introduced for the top-view segmentation task of bowl-based foods.

First, a detail-enhanced input composed of the original image $P_{0}$, the high-frequency residual map $L_{0}$, and the gradient magnitude map $G_{0}$ was incorporated into the shallow branch, allowing the model to make use of appearance information together with high-frequency details and edge cues, thereby improving its sensitivity to local texture variations and food boundaries. Second, a boundary-assisted supervision branch was added after the top-level fused feature $F_{top}$, so that the model could be guided during training not only by the classification of food regions but also by boundary locations and contour continuity, which helps improve the segmentation of adhesion regions between adjacent food items. Third, spatial encoding information, including $X_{coord}$, $Y_{coord}$, and $D_{bowl}$, was introduced at the input stage of the deep branch, enabling the network to exploit the relatively stable spatial distribution patterns in bowl-based scenes, particularly the relationship between food regions, the bowl center, and the bowl boundary.

**Figure 4:**
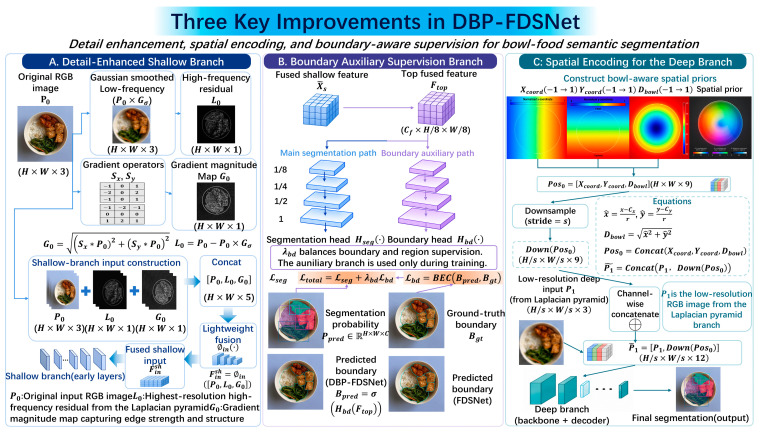
The schematic diagrams of the three key improvements in DBP-FDSNet.

Through these improvements, DBP-FDSNet can generate food semantic masks with clearer boundaries, more continuous regions, and better structural consistency, thereby providing more reliable segmentation results for subsequent two-dimensional semantic mapping, three-dimensional volume segmentation, and nutritional composition estimation. The specific improvements made to each component of the model are explained in greater detail below. Figure 4 presents the overall architecture of DBP-FDSNet and the structure of the MRF module.

1. Detail-Enhanced Shallow Branch

The original FDSNet model adopts a dual-branch architecture consisting of deep and shallow branches, where the deep branch is used to extract high-level semantic information, while the shallow branch supplements local texture and spatial details by using the high-frequency residuals obtained from Laplacian pyramid decomposition [22]. This design can alleviate, to some extent, the loss of boundary information caused by low-resolution input. However, in top-view images of bowl-based foods, different food categories often come into contact with one another, and the differences in color and texture between some food items can be relatively weak. Relying only on the high-frequency residual $L_{0}$ makes it difficult to describe weak boundaries and fine contours, and local noise may also be mistakenly interpreted as category boundaries, which can lead to discontinuous boundaries or under-segmentation.

To address this issue, a detail-enhanced input is introduced into the shallow branch in this study, so that the model can jointly use the original appearance information, high-frequency structural information, and explicit gradient-based edge information [23]. Let the original input image be denoted as $P_{0}\in R^{H\times W\times 3}$, and let the high-frequency residual map at the highest resolution obtained after Laplacian pyramid decomposition be denoted as $L_{0}\in R^{H\times W\times 1}$. Here, $P_{0}$ preserves the color, texture, and illumination information of the image, whereas $L_{0}$ emphasizes local high-frequency variations. The gradient magnitude map $G_{0}$ is then calculated from $P_{0}$. If $S_{x}$ and $S_{y}$ denote the gradient operators in the horizontal and vertical directions, respectively, and ∗ denotes the convolution operation, $G_{0}$ can be expressed as shown in Equation (1):

(1) $$G_{0}=\sqrt{{\left(S_{x}*P_{0}{)}^{2}+(S_{y}*P_{0}\right)}^{2}}$$

In practice, $P_{0}$ is first converted into a grayscale image or luminance component before the gradient is computed, so as to reduce the computational cost; therefore, $G_{0}\in R^{H\times W\times 1}$. Compared with $L_{0}$, $G_{0}$ can more directly reflect variations in pixel intensity and edge strength. Subsequently, $P_{0}$, $L_{0}$, and $G_{0}$ are concatenated along the channel dimension to form the detail-enhanced input of the shallow branch, denoted as $X_{s}=Concat\left(P_{0},L_{0},G_{0}\right)$. Since direct concatenation increases the number of input channels, a lightweight input fusion module $\phi_{s}\left(\cdot \right)$ is further used in this study to perform channel compression and local feature extraction on $X_{s}$, producing the shallow detail feature $F_{s}=\phi_{s}\left(X_{s}\right)$. Here, $\phi_{s}\left(\cdot \right)$ consists of small-kernel convolution, normalization, and nonlinear activation, and is designed to fuse multiple types of detail information without noticeably increasing the complexity of the network. Through this design, the improved shallow branch no longer relies only on a single high-frequency residual, but instead integrates information from the original image, high-frequency residuals, and gradient-based edges, thereby providing richer local cues for adjacent food adhesion regions and weak boundary areas while improving the boundary clarity and regional integrity of the subsequent semantic segmentation results.

2. Boundary-Assisted Supervision Branch

The original FDSNet is mainly optimized through a semantic classification loss to produce the final segmentation result. Although this allows the model to learn the approximate regional distribution of different food categories, the constraints imposed on food contours and category transition regions remain relatively weak. In images of bowl-based foods, adjacent food items often adhere to one another, and their boundaries may appear blurred; therefore, if only region-level classification is used, the resulting masks are prone to unclear edges or insufficient separation between neighboring categories [24]. To address this problem, a boundary-assisted supervision branch is introduced into DBP-FDSNet, so that the model is encouraged during training to attend not only to semantic region partitioning but also to boundary localization, thereby improving the contour clarity and spatial continuity of food masks.

This branch is connected after the top-level fused feature $F_{top}$. Let $\phi_{b}\left(\cdot \right)$ denote the boundary-assisted branch. After lightweight convolutional mapping and upsampling, the feature map is restored to the same spatial resolution as the input image, and a single-channel boundary probability map is then generated through the Sigmoid function, as shown in Equation (2):

(2) $$\hat{B}=\sigma \left(\phi_{b}\left(F_{top}\right)\right)$$

where $\hat{B}\in R^{H\times W\times 1}$ denotes the predicted boundary map. A pixel value closer to 1 indicates that the corresponding location is more likely to belong to a food contour or a category transition region. It should be noted that $\hat{B}$ is not involved in the final category prediction; instead, it is used as an auxiliary supervision signal during training, so that the shared features can be guided to pay greater attention to boundary regions.

The boundary label B can be automatically generated from the existing semantic segmentation annotation Y, and no additional manual annotation is required. Specifically, neighborhood differencing and morphological gradient operations are applied to the semantic label map, and pixels where category changes occur are marked as boundary regions. This process can be expressed as $B=G\left(Y\right)$, where $G\left(\cdot \right)$ denotes the boundary extraction operator. The generated boundary label includes both the external contours between food and the background or bowl wall, as well as the internal boundaries between different food categories.

During training, the boundary-assisted branch constrains the consistency between $\hat{B}$ and B through a boundary prediction loss. Since boundary regions usually account for only a small proportion of the image, while both pixel-level prediction stability and the overlap degree of boundary regions need to be considered [25], *binary cross-entropy loss* and Dice *loss* are combined in this study, and the *boundary loss* is defined as shown in Equation (3):

(3) $$L_{b}=L_{BCE}\left(\hat{B},B\right)+L_{Dice}\left(\hat{B},B\right)$$

The final training objective is jointly composed of the *main semantic segmentation loss* and the *boundary-assisted loss*, which can be written as $L=L_{seg}+\lambda_{b}L_{b}$, where $L_{seg}$ denotes the main segmentation loss and $\lambda_{b}$ is used to control the weight assigned to the *boundary-assisted loss*. With this design, the model is able to learn category-level semantic information while also capturing food boundary shapes, contour continuity, and category transition locations more effectively. The boundary-assisted branch participates in backpropagation only during the training stage, whereas the main segmentation branch is still used during inference to generate the final semantic masks, and therefore, no noticeable increase in inference complexity is introduced.

3. Incorporation of Spatial Encoding

In the top-view images of bowl-based foods used in this study, food regions are not randomly distributed, but are clearly constrained by the bowl position, the imaging setup, and the container structure. Since the camera is fixed directly above the bowl, the bowl is usually located near the center of the image, with most food items distributed inside the bowl, whereas regions close to the image boundary are more likely to correspond to the bowl wall, tabletop, or background [26]. The original FDSNet mainly relies on color and texture features for semantic inference, and therefore does not fully exploit this relatively stable spatial distribution pattern. To address this limitation, spatial encoding is introduced at the input stage of the deep branch in DBP-FDSNet, so that deep semantic features can incorporate both pixel-level positional information and bowl-structure cues during feature extraction.

Specifically, three types of spatial features are constructed in this study: the horizontally normalized coordinate map $X_{coord}$, the vertically normalized coordinate map $Y_{coord}$, and the distance map $D_{bowl}$, which describes the distance from each pixel to the bowl center or the valid region inside the bowl. Among them, $X_{coord}$ and $Y_{coord}$ are used to represent the relative position of each pixel in the image plane, while $D_{bowl}$ is used to describe the spatial relationship between each pixel and the bowl center, bowl wall, or bowl rim boundary. Let the image size be H×W, the pixel location be denoted as $\left(i,j\right)$, and the bowl center be denoted as $\left(x_{c},y_{c}\right)$. The coordinate maps and distance map can then be expressed as shown in Equations (4) and (5), respectively:

(4) $$X_{coord}\left(i,j\right)=\frac{j}{W-1},Y_{coord}\left(i,j\right)=\frac{i}{H-1}$$

(5) $$D_{bowl}\left(i,j\right)=\frac{\sqrt{{\left(j-x_{c}{)}^{2}+(i-y_{c}\right)}^{2}}}{r_{bowl}}$$

where $r_{bowl}$ denotes the normalized scale factor estimated from the bowl rim region. The three types of spatial features are then concatenated along the channel dimension to obtain the spatial encoding feature, as shown in Equation (6):

(6) $$E_{pos}=Concat\left(X_{coord},Y_{coord},D_{bowl}\right)$$

Since the deep branch usually receives downsampled low-resolution input, $E_{pos}$ is resized in this study to the same scale as the input of the deep branch, yielding $E_{pos}^{d}$, which is then concatenated with the low-resolution RGB input or its corresponding feature $P_{d}$, so that $X_{d}=Concat\left(P_{d},E_{pos}^{d}\right)$, where $X_{d}$ denotes the deep-branch input after spatial encoding has been incorporated. Through this design, when high-level semantic information is extracted by the deep branch, the model is able to use not only the color and texture cues of food, but also the relative position of each pixel within the bowl and the geometric relationship imposed by the container. As a result, valid food regions inside the bowl, the bowl rim, and background regions can be distinguished more robustly, while mis-segmentation caused by local texture similarity or interference from the bowl wall can be reduced [27].

#### 3.1.4. Loss Definition of DBP-FDSNet and Its Connection to Subsequent Outputs

In this section, we further clarify the training objective of DBP-FDSNet and its connection with the subsequent 3D volume prediction process. Since the core task of the model remains food semantic segmentation, the main segmentation branch takes pixel-level category prediction as its primary optimization objective. Given the clear morphological differences among staple foods, single-ingredient foods, and multi-ingredient foods in the dataset, the main segmentation loss is designed to consider both pixel-level classification accuracy and region-overlap consistency. During training, DBP-FDSNet is jointly guided by semantic region supervision and boundary structure supervision [28], where the former strengthens category discrimination and the latter improves the contour quality of the predicted masks. Because the boundary ground truth is automatically derived from existing semantic annotations, this design improves boundary representation without introducing additional labeling cost.

During inference, DBP-FDSNet retains only the main segmentation branch, which generates a category probability map and a final semantic label map with the same spatial size as the input image [29]. The resulting 2D semantic label map is used not only for evaluating food semantic segmentation performance, but also as the semantic input for the subsequent 3D analysis pipeline. In this pipeline, the segmentation results are mapped onto the 3D food surface reconstructed by RGB-TSDF or onto the height-field parameter domain, so that the occupied regions of different food categories inside the bowl can be determined. Together with the bowl instance model, the staple-food occlusion compensation strategy, and category-level height-field integration, these mapped semantic regions further support the volume estimation of each food category.

Therefore, within the overall workflow of this study, DBP-FDSNet serves as the front-end module linking 2D semantic segmentation to 3D category-level volume prediction. The accuracy, boundary clarity, and regional integrity of its output masks directly influence the reliability of subsequent semantic mapping, category-level volume segmentation, weight estimation, and nutritional composition prediction.

### 3.2. Three-Dimensional Food Volume Prediction

#### 3.2.1. Data Collection and Coordinate System Establishment

In our previous research, we extracted the geometric parameters of bowl-type containers, completed their 3D reconstruction and capacity calculation, and constructed an instance database, thereby providing reusable container structure models for subsequent food volume estimation [30]. Building on this foundation, this chapter further investigates 3D volume prediction for dishes served in bowls and develops a data acquisition system composed of two types of cameras. The top-view camera is fixed directly above the bowl to capture top-view images and provide semantic segmentation results, while the surrounding camera is placed above the bowl and moves from the left side to the right side through the top position, continuously collecting RGB images and depth information for multi-view 3D surface reconstruction [31].

To keep multi-view fusion reconstruction and volume integration within a consistent geometric space, a bowl coordinate system $\left\{B\right\}$ is established based on the registered bowl instance model. The center of the bowl bottom is taken as the origin, with the Z-axis pointing vertically upward and the X- and Y-axes lying on the tabletop plane. The mapping between image pixels and physical scale is also obtained to provide a unified scale reference. For any 3D point $X_{B}$ and image point $\tilde{u}$, the imaging relationship is expressed in Equation (7):

(7) $$s\tilde{u}=K\left[R|t\right]X_{B}$$

where s denotes the scale factor, and $\tilde{u}=[u,v,1{]}^{T}$ denotes the homogeneous pixel coordinate in the image, with u and v representing the horizontal and vertical pixel coordinates on the image plane, respectively. K is the camera intrinsic matrix containing the focal length parameters and principal point coordinates, while $\left[R|t\right]$ represents the camera extrinsic parameters relative to the bowl coordinate system, where R is a 3×3 rotation matrix and t is a 3×1 translation vector. $X_{B}=[x_{B},y_{B},z_{B},1{]}^{T}$ denotes the homogeneous coordinate of a 3D point in the bowl coordinate system $\left\{B\right\}$. Based on this relationship, the intrinsic and distortion parameters of the two cameras are estimated separately. With the calibration board placed at the bowl position as an intermediate reference, the extrinsic parameters from each camera to the bowl coordinate system are then obtained, as shown in Equation (8):

(8) $$T_{B\leftarrow C_{i}}=\left[\begin{array}{cc} R_{i} & t_{i}\\ 0 & 1 \end{array}\right]$$

where $T_{B\leftarrow C_{i}}$ denotes the rigid transformation from the i-th camera coordinate system to the bowl coordinate system. Here, i=1 corresponds to the top fixed camera, whereas i=2 corresponds to the surrounding RGB-D camera. The terms $R_{i}$ and $t_{i}$ represent the rotation and translation parameters of the camera relative to the bowl coordinate system, respectively. For the surrounding camera, the frame-wise pose $T_{B\leftarrow C_{2}}\left(t\right)$ is obtained by combining the mechanical trajectory with visual correction. In addition, RGB-D color-depth registration and depth error correction are performed to reduce systematic deviations caused by changes in imaging distance and field-of-view position.

The acquisition device used in this study was designed as an experimental platform for methodological validation, rather than as a final consumer-grade dietary monitoring device. The top-view RGB camera was used to obtain two-dimensional semantic segmentation information, while the surrounding RGB-D cameras were employed to provide multi-view depth information. In addition, the slide rail and turntable were used to maintain stable camera poses, acquisition trajectories, and illumination conditions. This configuration helps reduce interference caused by handheld imaging, natural light variations, and viewpoint uncertainty, thereby allowing the proposed three-dimensional reconstruction and volume estimation pipeline to be evaluated more accurately. Nevertheless, this also means that a gap remains between the current system and real dining scenarios, and further simplification of the hardware structure, together with improved system robustness, will be required before the method can be directly applied to ordinary mobile devices or open dining-table environments.

#### 3.2.2. Multi-View Fusion Reconstruction of the 3D Surface

This subsection aims to reconstruct the visible 3D surface of the food inside the bowl and establish a multi-view fusion pipeline based on surrounding RGB-D images. The surrounding camera continuously captures RGB-D images at 30 Hz for 30 s, producing approximately 900 RGB-D frames, each with a device-provided pose. Rather than retaining only a small number of discrete keyframes, this study uses the complete trajectory sequence as the fusion input. The vertical-view frame captured when the camera passes directly above the bowl center is retained as the main reference frame; although it shares the same imaging direction as Camera 1, it is acquired at a different height. The remaining frames are incorporated into the fusion process according to their temporal order and trajectory positions, allowing the observation trajectory of the surrounding camera to cover the food surface from the upper-left side, through the central upper position, to the upper-right side.

For each frame, the valid-depth ratio within the bowl region is calculated, and frames with a ratio lower than 85% are discarded. The remaining RGB-D frames, together with their corresponding poses, are then transformed into the bowl coordinate system for subsequent Truncated Signed Distance Function (TSDF) voxel integration and surface reconstruction. The initial poses of the keyframes captured by the surrounding camera are provided by the device, including the slide-rail position and camera attitude. By combining this information with the unified relationship between the bowl and the device described in Section 3.2.1, the pose of the surrounding camera at the k-th frame relative to the bowl coordinate system $\left\{B\right\}$ can be expressed as Equation (9):

(9) $$T_{B\leftarrow C_{2}}^{k}=\left[\begin{array}{cc} R_{k} & t_{k}\\ 0 & 1 \end{array}\right]$$

where $R_{k}$ denotes the rotation matrix of the camera relative to the bowl coordinate system in the k-th frame, and $t_{k}$ denotes the corresponding translation vector. For multi-view fusion of RGB-D images, this study adopts a TSDF-based method [32]. By accumulating depth observations from multiple frames, this voxel-based reconstruction method can gradually suppress single-frame depth noise, local holes, and outliers, while forming a continuous and smooth implicit surface near the voxel zero-level set. The reconstructed surface is then recovered from the zero-isosurface. In the TSDF formulation, let x denote the voxel center, and let $d_{k}\left(x\right)$ denote the projected signed distance corresponding to the observation in the k-th frame. The cumulative update formula is given in Equation (10):

(10) $$D_{k}\left(x\right)=\frac{W_{k-1}\left(x\right)D_{k-1}\left(x\right)+w_{k}\left(x\right)d_{k}\left(x\right)}{W_{k-1}\left(x\right)+w_{k}\left(x\right)},W_{k}\left(x\right)=W_{k-1}\left(x\right)+w_{k}\left(x\right)$$

where $D_{k}\left(x\right)$ denotes the TSDF value of the voxel after the k-th update, $W_{k}\left(x\right)$ denotes the accumulated weight, and $w_{k}\left(x\right)$ denotes the current observation weight. This formulation can be regarded as a recursive weighted averaging process based on observation confidence, which helps reduce the influence of noise on surface details. In preliminary experiments, we found that the dense voxel representation of TSDF required relatively high memory consumption. Therefore, RGB-TSDF was adopted in the subsequent reconstruction, as it is more suitable for high-precision small-object reconstruction under the surrounding-view acquisition setting used in this study [33]. RGB-TSDF manages voxels through a grid-octree sparse voxel structure and a hard-coded indexing mechanism, allocates high-resolution voxels only in observed regions, and introduces bilinear interpolation during depth-map fusion to improve the accuracy of local voxel updates.

In this study, all valid RGB-D frames along the complete trajectory are used as fusion inputs. Let $T_{B\leftarrow C_{2}}^{k}$ denote the camera pose of the k-th frame, $Z_{k}\left(u,v\right)$ denote the corresponding depth map, and K denote the camera intrinsic matrix. The local 3D point associated with pixel $\left(u,v\right)$ can then be calculated using Equation (11):

(11) $$p_{k}(u,v)=Z_{k}(u,v)K^{-1}[u,v,1{]}^{T}$$

The resulting point is then transformed into the bowl coordinate system through $T_{B\leftarrow C_{2}}^{k}$ for subsequent processing. Since the bowl serves as the container in this 3D food reconstruction task, spatial cropping based on the bowl instance model is performed before voxel updating, and only valid observations within the bowl cavity and near the bowl rim are retained.

During fusion, RGB-TSDF constructs sparse active voxel blocks from the depth map, with voxel nodes allocated only near the food surface and the inner wall of the bowl. Based on the interpolated depth-map value, the signed distance between each voxel center and the currently observed surface is calculated, and TSDF updating is then performed within the truncated narrow band.

To ensure the stability of subsequent processing, a triangular mesh is extracted from the TSDF zero-level set, $D\left(x\right)=0$, using the Marching Cubes method after RGB-TSDF fusion. This step produces the initial 3D model of the visible inner bowl wall and food surface, whose reconstruction quality is then evaluated from the following four aspects: 1. Geometric integrity: The reconstructed surface is checked for continuity and possible holes. 2. Floating fragments and pseudo-surfaces: Isolated small connected components are identified and removed. 3. Scale consistency: The mesh is verified to ensure that it remains within the physically reasonable spatial range of the bowl interior. 4. Projection consistency: The mesh is reprojected onto the original image to examine its alignment with the depth boundaries and the inner contour of the bowl.

**Figure 5:**
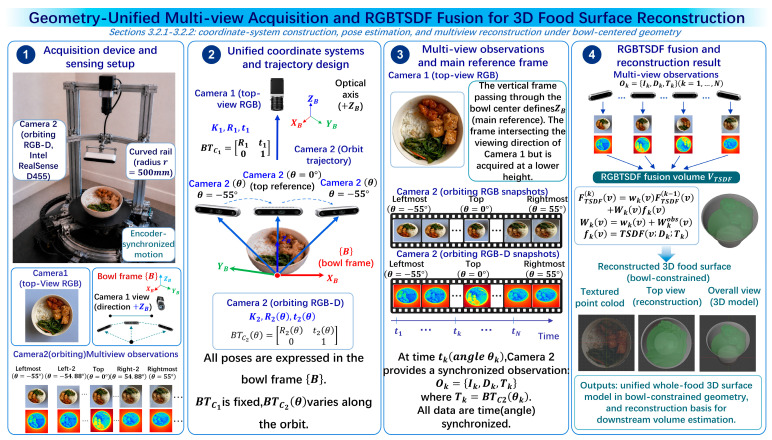
Workflow of data acquisition, coordinate system construction, and multi-view 3D fusion-based food surface reconstruction.

After this screening process, the reconstructed model is used as the final surface model for subsequent 3D segmentation and volume calculation. Figure 5 illustrates the workflow of data acquisition, coordinate system construction, and multi-view 3D fusion-based food surface reconstruction.

#### 3.2.3. Occlusion and Staple-Food Invisibility Strategy

In bowl-served food scenarios, different food items usually present clear spatial stacking relationships. Staple foods, especially Rice, are often located at the bottom of the bowl or in a lower layer, where they may be partially occluded or even completely covered by upper-layer single-ingredient or multi-ingredient foods. If volume integration relies only on the visible surface, the staple-food volume may be underestimated, while the volume of upper-layer foods may be overestimated. To address this issue, a staple-food occlusion determination and layered compensation strategy is introduced before geometry-constrained 3D segmentation and volume calculation [34].

The 20 food categories are divided into three groups: staple foods, single-ingredient foods, and multi-ingredient foods. The staple-food category contains Rice. The single-ingredient food category includes Roasted potato, Spring roll, Grilled skewer, Sausage, Egg tart, Cold rice noodles, Corn, Roasted pumpkin, Salad, and Braised tofu, while the multi-ingredient food category includes Chicken with potatoes, Braised pork belly, Spicy diced chicken, Pork with celery, Shredded pork in garlic sauce, Sweet and sour fish fillet, Scrambled eggs with tomato, Shredded potato with celery, and Pork with black fungus.

The dataset used in this study does not consist solely of traditional Chinese foods. In addition to typical Chinese meal items such as Rice, Braised pork belly, Spicy diced chicken, and Shredded pork in garlic sauce, it also includes food categories that are commonly found in different dietary contexts, such as Sausage, Egg tart, Corn, Roasted pumpkin, Salad, and Spring roll. Therefore, the dataset shows a certain degree of diversity in terms of food appearance, morphological structure, and nutritional composition, which allows it to be used for evaluating the proposed method’s ability to estimate volume and predict nutritional content in multi-category bowl-based meal scenarios. Nevertheless, the applicable scope of this dataset is still limited to bowl-based meals, and it cannot be taken as evidence that the proposed method already covers all dietary cultures or cooking styles.

For single-ingredient and multi-ingredient foods, volume integration is mainly based on their visible 3D surfaces, whereas staple foods require an additional strategy for occlusion compensation and invisibility determination. To quantitatively describe the degree of staple-food occlusion, the visible proportion of staple food is defined as $R_{\mathrm{vis}}=\frac{|\Omega_{r}^{\mathrm{vis}}|}{|\Omega_{r}|}$, where $\Omega_{r}^{\mathrm{vis}}$ denotes the directly observable staple-food region in the top-view semantic segmentation result, $\Omega_{r}$ denotes the estimated overall occupied region after completion, and ∣⋅∣ denotes the region area, namely the number of pixels. According to $R_{\mathrm{vis}}$, the staple-food state is divided into the following three cases:

(1) Partial occlusion of staple food: When the staple-food label is detected in the semantic segmentation result and the visible proportion satisfies $R_{\mathrm{vis}}\geq 40\%$, the staple food is regarded as partially occluded. In this case, a sufficiently visible staple-food region is still present in the top-view image, which can be used to estimate its reference height. Let $H\left(x,y\right)$ denote the uppermost visible height field inside the bowl. The reference height of the staple food is then calculated as the average height of the visible staple-food region, as shown in Equation (12):

(12) $${\overset{ˉ}{H}}_{r}=\frac{1}{|\Omega_{r}^{vis}|}\sum_{(x,y)\in \Omega_{r}^{vis}}H(x,y)$$

For regions that are occluded by upper-layer foods but still belong to the staple-food area, the staple-food layer is assumed to be locally continuous, and its completed upper-surface height is set as $H_{r}\left(x,y\right)={\overset{ˉ}{H}}_{r}$, where $H_{r}\left(x,y\right)$ denotes the completed upper-surface height of the staple food. This strategy helps recover partially occluded staple-food regions and avoids underestimating the volume when only the exposed staple-food area is considered.

(2) Severe occlusion of staple food: When the staple-food label is still detected in the semantic segmentation result but the visible proportion satisfies $5\%<R_{\mathrm{vis}}<40\%$, the staple food is regarded as severely occluded. In this case, only a small portion of the staple-food region remains visible, so estimating its volume directly from the exposed area would introduce considerable deviation. The visible staple-food region is therefore still used to estimate the reference height, while the completion of the occupied staple-food region relies more on the spatial constraints inside the bowl and the distribution of upper-layer foods. The staple-food volume under this condition can be expressed as Equation (13):

(13) $$V_{r}=\iint_{\Omega_{r}}\left(H_{r}(x,y)-H_{bowl}(x,y)\right)\;dxdy$$

where $H_{\mathrm{bowl}}\left(x,y\right)$ denotes the reference height of the bowl bottom or inner wall provided by the bowl model, and $\Omega_{r}$ denotes the completed occupied region of the staple food. For single-ingredient or multi-ingredient foods located above the staple-food layer, the lower boundary is not directly defined by the bowl-bottom height; instead, it is set to the larger value between the completed staple-food layer height and the bowl-model height, namely $H_{\mathrm{bottom}}^{c}\left(x,y\right)=\max\left(H_{\mathrm{bowl}}\left(x,y\right),H_{r}\left(x,y\right)\right)$. The volume of the upper-layer food category c can therefore be calculated as shown in Equation (14):

(14) $$V_{c}=\iint_{\Omega_{c}}\left(H_{top}(x,y)-H_{bottom}^{c}(x,y)\right)\;dxdy$$

This strategy prevents the spatial volume occupied by the staple-food layer from being mistakenly included in the volume of upper-layer foods, thereby making category-level volume estimation more reasonable.

(3) Invisibility of staple food: When no staple-food label is detected in the semantic segmentation result and the visible proportion satisfies $0\leq R_{\mathrm{vis}}\leq 5\%$, the staple food is regarded as invisible in the current top-view image. However, this does not necessarily mean that the sample contains no staple food, since the staple-food layer may be completely covered by upper-layer foods. To handle this situation more appropriately, the invisible case is further divided into two subcases.

(a) The staple food is completely covered but actually present: If the semantic segmentation module detects no staple-food label. In this case, the staple food layer is completely invisible in the top-view image, and its spatial extent cannot be directly obtained by the system from the visible semantic labels alone. Therefore, this condition is defined in the present study as a semi-automatic fallback procedure under extreme occlusion, rather than as a fully automated process for staple food volume estimation. It should be noted that the user-provided staple food mass $m_{r}^{user}$ is used only as auxiliary information for low-visibility samples, with the purpose of preventing the space actually occupied by the staple food from being incorrectly assigned to upper-layer food items. For samples in which the staple food is partially or severely occluded, the system still mainly relies on the visible staple food region, bowl model constraints, and height-field continuity to complete the estimation automatically.

Based on the staple-food density $\rho_{r}$, the corresponding volume can be inversely calculated as $V_{r}^{\mathrm{user}}=\frac{m_{r}^{\mathrm{user}}}{\rho_{r}}$. The inner-wall contour of the bowl instance model is then used to establish the relationship between the internal bowl volume and height, yielding the bowl volume–height function $C_{\mathrm{bowl}}\left(h\right)$. By solving $C_{\mathrm{bowl}}\left(h_{r}\right)=V_{r}^{\mathrm{user}}$, the estimated height $h_{r}$ of the completely invisible staple-food layer can be obtained. In this case, the staple-food layer height is set as $H_{r}\left(x,y\right)=h_{r}$, which serves as the supporting lower boundary for upper-layer single-ingredient and multi-ingredient foods. Thus, when staple food is completely covered, the volumes of other food categories are calculated between the visible surface and the staple-food layer rather than between the visible surface and the bowl bottom, preventing the space occupied by staple food from being mistakenly assigned to upper-layer foods. This treatment can preserve the geometric plausibility of volume partitioning when the staple food is completely invisible; however, it also indicates that manual assistance is still required in this case, which represents a current limitation of the proposed pipeline for fully automated application. In future work, methods for automatically determining the presence of staple foods and estimating the volume of hidden layers will be further explored by considering the remaining space inside the bowl, the support relationship between the staple layer and upper-layer foods, and information provided by additional devices, so that the dependence on user input can be reduced.

(b) Truly no staple food: If no staple-food label is detected in the semantic segmentation result and the user confirms that the sample does not contain staple food, the staple-food layer is removed. Under this condition, single-ingredient and multi-ingredient foods are treated as being directly distributed within the bowl cavity. Since no staple-food supporting layer exists, their volume is calculated mainly from the height difference between the visible food surface and the reference height provided by the bowl model.

**Figure 6:**
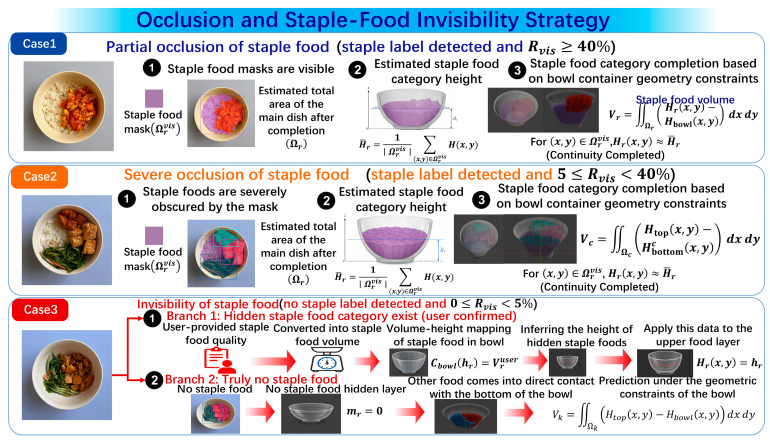
Case-specific workflow of the Occlusion and Staple-Food Invisibility Strategy.

In summary, this subsection develops a layered processing strategy for three staple-food states: partial occlusion, severe occlusion, and invisibility. When staple food remains visible, the occlusion degree is determined according to $R_{\mathrm{vis}}$, and the staple-food layer height is estimated from the visible staple-food region. When staple food is completely invisible but actually present, its layer height is inversely derived from the user-provided weight, staple-food density, and the bowl-model volume–height function. For samples that truly contain no staple food, the staple-food layer is removed, and the bowl model serves as the lower boundary for volume integration of the remaining food categories. Figure 6 illustrates the case-specific workflow of the Occlusion and Staple-Food Invisibility Strategy.

#### 3.2.4. Geometry-Constrained 3D Segmentation Model and Volume Prediction

Before 3D segmentation is performed for each food category, multiple sources of information are integrated within a unified geometric representation. These include the 3D food surface reconstructed from the sequential fusion of surrounding RGB-D camera data described in Section 3.2.2, the bowl instance model and its related geometric information retrieved from the bowl database according to the current bowl ID, the semantic segmentation results from the top-view camera, and the three-category food classification results together with the staple-food occlusion compensation information determined in Section 3.2.3. In this study, all these data are represented in the bowl coordinate system $\left\{B\right\}$, with the center of the bowl bottom defined as the reference origin. Accordingly, surface points, semantic-label mapping positions, and bowl geometric boundaries are uniformly expressed as vectors in the form of $p_{B}=[x,y,z{]}^{T}$.

During the experiments, the corresponding bowl instance model is retrieved from the bowl database and spatially aligned with the reconstructed surface in the bowl coordinate system $\left\{B\right\}$. The inner bowl wall defines the geometric boundary, the bowl bottom serves as the lower boundary, and the region within 10 mm above the bowl-rim plane is used as the upper boundary. Based on these constraints, the initial 3D surface is spatially cropped to remove the bowl body, external background regions, and abnormal surfaces penetrating into the bowl interior. This process yields an effective candidate surface that contains only the food inside the bowl and its adjacent contact regions [35], which is then used for mapping top-view semantic results into 3D space and for category-level volume integration. For this purpose, the candidate 3D food surface is converted from a mesh representation into a height-field representation defined on the bowl-rim plane. Specifically, the x-y plane of the bowl coordinate system $\left\{B\right\}$ is used as the parameter domain Ω, and the cropped 3D surface $S\subset R^{3}$ is orthogonally projected along the Z-axis. For any planar position $\left(x,y\right)\in \Omega$, the uppermost visible surface height is defined as shown in Equation (15):

(15) $$h_{t}\left(x,y\right)=\max\left\{\;z|\left(x,y,z\right)\in S\;\right\}$$

The surface height at each planar position is defined as the highest intersection between the vertical projection line and the 3D surface. Under the container-constraint strategy, the bowl instance model retrieved from the database provides the reference height function $h_{b}\left(x,y\right)$ of the inner bowl wall or bowl bottom in the same parameter domain. To facilitate parameter optimization and numerical computation, Ω is discretized into a regular grid $\left\{\left(x_{i},y_{j}\right)\right\}$, and a height matrix $H\in R^{M\times N}$ is constructed, where the $\left(i,j\right)$-th element is denoted as $H_{ij}=h_{t}\left(x_{i},y_{j}\right)$. For missing cells caused by local occlusion, such as height holes or penetrating-layer artifacts, valid neighboring height values are used for local interpolation and continuity correction, so that the height field maintains good spatial continuity within the valid bowl region.

To obtain the 2D height-associated region of a specific food category, the semantic segmentation result from the top-view camera, namely Camera 1, is mapped into 3D space. Let $M\left(u,v\right)$ denote the semantic segmentation map, where the category label of pixel $\left(u,v\right)$ is given by $c=M\left(u,v\right)$. Since the top-view camera is positioned directly above the bowl center and its imaging direction is aligned with the Z-axis of the bowl coordinate system $\left\{B\right\}$, the image pixels can be first mapped onto the bowl-rim plane parameter domain Ω. For the homogeneous image coordinate $\tilde{u}=[u,v,1{]}^{T}$, the corresponding planar point in the bowl coordinate system, $q=[x,y,1{]}^{T}$, satisfies $q∼H_{B\leftarrow I}\tilde{u}$, where $H_{B\leftarrow I}$ denotes the homography matrix from the top-view image plane to the bowl-rim plane parameter domain. Combined with the height field $h_{t}\left(x,y\right)$, the uppermost visible 3D surface point at this planar position can then be recovered, as shown in Equation (16):

(16) $$X_{t}\left(x,y\right)=\left[\begin{array}{c} x\\ y\\ h_{t}(x,y) \end{array}\right]$$

The 2D semantic label c is then assigned to the corresponding 3D surface element, forming a 3D surface model with category information. For partial staple-food occlusion, the staple-food layer height $h_{r}\left(x,y\right)$ has already been recovered in Section 3.2.3 from the partially exposed staple-food region. The staple-food label is therefore projected onto the 3D points of the staple-food layer as $X_{r}(x,y)=[x,y,h_{r}(x,y){]}^{T}$, while the labels of the overlying single-ingredient or multi-ingredient foods still correspond to the uppermost visible surface $X_{t}\left(x,y\right)$. However, assigning 2D semantic labels to spatially located 3D points in the bowl coordinate system is not sufficient for volume calculation. Category-level partitioning is still needed to define the occupied regions and hierarchical boundaries used in subsequent volume integration. Let the projected label function be $L\left(x,y\right)\in C$, where C denotes the set of food categories. The 3D segmentation result of the c-th food category is therefore represented not as an independent closed mesh in free space, but as the combination of its occupied region in the parameter domain and its corresponding height layer, as shown in Equation (17):

(17) $$S_{c}=\{\;[x,y,h_{c}(x,y){]}^{T}|(x,y)\in \Omega ,\;\;L(x,y)=c\;\}$$

where $h_{c}\left(x,y\right)$ depends on the layer to which category c belongs. For the uppermost visible foods, $h_{c}\left(x,y\right)=h_{t}\left(x,y\right)$, whereas for the staple-food layer recovered through occlusion compensation, $h_{c}\left(x,y\right)=h_{r}\left(x,y\right)$. Based on the category-level 3D segmentation results, the volume of each food category is calculated through height-field integration. Specifically, within the occupied region of category c on the bowl-rim plane parameter domain Ω, the height difference between the upper and lower boundaries is integrated to obtain the corresponding volume inside the bowl cavity, as shown in Equation (18) [36]:

(18) $$V_{c}=\iint_{\left\{(x,y)\in \Omega \;|\;L(x,y)=c\right\}}(h_{c}^{u}(x,y)-h_{c}^{l}(x,y))\;dx\;dy$$

where $h_{c}^{u}\left(x,y\right)$ and $h_{c}^{l}\left(x,y\right)$ denote the upper- and lower-boundary heights of category c at position $\left(x,y\right)$, respectively. For the staple-food layer, the upper boundary is given by the completed staple-food height field $h_{r}\left(x,y\right)$, while the lower boundary is provided by the bowl-model reference height $h_{b}\left(x,y\right)$. For single-ingredient and multi-ingredient foods located above the staple-food layer or other supporting layers, the upper boundary corresponds to the uppermost visible surface height $h_{t}\left(x,y\right)$, and the lower boundary is determined by the underlying supporting layer or the bowl-model height.

**Figure 7:**
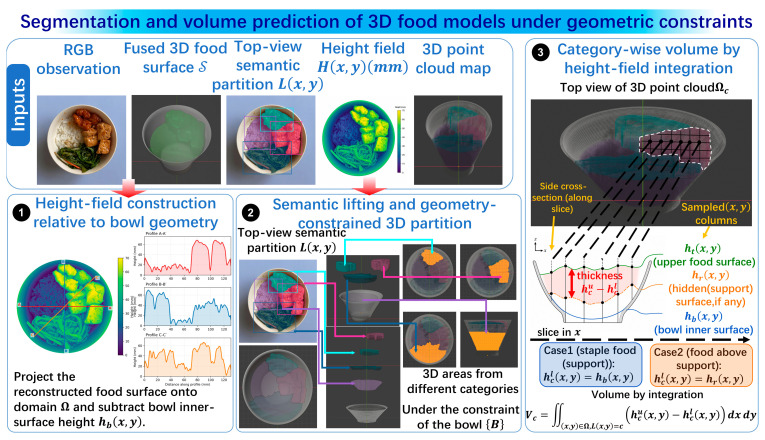
Workflow of the Geometry-Constrained 3D Segmentation Model and Volume Prediction.

At this stage, the category-level 3D segmentation results are converted into volume outputs, including the spatial distribution regions of staple foods, single-ingredient foods, and multi-ingredient foods in the bowl coordinate system, their layered height-field representations, and the estimated volume $V_{c}$ of each category. The staple-food volume is determined jointly by visible-region integration and occlusion compensation, while the volumes of single-ingredient and multi-ingredient foods are mainly obtained by integrating the height difference between the visible surface and the corresponding supporting layer. Figure 7 illustrates the workflow of the Geometry-Constrained 3D Segmentation Model and Volume Prediction.

### 3.3. Prediction of Food Nutritional Composition

At this stage, the predicted volume of each food category is used to estimate the nutritional composition of the whole bowl. A hierarchical mapping strategy is adopted, in which the category-level volume is converted into mass according to food density, and the nutrient content per 100 g of food is then used to calculate four indicators: calories, carbohydrate, protein, and fat. For the density data used in the experiments, priority was given to the INFOODS Density Database Version 2 [37], whose records are derived from literature sources, national food composition tables, and measurements conducted by the database authors. However, because this database mainly covers general foods and some Western foods, it does not provide fully matched density entries for several composite dishes included in this study. Therefore, standardized density measurements were additionally conducted for these dishes under the same image acquisition and serving conditions.

For nutritional composition data, databases such as USDA FoodData Central and EuroFIR FoodEXplorer were used [38]. For other dish categories, multi-source data were standardized and integrated according to the Specifications for Food Composition Data Expression published by the Health Data Center. For the c-th food category, let its volume and density be $V_{c}$ and $\rho_{c}$, respectively. The predicted mass is then given by $m_{c}=\rho_{c}V_{c}$. If the nutritional vector per 100 g of this category is denoted as $n_{c}=[n_{c}^{cal},n_{c}^{carb},n_{c}^{pro},n_{c}^{fat}{]}^{T}$, the predicted nutritional composition can be calculated as $y_{c}=\frac{m_{c}}{100}n_{c}$. The total nutritional composition of the whole bowl is obtained by summing the results across all categories, namely $y_{\mathrm{sum}}=\sum_{c=1}^{C}y_{c}$.

## 4. Data and Results Analysis

In this chapter, we design a series of experiments to validate the overall pipeline described above. The experiments cover the semantic segmentation results of top-view food images, ablation studies and comparative analyses of the improved model, 3D food fusion and geometric constraint processing, the role of the bowl instance model in geometric constraint construction, and the effects of different 3D fusion parameters on food categories with different structural characteristics. In the analysis, the foods are divided into three groups, namely staple foods, single-ingredient foods, and multi-ingredient foods, so that the influence of these experimental results on food volume estimation and nutritional composition prediction can be further examined.

The top-view camera, referred to as Camera 1, is a Basler ace 2 a2A1920-160ucBAS RGB camera equipped with a Basler C11-1220-12M 12 mm C-mount lens. It captures images at a resolution of 768 × 768, with the optical center positioned 700 mm above the horizontal plane and the lens aperture set between F5.6 and F8. The surrounding camera, referred to as Camera 2, uses an Intel RealSense D455 to acquire RGB-D videos. The RealSense SDK is used to align the depth images with the color images and to adjust the camera intrinsic and extrinsic parameters. Camera 2 continuously collects RGB-D data at 1280 × 720 and 30 Hz for 30 s, producing approximately 900 RGB-D frames, which are then resized to match the top-view images. By reading the encoder information from the slide rail and turntable and synchronizing it with the D455 images, the nominal pose of the surrounding camera is calculated according to geometric relationships, thereby obtaining frame-wise RGB-D poses.

As described in the previous sections, the bowl coordinate system is established with the center of the bowl bottom as the origin. The Z-axis points vertically upward, while the X- and Y-axes lie on the tabletop plane. The optical center of Camera 1 is set to $\left(0,0,700\right)\;\mathrm{mm}$. The surrounding camera moves along an arc-shaped trajectory above the bowl center, with a trajectory radius of 500 mm and a scanning angle range of $\left[-55^{\circ },55^{\circ }\right]$, while its optical axis always points toward the bowl center. When the scanning angle is $\theta =0^{\circ }$, Camera 2 is located directly above the bowl; when $\theta =\pm 55^{\circ }$, it reaches the upper-left and upper-right positions, respectively. The average angular velocity is approximately $\frac{3.66^{\circ }}{s}$, and the angular interval between adjacent frames is approximately $0.122^{\circ }$.

The experimental host is equipped with an Intel Core i9-14900K processor and an NVIDIA GeForce RTX 4090 GPU with 24 GB of memory, and the experimental platform is deployed on Ubuntu 22.04.5 LTS. The semantic segmentation network is trained using Python 3.10.20, PyTorch 2.7.0, and CUDA Toolkit 12.6.3, while visual processing and 3D reconstruction are implemented with OpenCV 4.12.0, Open3D 0.19.0, and the RGB-TSDF fusion module. PostgreSQL 17.9 is used as the main database, with PostGIS 3.6.2 extending the spatial and geometric fields, and pgAdmin 4 9.14 serving as the graphical database management tool. 

### 4.1. Semantic Segmentation Ablation Experiments and Result Analysis

In this study, we constructed a bowl-served food image dataset covering 20 food categories and 1452 sample groups. Each group contains an RGB-D video captured from surrounding viewpoints and a top-view RGB image captured above the bowl. A total of 726 different food combinations were selected, and two sample groups were generated for each combination by changing the spatial arrangement of the foods, resulting in 1452 groups. After data augmentation on the top-view RGB images, the dataset contained 4261 images with a unified resolution of 768×768, of which 10%, namely 426 images, were used as the test set. Images containing staple foods account for 65% of the dataset, corresponding to 2770 images, while images without staple foods account for 35%. In total, the dataset includes 17,684 annotated segmentation regions. Among them, staple foods account for 2770 regions, or 15.7%; single-ingredient foods account for 7245 regions, or 40.9%; and multi-ingredient foods account for 7669 regions, or 43.3% (In general, each bowl contains three to four types of food).

The image dataset was divided into training, validation, and test sets at a ratio of 7:2:1. Segmentation performance was evaluated using several commonly used metrics, including mIoU, mDice, mAcc, and bF1 (To save space, these evaluation metrics are not described in detail here, as their general definitions are not modified in this study) [39,40].

To ensure fair comparisons among different semantic segmentation models under the same experimental conditions, the training settings of DeepLabV3+ [41], BiSeNetV2 [42], SegFormer-B0 [43], FDSNet [14], and DBP-FDSNet are kept consistent. Table 1 summarizes the dataset, input image size, number of training epochs, batch size, optimizer, initial learning rate, learning rate scheduling strategy, weight decay, and warmup setting used for each model. Among these models, FDSNet and DBP-FDSNet further include a backbone-branch pretraining stage and a fine-tuning stage on the UFP dataset, which are introduced to improve feature representation and segmentation accuracy in the food semantic segmentation task.

**Figure 8:**
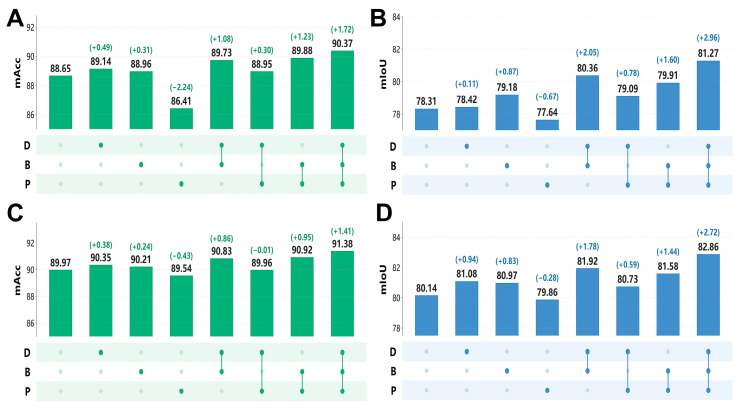
mAcc and mIoU results under different module combinations with and without pretraining. (**A**) and (**C**) correspond to mAcc without and with pretraining, respectively, while (**B**) and (**D**) correspond to mIoU without and with pretraining, respectively.

**Figure 9:**
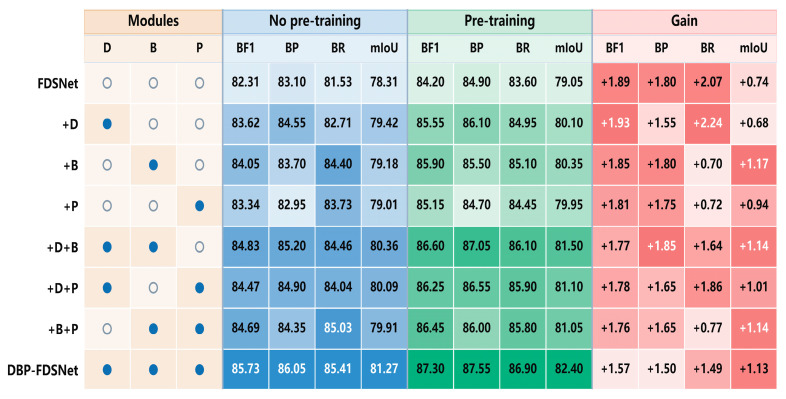
Performance heatmap illustrating the contribution of each module in the ablation experiments with and without pretraining.

**Figure 10:**
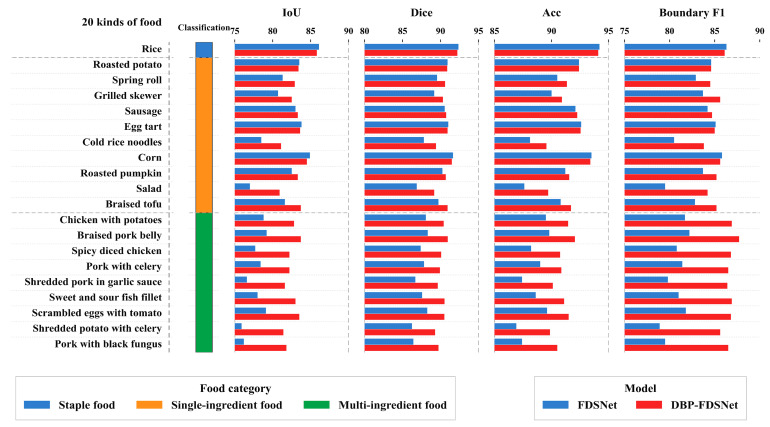
Category-wise comparison between FDSNet and DBP-FDSNet across 20 food categories in terms of IoU, Dice, Acc, and bF1.

Here, the effects of the three improved modules on segmentation performance are evaluated through ablation experiments. The original structure without these modules is used as the baseline, where *D* denotes the detail-enhanced shallow-branch input, *B* denotes boundary-assisted supervision, and *P* denotes spatial encoding. The results show that each module contributes to performance improvement when introduced individually. With *B*, the mIoU increases from 78.31% to 79.18%, and the bF1 improves by approximately 1.6%. With *D*, the mAcc increases by approximately 0.49%, while *P* improves the model’s ability to capture global spatial structure. The improvement becomes more pronounced when the modules are combined. When *D*, *B*, and *P* are introduced together, the model achieves an mIoU of 80.51%, approximately 2.2% higher than that of the original structure, while the bF1 reaches 85.73%, with an increase of about 3.5%, and the mAcc reaches 90.06%. These results suggest that the three modules complement each other in enhancing detailed feature representation, boundary clarity, and spatial information utilization, thereby improving the overall quality of food semantic segmentation. Table 2 presents the ablation results with and without UFP-based pretraining. Figure 8 shows the *mAcc* and *mIoU* results under different module combinations, both with and without pretraining. Specifically, Figure 8A,C show the mAcc results under different module combinations without and with pretraining, respectively, while Figure 8B,D show the corresponding mIoU results. Figure 9 presents the performance heatmap of the model, illustrating the contribution of each module in the ablation experiments conducted with and without pretraining. Figure 10 shows the category-wise comparison between FDSNet and DBP-FDSNet across 20 food categories in terms of IoU, Dice, Acc, and bF1.

In addition, we conducted a comparative study between DBP-FDSNet and several existing semantic segmentation methods. The compared models included U-Net (without pretraining) [44], DeepLabV3+ [41], BiSeNetV2 [42], SegFormer-B0 [43], QueryInst [45], BoundaryFormer [46] and the recently released instance segmentation models Mask2Former [47] and MaskDINO [48]. All models were trained and tested under the same 768×768 input condition. DBP-FDSNet achieved the highest segmentation accuracy, with an mIoU of 82.86%, outperforming the second-best model, Mask2Former, which achieved 82.01%. Its mDice, mAcc, and bF1 were also the highest among all compared methods. A comparative analysis of multiple models was further carried out in this study.

**Figure 11:**
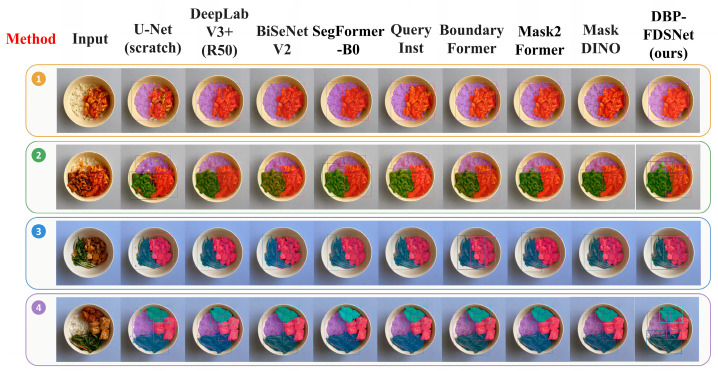
Prediction results of different semantic segmentation models on four representative test samples.

**Figure 12:**
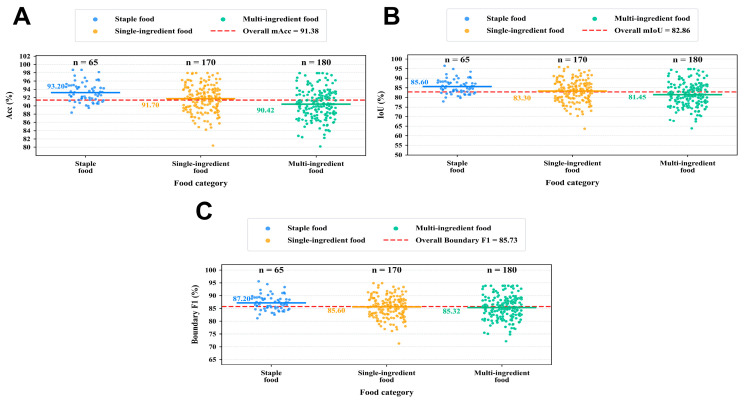
Distributions of *Acc* (**A**), *IoU* (**B**), and *bF*1 (**C**) for the segmented regions of the test set based on three major food categories across 100 randomly selected test samples; the red dashed line indicates the overall mean value of each metric.

**Figure 13:**
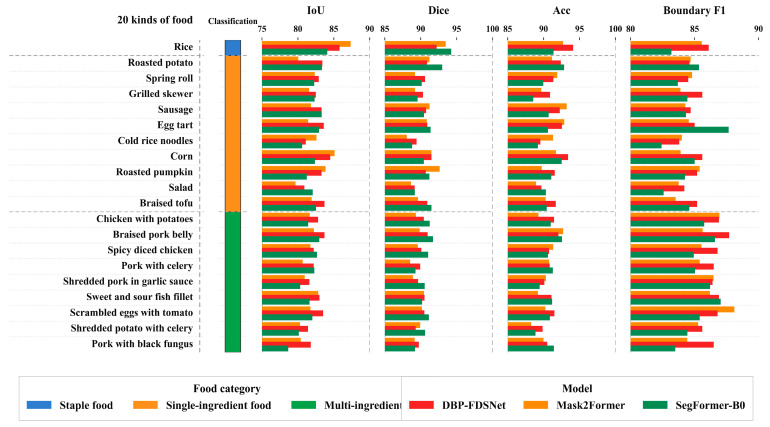
Performance comparison of the three best-performing models across 20 food categories.

Figure 11 presents the prediction results of different semantic segmentation models on four representative test samples. Figure 12 shows the distributions of Acc, IoU, and bF1 for the segmented regions of the test set based on three major food categories, using 100 randomly selected test samples. Figure 13 further compares the performance of the three best-performing models, namely DBP-FDSNet, Mask2Former, and SegFormer-B0, across 20 food categories, with Acc, IoU, and bF1 used as evaluation metrics. See file 1 in the supplementary document for specific images.

It can be observed that, for both staple foods and multi-ingredient dishes, most samples are distributed near or above the average line, suggesting that the model maintains good generalization ability across foods with different morphological characteristics. Table 3 reports the performance of each model on the four evaluation metrics.

In summary, the experimental results in this section show that DBP-FDSNet achieves better performance than the original FDSNet and other widely used segmentation models in the food semantic segmentation task. Its improved segmentation accuracy and boundary quality provide more reliable semantic masks for subsequent 3D volume estimation.

### 4.2. Parameter Selection for Multi-View 3D Reconstruction and Comparative Experiments on Semantic Mapping Strategies

This subsection presents systematic experiments and parameter analyses for multi-view 3D reconstruction and semantic mapping in bowl-served food scenarios. TSDF reconstruction accuracy is evaluated under different voxel resolutions and truncation distance settings to determine suitable reconstruction parameters. Four semantic mapping strategies are then compared, including Direct Projection, Bowl-Constrained Projection, Height-Field Projection, and Height-Field + Refinement, with their performance differences assessed using metrics such as back-projection mIoU and bF1. The results show that mapping consistency improves progressively with the introduction of bowl-interior geometric constraints, height-field representation, and local correction. A horizontal comparative analysis of the key parameters is further conducted to examine their influence on reconstruction and semantic mapping performance.

The experiments were conducted using 1452 groups of RGB-D image sequences paired with top-view RGB images, from which 145 groups were randomly selected for performance evaluation and parameter selection. The 3D surface of the food inside the bowl was reconstructed using RGB-TSDF voxel fusion. The main parameters included voxel resolution ranging from 1 mm to 3 mm, truncation distances of 2V, 4V, and 8V, where V denotes the voxel side length, and input-frame ratios of 60%, 80%, and 100%. Reconstruction quality was evaluated using Reprojection Depth RMSE, MAE, Hole Ratio, *Number of Small Connected Components* (<1%), overall volume MAPE, Runtime, and PeakMemory.

During semantic mapping, the trained DBP-FDSNet model is used to generate 2D semantic segmentation results from the top-view RGB images, and the resulting category labels are then mapped onto the reconstructed 3D food surface. To assess the consistency between the 2D semantic results and the 3D surface after mapping, back-projection mIoU, mDice, mAcc, and F1-Score are used as the main evaluation metrics.

Among these metrics, MAE, overall volume MAPE, Runtime, Peak Memory, back-projection mIoU, mDice, mAcc, and F1-Score follow the general definitions used in existing studies [49,50,51]. These metrics are used to evaluate depth or volume errors, computational efficiency, GPU memory consumption, and segmentation consistency after semantic mapping. Reprojection Depth RMSE is defined as the root mean square error between the depth of the reconstructed 3D surface after reprojection to the corresponding viewpoint and the original depth map within the valid region, reflecting the geometric consistency between the reconstructed surface and real depth observations. Hole Ratio denotes the proportion of holes in the reconstructed surface within the valid bowl region and is used to assess surface completeness. The Number of Small Connected Components refers to isolated connected components whose area or point-count proportion is less than 1%, which reflects the presence of floating fragments, pseudo-surfaces, and local noisy structures in the reconstruction result.

**Figure 14:**
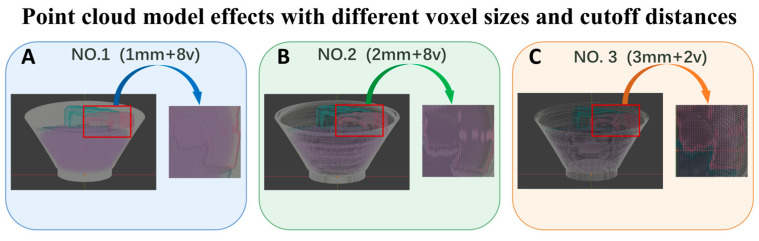
Comparison of 3D reconstruction accuracy under different RGB-TSDF parameter configurations. (**A**) corresponds to the NO.1 parameter combination, with a voxel size of 1 mm and a truncation distance of 8V. (**B**) corresponds to the NO.2 parameter combination, with a voxel size of 2 mm and a truncation distance of 8V. (**C**) corresponds to the NO.3 parameter combination, with a voxel size of 3 mm and a truncation distance of 2V.

**Figure 15:**
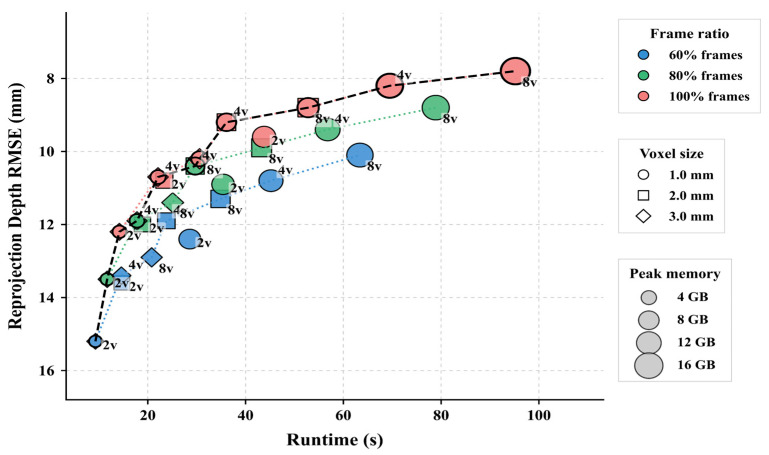
The relationship among *input-frame ratio*, *voxel size*, *truncation distance*, *peak memory usage*, Runtime, and *Reprojection Depth RMSE*.

**Figure 16:**
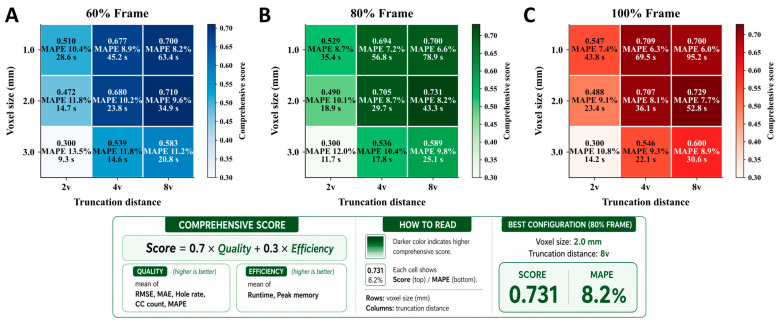
Present the overall score distributions under different voxel-size and truncation-distance combinations at input-frame ratios of 60%, 80%, and 100% (Separate picture of (**A**–**C**) triplets). Each cell lists the *overall score*, *overall volume MAPE*, and *Runtime* for the corresponding parameter setting. The bottom region of the figure summarizes the scoring mechanism and the final parameter selection results.

The 3D reconstruction accuracy was evaluated under different RGB-TSDF parameter settings. Figure 14 shows the reconstruction results obtained with different combinations of voxel size and truncation distance at input-frame ratios of 60%, 80%, and 100%. No. 1, with a parameter setting of 1 mm + 8 V, preserves local surface details more effectively, but the increased number of voxels also results in higher computational cost and GPU memory consumption. No. 2, using 2 mm + 8 V, provides a more balanced performance in terms of food-surface continuity, local detail preservation, and computational efficiency, and was therefore considered more suitable for the subsequent experiments. In contrast, No. 3, with a larger voxel size and a smaller truncation distance of 3 mm + 2 V, reduces the computational burden but makes the reconstructed surface more susceptible to holes, fractures, and granular artifacts. Overall, smaller voxel sizes and larger truncation distances tend to improve reconstruction accuracy, although this improvement comes at the cost of substantially increased computation and memory usage. Figure 15 shows the relationship among *input-frame ratio, voxel size, truncation distance, peak memory usage*, Runtime, and *Reprojection Depth RMSE*. Figure 16 presents the overall score distributions under different combinations of voxel size and truncation distance when the input-frame ratios are set to 60%, 80%, and 100%, corresponding to subfigures A, B, and C, respectively.

After the optimal RGB-TSDF reconstruction parameters are determined, the mapping from 2D semantic segmentation results to the 3D food surface is further analyzed. Since the top-view semantic masks need to be aligned with the reconstructed 3D surface in a unified bowl coordinate system, Direct Projection is easily disturbed by pseudo-surfaces outside the bowl, local holes, and uneven point-cloud sampling. To address these issues, four semantic mapping strategies are compared with progressive constraints and refinements: Direct Projection, Bowl-Constrained Projection, Height-Field Projection (including Bowl-Constrained Projection), and Height-Field + Refinement (including Bowl-Constrained Projection).

Among these strategies, Direct Projection assigns 2D semantic labels to the corresponding 3D surface points according to the imaging relationship of the top-view camera. Let p denote a 3D surface point, $\pi \left(\cdot \right)$ denote the projection function from 3D space to the top-view image plane, and $L_{2D}$ and $L_{3D}$ denote the 2D and 3D semantic labels, respectively. The mapping can be written as $L_{3D}\left(p\right)=L_{2D}\left(\pi \left(p\right)\right)$. Although this method is straightforward to implement, labels may be incorrectly assigned to noisy points outside the bowl or to reconstructed pseudo-surfaces.

Bowl-Constrained Projection builds on Direct Projection by introducing an inner-bowl constraint, so that only the valid 3D surface within the bowl model is retained. This helps reduce interference from background regions and abnormal points outside the bowl. To further improve the alignment between 2D semantics and 3D geometry, Height-Field Projection represents the 3D surface inside the bowl as a height field on the bowl-rim plane, expressed as $p\left(x,y\right)=\left(x,y,H\left(x,y\right)\right)$, where $H\left(x,y\right)$ denotes the visible surface height at the corresponding planar position. With this representation, the 2D semantic mask and the 3D surface share the same parameter domain, which reduces mapping loss caused by point-cloud sparsity and local holes.

The Height-Field Projection + Refinement strategy further applies local consistency correction to the height-field mapping results. By removing isolated small regions and smoothing category boundaries, this strategy reduces local boundary artifacts and improves the spatial coherence of the mapped semantic regions.

**Figure 17:**
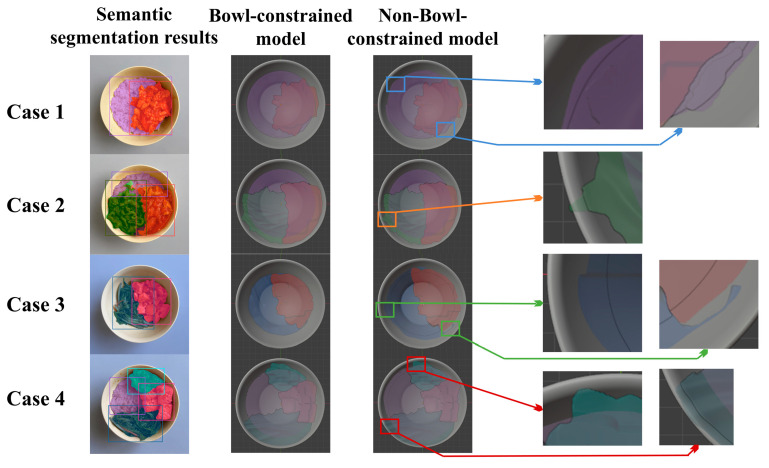
Comparison of the 3D semantic mapping results in four representative cases before and after the introduction of bowl constraints, with local enlarged views shown on the right.

**Figure 18:**
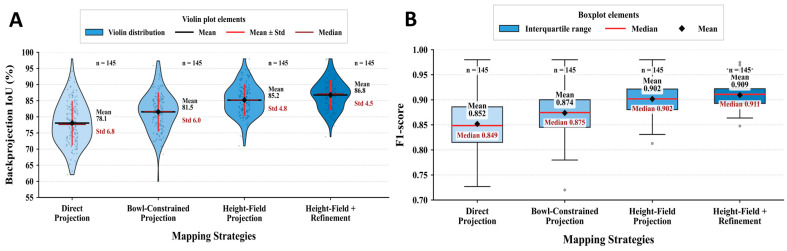
Back-projection IoU (**A**) and F1-score (**B**) results of the four strategies, illustrating their overall distribution, stability, and segmentation consistency across 145 samples.

**Figure 19:**
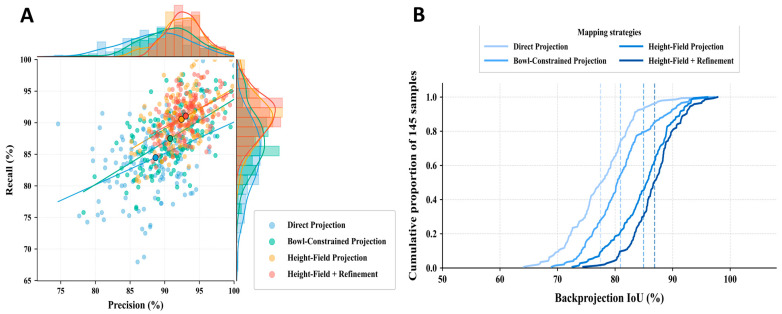
(**A**) the joint distribution of Precision and Recall, (**B**) the ECDF curves of back-projection IoU.

The role of the bowl instance model in semantic mapping was further examined through comparative experiments with and without bowl constraints. The results show that introducing bowl constraints markedly reduces the incorrect assignment of semantic labels to pseudo-surfaces outside the bowl, background points, and floating fragments. After the bowl cavity is cropped, invalid regions are removed, and the semantic labels become more concentrated on the valid food surface. This suggests that the bowl instance model not only provides the geometric boundary for subsequent volume integration, but also offers useful spatial guidance during semantic mapping. Table 4 presents the comparison results of different semantic mapping strategies. Figure 17 compares the three-dimensional semantic mapping results before and after the introduction of bowl constraints, with the enlarged local views shown on the right. Figure 18 reports the back-projection IoU and F1-score results of the four strategies. Figure 19 shows the *ECDF* curves of back-projection IoU, together with the joint distribution of Precision and Recall, illustrating the balance between mapping accuracy and completeness under different strategies.

With the gradual introduction of bowl constraints, height-field parameterization, and local refinement, the back-projection mIoU, F1-score, Precision, and Recall all improve consistently. These results demonstrate that the proposed height-field mapping and local refinement strategy can effectively enhance the spatial consistency between 2D semantic labels and the 3D food surface.

### 4.3. Experiments on Layered Volume Estimation Under Staple-Food Occlusion and Invisibility

The previous methods section described the staple-food occlusion compensation and invisibility handling strategies. This subsection further examines how the layered strategy influences volume estimation and weight conversion under different staple-food states. Based on the visibility of the staple-food region in the top-view semantic segmentation results, the samples are grouped into three cases for experimental analysis: partial occlusion, severe occlusion, and invisibility of staple food.

In the experimental statistics, 943 of the 1452 sample groups contained staple food. Among these samples, 74.3% were classified as severe occlusion cases, while the remaining samples belonged to the partial occlusion case. Another 503 groups were identified as staple-food invisibility or no-staple-food samples, among which invisible-staple-food samples accounted for 83.2% and no-staple-food samples accounted for 16.8%. The remaining samples were excluded because of data loss or unusable data. For samples containing staple food, the weight prediction MAE and MAPE were calculated only for the staple-food category, without including the errors of single-ingredient and multi-ingredient foods. For samples without staple food, the MAE and MAPE were calculated for single-ingredient and multi-ingredient foods.

This subsection mainly examines whether the layered processing strategy is necessary under staple-food occlusion and invisibility conditions. Since staple-food occlusion compensation directly determines the lower-boundary setting in the height field and further affects the volume integration of single-ingredient and multi-ingredient foods, the samples are grouped and analyzed according to different staple-food states before the volume estimation results are evaluated.

**Figure 20:**
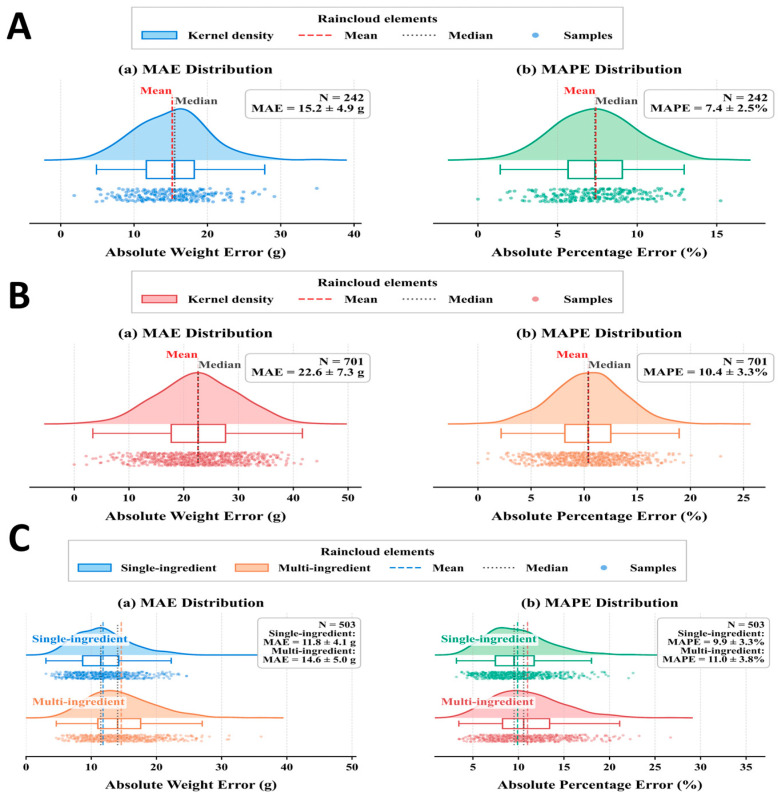
Error distributions of layered volume estimation and occlusion effects under different staple-food states. (**A**) Weight error distribution for samples with partially occluded staple foods, where the half-violin plot represents the error density, the box plot shows the median and interquartile range, and the scatter points indicate sample-level errors. (**B**) Weight error distribution for samples with severely occluded staple foods. (**C**) Weight error distributions of single-ingredient and multi-ingredient foods in samples without staple foods. In panel C, the category-specific dashed lines follow the corresponding food-category colors, with the single-ingredient and multi-ingredient foods shown by their respective colored mean lines in the *MAE* and *MAPE* distributions.

**Figure 21:**
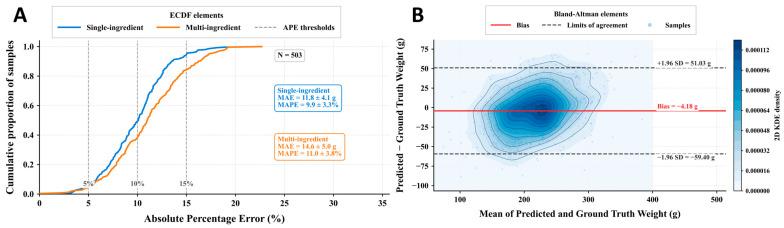
(**A**) ECDF curves of MAPE for single-ingredient and multi-ingredient foods in the no-staple-food scenario. (**B**) Density distributions of predicted and ground-truth weights under severe staple-food occlusion.

It should be noted that the weight-error statistics reported here do not represent a full analysis of the complete weight prediction pipeline. Rather, they are obtained under the subsequent unified 2D-to-3D semantic mapping, category-level volume segmentation settings, and volume-to-density conversion process. In the following subsection, parameter differentiation experiments are further conducted for single-ingredient and multi-ingredient food volume segmentation, focusing on the effects of smoothing strength, connected-component merging threshold, and boundary correction radius on weight MAPE. After the final parameters are determined, the overall weight and nutritional composition prediction results are analyzed. Table 5 presents the statistical results of food weight estimation under three different scenarios, using MAE and MAPE as evaluation metrics. Figure 20 shows the error distributions of layered volume estimation under different staple food states and occlusion conditions, including the weight error distribution for partially occluded staple food samples, that for severely occluded staple food samples, and the weight error distributions of single-component and multi-component foods in samples without staple foods. Figure 21 further presents the MAPE-based *ECDF* curves for single-component and multi-component foods under the no-staple-food condition, as well as the density distributions of predicted and ground-truth weights under severe staple food occlusion.

### 4.4. Food Mass Estimation and Whole-Bowl Nutritional Composition Prediction

After multi-view RGB-TSDF reconstruction, reconstruction strategy analysis, and layered processing for staple-food occlusion have been completed, the top-view semantic segmentation results are further mapped onto the 3D height-field surface in this subsection. Based on the resulting category-level volumes, food weight and nutritional composition are then predicted. It should be noted that the weight prediction in this section is not derived directly from the 2D semantic segmentation results alone; rather, it depends on the combined effects of the selected 3D reconstruction parameters and strategies, staple-food occlusion compensation, and height-field-based semantic mapping.

In the basic workflow, DBP-FDSNet is used to generate 2D semantic labels from the top-view image, after which these labels are mapped onto the height-field surface in the bowl coordinate system to determine the occupied 3D regions of staple foods, single-ingredient foods, and multi-ingredient foods. The category-level volume is then obtained by integrating the height difference between the upper and lower boundaries of each corresponding food region. Based on the density table of each food category, the estimated volume is converted into weight, and the four nutritional indicators of the whole bowl, including Calories, Fat, Carbohydrate, and Protein, are further calculated using the nutrient composition table per unit mass.

Before category-level volume segmentation, differentiated parameter settings are evaluated for single-ingredient and multi-ingredient foods. Single-ingredient foods generally have more continuous geometric shapes and relatively stable boundaries, whereas multi-ingredient foods contain more complex internal components and tend to show fragmented or mixed boundaries. For this reason, applying the same post-processing parameters to both categories may not be appropriate. In this study, 145 samples are used to examine how smoothing strength, connected-component merging threshold, and boundary correction radius affect weight MAPE. As shown in Table 6, the best performance for single-ingredient foods is obtained with a smoothing strength of 0.25, a connected-component merging threshold of 50, and a boundary correction radius of 2, while multi-ingredient foods perform best with a smoothing strength of 0.15, a connected-component merging threshold of 20, and a boundary correction radius of 1.

These results suggest that single-ingredient foods benefit from stronger smoothing and connected-component merging, as these operations help suppress local fragments. Multi-ingredient foods, however, require more local boundary details to be preserved, since excessive merging may blur category regions and introduce segmentation confusion. Therefore, the two optimized parameter sets are used in all subsequent experiments, while the default parameters are retained for staple foods.

**Figure 22:**
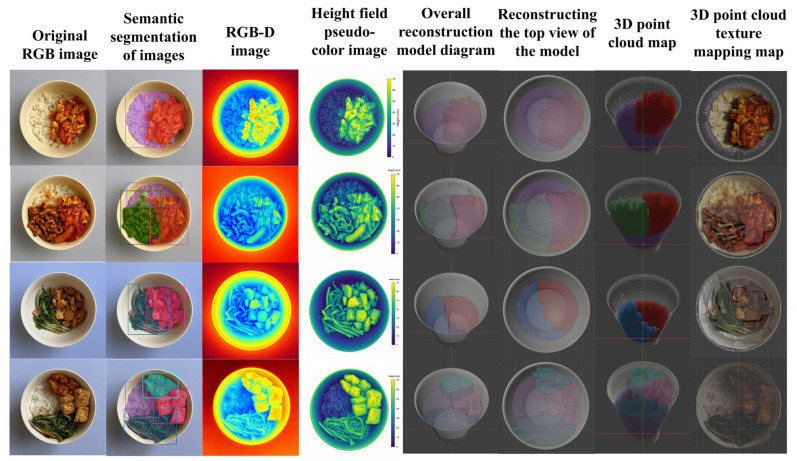
Representative results of 3D reconstruction and height-field analysis. The four representative cases, each including the original RGB image, semantic segmentation result, RGB-D depth image, pseudo-color height-field map, overall reconstructed model, top-view reconstructed model, 3D point cloud, and texture-mapped 3D point cloud. The Height field pseudo-color image in Figure 22 corresponds to the Height field pseudo-color image in Figure 23, where more details can be seen.

**Figure 23:**
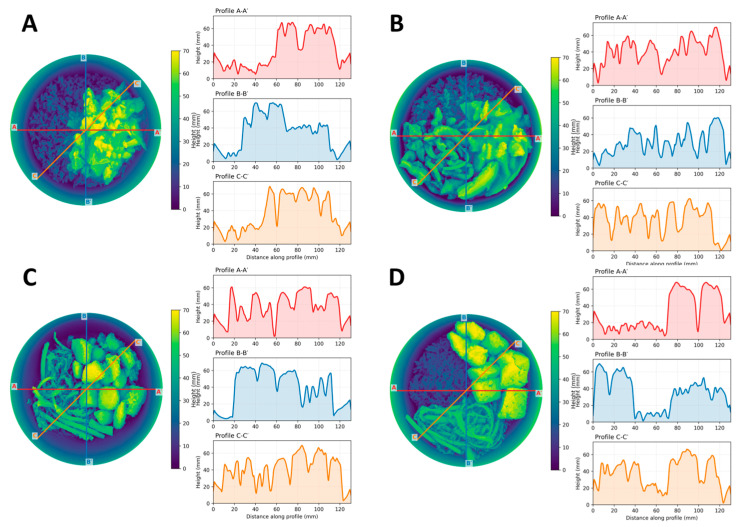
Local height-profile analysis results extracted from the height-field pseudo-color images corresponding to the four bowl-served food examples in Figure 22. (**A**–**D**) Height-field pseudo-color images and local height profiles of the first to fourth examples in Figure 22, respectively. In each subfigure, the left panel shows the reconstructed height distribution of the food surface, while the three curves on the right represent height variations along the marked A–A′, B–B′, and C–C′ directions.

**Figure 24:**
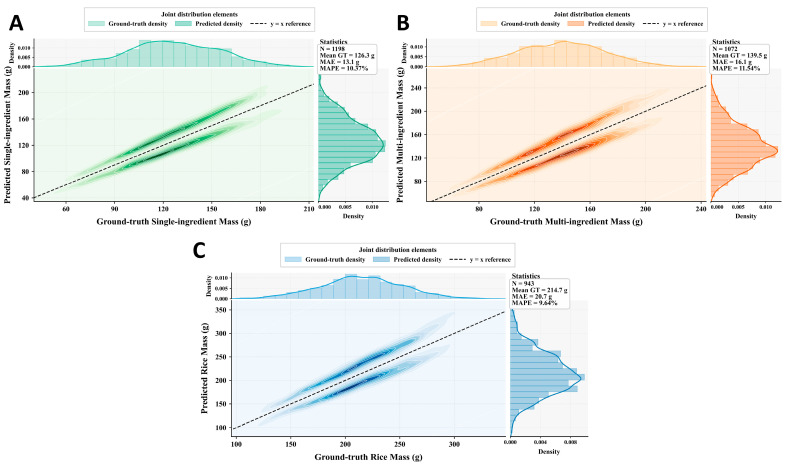
(**A**–**C**) show the relationships between ground-truth and predicted mass for single-ingredient foods, multi-ingredient foods, staple foods respectively, together with the corresponding error levels in terms of MAE and MAPE.

**Figure 25:**
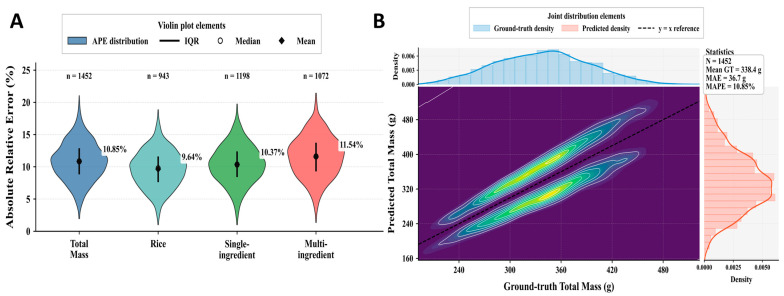
(**A**) compares the absolute and relative error distributions for four estimation tasks, namely total mass, staple foods, single-ingredient foods, and multi-ingredient foods, with their mean and median values also indicated. (**B**) presents the distribution of ground-truth and predicted values for total mass estimation.

Instead of directly assigning semantic labels to sparse point clouds, the 2D semantic results are mapped onto the 3D height-field model. In this representation, the reconstructed food surface inside the bowl is described in the top-view planar parameter domain, so that the semantic masks, bowl-model boundaries, and reconstructed surfaces can be aligned within a unified spatial framework. Figure 22 presents four representative cases, including the corresponding depth images, point clouds, 3D models, pseudo-color height-field maps, and semantic segmentation results. Figure 23 presents the local height-profile analysis results extracted from the corresponding pseudo-color height-field maps. After category-level volume segmentation was completed for the 1452 sample groups, the predicted mass results were further analyzed at both the whole-bowl level and the three-category level. Figure 24 and Figure 25 show the consistency and error distributions of mass estimation results across different food categories, with the detailed quantitative results reported in Table 7. The total mass of the whole bowl achieved an MAE of 36.7±8.9 g and a MAPE of 10.85±3.14%. Among the three major food categories, staple foods had the highest average ground-truth mass, reaching 214.7 g, with a MAPE of 9.64±3.02%. The MAPE values for single-ingredient and multi-ingredient foods were 10.37±3.41% and 11.54±3.87%, respectively. See File S2 and File S3 in the Supplementary Document for specific images.

**Figure 26:**
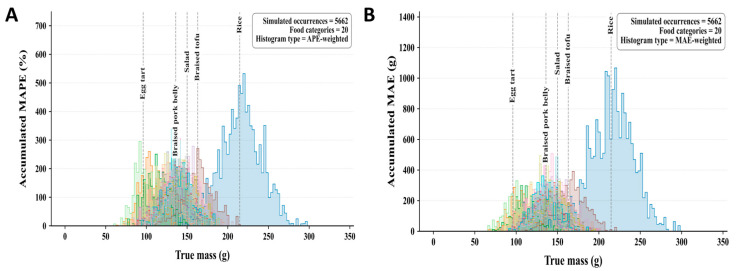
(**A**) Cumulative distributions of MAPE across different ground-truth mass intervals, (**B**) Cumulative distributions of MAE across different ground-truth mass intervals. The different colors represent the 20 food categories, and the color assignment is consistent with the category colors used in Figure 27, so that the contribution of each food category can be compared across figures. The vertical dashed lines indicate representative food-category mass positions, which are used to show where the main error accumulation occurs along the true-mass axis.

**Figure 27:**
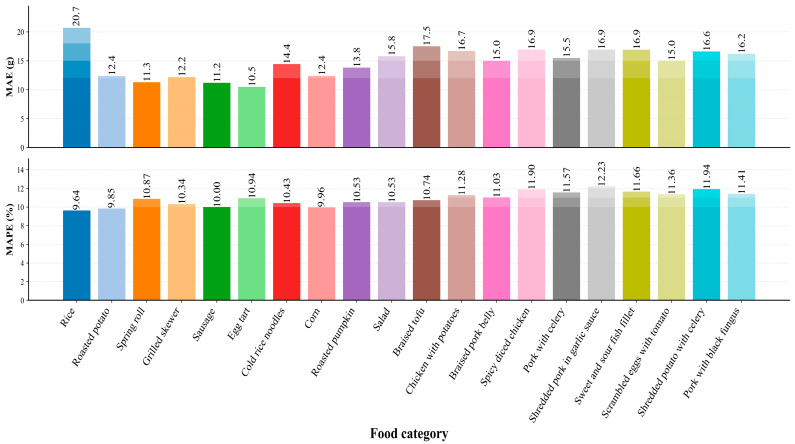
MAE and MAPE results for the 20 food categories under whole-bowl statistics.

We further calculated the average ground-truth mass, MAE, and MAPE for each of the 20 food categories, as shown in Figure 26 and Figure 27. The statistics show that 943 of the 1452 sample groups contained staple food. Apart from staple food, 75% of the bowls contained three food types, while the remaining 25% contained four food types; therefore, the total occurrence count of non-staple food categories was higher than the total number of samples. Overall, most single-ingredient foods showed MAPE values close to 10%, whereas multi-ingredient foods generally produced slightly higher errors, suggesting that more complex category structures can reduce the stability of 3D segmentation.

For nutritional composition prediction, the predicted weight of each food category is combined with the nutrient composition table per unit mass to calculate the whole-bowl Calories, Fat, Carbohydrate, and Protein. When the evaluation object is a specific food category rather than the total nutritional composition of the whole bowl, the relative error of nutritional prediction is the same as that of weight prediction, since a fixed nutrient composition value per unit mass is used for conversion. For the c-th food category, the predicted value of the k-th nutrient can be expressed as ${\hat{N}}_{c,k}=\frac{{\hat{m}}_{c}}{100}a_{c,k}$, while the ground-truth value is $N_{c,k}=\frac{m_{c}}{100}a_{c,k}$, where ${\hat{m}}_{c}$ and $m_{c}$ denote the predicted and ground-truth weights of this food category, respectively, and $a_{c,k}$ denotes the content of the k-th nutrient per 100 g of this category.

Since $a_{c,k}$ is a fixed constant for the same food category, when statistics are performed for a single food category, the MAPE values of the four nutritional components are the same as the weight MAPE of that category. The MAE values of different nutrients differ due to differences in nutrient content per unit mass. Only when the total nutritional composition of the whole bowl is evaluated do the overall errors of different nutrients need to be calculated separately, because different food categories have different nutrient densities, and the errors of different categories may be weighted, accumulated, or partially offset.

**Figure 28:**
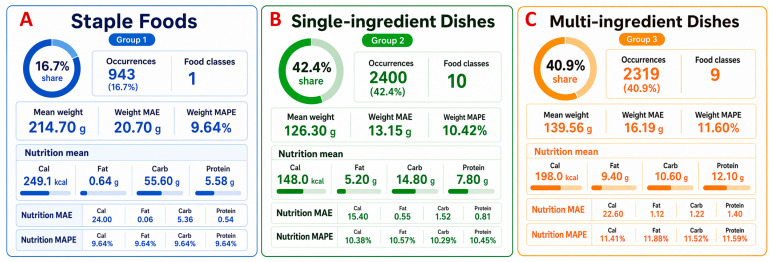
The sample proportions, occurrence frequencies, average mass, and prediction errors of the four nutritional components for staple foods (**A**), single-ingredient foods (**B**), and multi-ingredient foods (**C**), respectively.

Figure 28 shows the distribution proportions of the three major food categories and the prediction indicators for each nutrient and weight, illustrating the contribution of different food categories to the whole-bowl weight and nutritional indicators. Table 8 presents the total nutritional composition results for 1452 bowls. The results show that the MAPE values for whole-bowl Calories, Fat, Carbohydrate, and Protein are 13.23%, 18.51%, 14.18%, and 13.35%, respectively.

**Figure 29:**
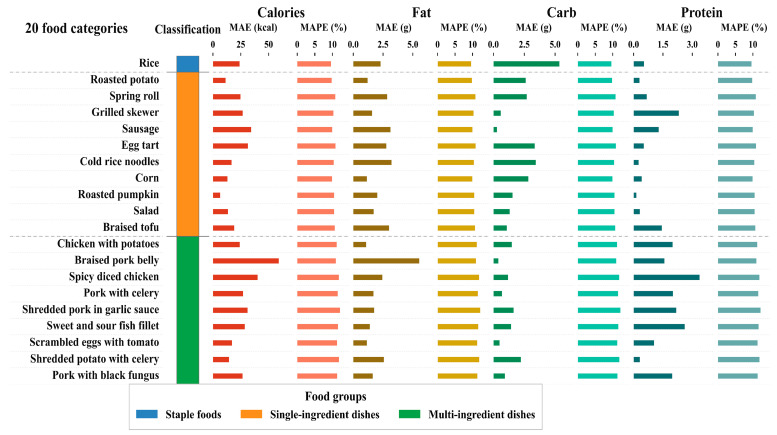
MAE and MAPE of the four nutritional components for 20 food categories across all samples, evaluated at the whole-bowl level.

To analyze the influence of different categories on nutritional prediction errors, we further calculated the MAE and MAPE of the three major food categories—staple foods, single-ingredient foods, and multi-ingredient foods—as well as all 20 food categories in total, for the four nutritional components, as shown in Figure 29. The results indicate that the MAPE values of the four nutritional indicators are 9.64% for staple foods, approximately 10.3–10.6% for single-ingredient foods, and approximately 11.4–11.9% for multi-ingredient foods, which is consistent with the previously observed trend in weight estimation errors. This is because, in the proposed method, the nutritional composition of the same food category is obtained by multiplying the predicted weight by a fixed unit nutrient content. Therefore, the category-level relative nutritional error is consistent with the category-level relative weight error.

To further compare the experimental results of the proposed method with those of simpler image-based or RGB-D fusion-based approaches, Table 9 presents the MAPE values for mass and nutritional composition prediction across several methods.

Table 9 provides a literature-level comparison rather than a strictly controlled direct evaluation. The compared methods were developed using different datasets, data-cleaning procedures, and evaluation settings, and some studies reported MAPE or percentage MAE rather than the MAPE metric used in the present study. Single-image methods generally have lower acquisition costs and are easier to deploy, but their prediction errors tend to depend more heavily on learned image features and estimated portion sizes. By contrast, multimodal and RGB-D fusion-based methods introduce additional depth, geometric, or ingredient-related information, which can improve the stability of mass and nutritional composition prediction.

In summary, this subsection determines the category-level volume segmentation parameters, analyzes weight prediction for the three major food groups and 20 food categories, and evaluates nutritional composition prediction at both the whole-bowl and category levels based on the mapping from 2D semantics to 3D height fields. The results show that differentiated mapping parameters better accommodate the morphological differences between single-ingredient and multi-ingredient foods, while the height-field-based semantic mapping method provides a stable correspondence between 2D labels and 3D surfaces. The whole-bowl total mass prediction achieves a MAPE of 10.85%. For nutritional composition prediction, the average MAPE of the four nutritional components is approximately 14.82%, with values of 13.23% for Calories, 18.51% for Fat, 14.18% for Carbohydrate, and 13.35% for Protein. It should be noted that the MAPE for Fat is higher than those of the other three nutritional indicators, which is mainly related to the conversion procedure adopted in this study, where volume is first converted into mass and then into nutritional composition. In other words, fat distribution is not directly identified from the images. Instead, food mass is first derived from category-level volume estimation results, after which nutrient contents are calculated according to a fixed nutrient composition table per unit mass. The relatively higher error for fat mainly results from the propagation of category-level mass estimation errors during the nutrient-coefficient weighting process. Compared with Calories and Carbohydrate, the average ground-truth value of whole-bowl Fat is smaller, which means that an absolute error of a similar magnitude will appear as a larger relative error in terms of MAPE. In addition, the fat content per unit mass varies substantially across different food categories, and when the volume or mass of high-fat categories is estimated with some bias, the resulting error is more likely to have a noticeable effect on the whole-bowl Fat prediction. By contrast, Carbohydrate is more strongly influenced by the mass of staple foods such as rice, for which a relatively stable volume estimation basis is provided after occlusion compensation of the staple layer. The prediction error for Protein is also mainly affected by the mass estimation of the corresponding food categories, and therefore its overall MAPE remains lower than that of Fat.

The average MAPE across mass and the four nutritional components is 14.02%. These results suggest that the proposed pipeline, which links 2D semantic segmentation, 3D height-field-based category-level volume estimation, and volume-to-nutrition conversion, is feasible for nutritional analysis of multi-category bowl-served foods.

## 5. Limitations and Discussion of This Study

Although a relatively complete pipeline for predicting the nutritional composition of multi-category bowl-based foods was established in this study, several limitations remain. The current data acquisition process still relies on a top-mounted RGB camera and a surrounding RGB-D camera, while the equipment structure is relatively fixed. Therefore, accurate camera calibration, high-quality depth maps, and stable imaging conditions are required, which limit the flexibility of practical application. In addition, when staple foods are completely invisible, the system still relies on the staple-food weight provided by the user as auxiliary information, indicating that the degree of automation still needs to be further improved. Nutritional composition prediction also depends on food density values and nutritional databases, which makes it difficult to fully account for differences in cooking methods, moisture content, and specific recipes. These issues constitute the main limitations of the proposed method, and Table 10 summarizes these limitations according to different stages.

## 6. Conclusions

This study proposed a complete pipeline for volume estimation and nutritional composition prediction of multi-category bowl-based meals, covering the entire process from image acquisition and semantic segmentation to three-dimensional reconstruction and nutritional composition prediction.

For food semantic segmentation, DBP-FDSNet was developed on the basis of the original model to adapt it to boundary adhesion and texture similarity, which are commonly observed in top-view images of bowl-based foods. Targeted improvements were made to the original network by introducing a detail-enhanced shallow branch, a boundary-assisted supervision branch, and spatial position encoding. DBP-FDSNet achieved an mIoU of 80.51%, a bF1 of 85.73%, and an mAcc of 90.06%. The three-dimensional food surface inside the bowl was reconstructed through RGB-TSDF fusion, with the selected reconstruction parameters set as a voxel resolution of *2* mm, a truncation distance of *4* V, and an image input ratio of 100%. Four strategies were also compared, and Height-Field + Refinement achieved the best mapping performance, with a back-projection mIoU of 86.9%, an mDice of 92.8%, an mAcc of 93.7%, and a bF1 of 91.9%.

In terms of volume estimation, this study focuses on the problem that staple foods in bowl-based meals are often occluded by upper-layer foods. According to the visible proportion of staple foods, the samples were divided into three cases: partial occlusion, severe occlusion, and complete invisibility. The experimental results show that, among 1452 sample groups, the MAE and MAPE of staple-food weight prediction for samples with partially occluded staple foods were 15.2 g and 7.4%, respectively. For samples in which staple foods were severely occluded, the corresponding MAE and MAPE were 22.6 g and 10.4%, respectively. The MAPE of total food mass prediction at the whole-bowl level was 10.85%. However, when staple foods are completely invisible, the current workflow remains semi-automatic, which may limit its application in fully unattended daily dietary assessment scenarios.

In terms of nutritional composition prediction, the category-level volume estimates were first converted into mass according to food density, and the contents of whole-bowl Carbohydrate, Fat, and Protein were then calculated using a nutritional composition table per unit mass. The experimental results show that, across 1452 bowl-based meal samples, the prediction MAE for whole-bowl Calories was 95.6 kcal, with a MAPE of 13.23%. For Fat, the MAE and MAPE were 4.44 g and 18.51%, respectively. For Carbohydrate, the MAE was 10.99 g, and the MAPE was 14.18%. For Protein, the MAE and MAPE were 4.78 g and 13.35%, respectively.

By integrating DBP-FDSNet-based semantic segmentation, RGB-TSDF multi-view reconstruction, bowl instance-based geometric constraints, staple-food occlusion compensation, and category-level height-field fusion, the proposed method can achieve relatively stable nutritional composition prediction for multi-category bowl-based meals. However, differences still remain between the current hardware system and real daily dining environments. Future work should further simplify the acquisition equipment and evaluate the practical applicability of this method under handheld imaging conditions and in open dining-table scenarios.

## Figures and Tables

**Table 1:** Training Configurations and Hyperparameter Settings of Different Semantic Segmentation Models.

Model	Datasets	Input	Epoch	Batch Size	Optimizer	LR	Sch *	Weight Decay	Warmup Epochs
DeepLabV3+ (R50 init)	UFP *	768 × 768	120	8	SGD	7 × 10^−3^	Poly	1 × 10^−4^	0
BiSeNetV2	UFP	768 × 768	120	8	SGD	1 × 10^−2^	Poly	5 × 10^−4^	5
SegFormer-B0 (MiT-B0 init)	UFP	768 × 768	120	8	AdamW	6 × 10^−5^	Poly	1 × 10^−2^	5
FDSNet (Swin branch)	ImageNet-22K	384 × 384	90	4096	AdamW	1 × 10^−3^	Cosine	5 × 10^−2^	5
FDSNet (STDC branch)	ImageNet-1K	512 × 1024	300	64	SGD	1 × 10^−2^	Cosine	1 × 10^−4^	5
FDSNet	UFP	768 × 768	120	8	AdamW	1 × 10^−5^	Poly	1 × 10^−2^	2
DBP-FDSNet (Swin branch)	ImageNet-22K	384 × 384	90	4096	AdamW	1 × 10^−3^	Cosine	5 × 10^−2^	5
DBP-FDSNet (STDC branch)	ImageNet-1K	512 × 1024	300	64	SGD	1 × 10^−2^	Cosine	1 × 10^−4^	5
DBP-FDSNet	UFP	768 × 768	120	8	AdamW	1 × 10^−5^	Poly	1 × 10^−2^	2

* UFP denotes the UECFoodPixComplete food semantic segmentation dataset used in this study for training and fine-tuning each model on the target task. The dataset contains top-view RGB images of multiple food categories together with their corresponding semantic segmentation annotations. Sch denotes the learning rate scheduling strategy; Poly refers to polynomial decay, where the learning rate gradually decreases as training iterations proceed, while Cosine refers to cosine annealing, where the learning rate decays following a cosine curve.

**Table 2:** Ablation Results With and Without UFP-Based Pretraining.

D	B	P	mIoU	mDice	mAcc	bF1	*FLOPs (G)*	*Params (M)*
✗	✗	✗	78.31 (+1.83)	87.42 (+1.41)	88.65 (+1.32)	80.26 (+2.05)	182.74	101.93
✓	✗	✗	78.42 (+2.66)	88.19 (+1.22)	89.14 (+1.21)	81.87 (+1.75)	189.12	104.18
✗	✓	✗	79.18 (+1.79)	87.96 (+1.26)	88.96 (+1.25)	82.43 (+1.62)	182.74	101.93
✗	✗	✓	77.64 (+2.22)	85.58 (+2.50)	86.41 (+3.13)	81.32 (+2.02)	183.31	102.02
✓	✓	✗	80.36 (+1.56)	88.78 (+1.09)	89.73 (+1.10)	83.54 (+1.29)	189.12	104.18
✓	✗	✓	79.09 (+1.64)	87.67 (+1.12)	88.95 (+1.01)	82.96 (+1.51)	189.69	104.27
✗	✓	✓	79.91 (+1.67)	88.45 (+1.20)	89.88 (+1.04)	83.21 (+1.48)	183.31	102.02
✓	✓	✓	81.27 (+1.59)	89.56 (+0.86)	90.37 (+1.01)	84.61 (+1.12)	189.69	104.27

‘✗’ indicates that the module is not used, and ‘✓’ indicates that the module is used. D denotes the detail-enhanced shallow-branch input, B denotes boundary-assisted supervision, and P denotes spatial encoding. The values in parentheses represent the performance gains achieved after transferring the model pretrained on the UFP dataset to the target task, relative to the corresponding settings without transfer learning.

**Table 3:** Comparative Performance of Different Semantic Segmentation Models on the Food Test Set.

Model	mIoU	mDice	mAcc	bF1 *
U-Net (trained from scratch) [44]	78.73	87.86	88.91	81.27
DeepLabV3+ (R50) * [41]	81.28	89.31	90.19	84.06
BiSeNetV2 [42]	80.41	88.94	89.86	83.33
SegFormer-B0 * [43]	81.96	90.01	90.74	84.82
QueryInst [45]	81.52	89.66	90.33	84.44
BoundaryFormer [46]	81.63	89.74	90.41	84.98
Mask2Former [47]	82.01	90.07	90.78	85.12
MaskDINO [48]	81.78	89.89	90.56	84.93
DBP-FDSNet(ours)	82.86	90.42	91.38	85.73

* R50 denotes the ResNet-50 backbone, meaning that the model uses ResNet-50 for feature extraction. B0 refers to the lightweight SegFormer variant equipped with the MiT-B0 encoder. Since MiT-B0 is the default encoder of SegFormer-B0, its backbone is not specified separately in the table. bF1 denotes the Boundary F1 score.

**Table 4:** Comparative Results of Different Semantic Mapping Strategies.

Mapping Strategies	Back-Projection $mIoU\left(\%\right)$	$F1-Score\left(\%\right)$	$Precision\left(\%\right)$	$Recall\left(\%\right)$
Direct Projection	77.8 ± 6.4	86.3 ± 5.1	88.6 ± 4.8	84.2 ± 5.7
Bowl-Constrained Projection	81.0 ± 5.6	89.1 ± 4.3	90.7 ± 4.0	87.6 ± 4.8
Height-Field Projection	84.9 ± 4.8	91.5 ± 3.6	92.6 ± 3.2	90.5 ± 4.0
Height-Field + Refinement	86.9 ± 4.2	92.8 ± 3.1	93.7 ± 2.8	91.9 ± 3.5

**Table 5:** Statistics of MAE and MAPE for Food Weight Under Three Scenarios.

Scenario Type	Number of Data Groups	Staple Food		Single-Ingredient Food		Multi-Ingredient Food	
		MAE(g)	MAPE(%)	MAE(g)	MAPE(%)	MAE(g)	MAPE(%)
Partial Occlusion of Staple Food	242	15.2 ± 5.1	7.4 ± 2.6	-	-	-	-
Severe Occlusion of Staple Food	701	22.6 ± 7.3	10.4 ± 3.4	-	-	-	-
Overall Samples Containing Staple Food	943	20.7 ± 6.8	9.64 ± 3.02	-	-	-	-
Invisible Staple Food/No Staple Food	503	-	-	11.8 ± 4.1	9.9 ± 3.3	14.6 ± 5.0	11.0 ± 3.8

**Table 6:** Results of Differentiated Parameter Settings for Single-Ingredient and Multi-Ingredient Foods.

Smoothing Strength	Connected-Component Merging Threshold	Boundary Correction Radius	Single-Ingredient Weight MAPE (%)	Multi-Ingredient Weight MAPE (%)
0.10	20	1	13.1	12.4
0.15	20	1	12.2	**11.6**
0.20	30	1	11.3	11.9
0.20	40	2	10.8	12.5
0.25	40	2	10.4	12.2
0.25	50	2	**10.1**	12.0
0.30	50	2	10.5	12.6
0.30	55	3	10.9	13.4
0.35	60	3	11.6	14.3

**Table 7:** Mass Estimation Errors for Different Statistical Categories Across 1452 Samples.

Statistical Object	Average Ground-Truth Mass (g)	MAE (g)	MAPE (%)
Total Mass	338.4	36.7 ± 8.9	10.85 ± 3.14
Staple Foods	214.7	20.7 ± 6.8	9.64 ± 3.02
Single-Ingredient Foods	126.3	13.1 ± 4.6	10.37 ± 3.41
Multi-Ingredient Foods	139.5	16.1 ± 5.4	11.54 ± 3.87

**Table 8:** Total Nutritional Composition Results of the Whole Bowl Across All Samples.

Nutritional Component	Average Ground-Truth Content	MAE (Kcal/g)	MAPE (%)
Calories (kcal)	722.4	95.6	13.23
Fat (g)	24.0	4.44	18.51
Carb (g)	77.5	10.99	14.18
Protein (g)	35.8	4.78	13.35

**Table 9:** Multi-method comparison of MAPE for food mass and nutritional composition prediction.

Method	Method Type	Calories	Fat	Carbohydrate	Protein	Mass
Google Nutrition 5K [52]	Single image	26.1	34.2	31.9	29.5	18.8
Swin-Nutrition [53]	Single image	15.3	22.1	20.8	15.4	12.5
U-Net + ResNet101 [10]	Single image	15.68	21.41	18.79	17.68	11.75
Google Nutrition 5K depth [52]	RGB-D fusion	18.8	18.1	23.8	20.9	18.9
RGB-D Nutrition [54]	RGB-D fusion	15.0	23.5	22.4	21.0	10.8
DPF-Nutrition [9]	RGB-D fusion	14.7	22.6	20.7	20.2	10.6
RDINet [55]	RGB-D fusion	14.9	19.7	18.9	19.5	11.2
Our Method	/	13.23	18.51	14.18	13.35	10.85

**Table 10:** Limitations of the Proposed Method Summarized by Stage.

Stage	Main Limitations	Possible Impact
Data Acquisition	The system requires coordinated operation between a top-view RGB camera and a surrounding RGB-D camera, while also relying on stable calibration relationships and a fixed acquisition trajectory.	This limits its direct application to ordinary mobile devices or open dining environments.
Dataset Scope	The dataset covers multiple categories of bowl-based meals, and its sample composition is closer to Chinese or East Asian mixed-meal scenarios. However, additional evaluations have not yet been conducted for broader dietary cultures or different container types.	This may limit the adaptability of the proposed method to other food category systems, non-bowl-based meals, and different food plating styles.
Semantic Segmentation	Mis-segmentation may still occur for foods with low contrast, similar colors, mixed textures, or adhesive boundaries.	These errors may shift the mapped 3D semantic regions and affect category-level volume allocation.
3D Reconstruction	Depth noise in RGB-D data, reflective surfaces, occluded regions, and camera pose errors may affect the quality of RGB-TSDF fusion.	The reconstructed model may contain local surface holes, boundary drift, or discontinuities in the height field.
Staple-Food Occlusion Compensation	When the staple food is completely invisible but actually present, the current method uses the user-provided staple food weight as auxiliary information to infer the height of the hidden staple layer. This fallback strategy is not required for partially or severely occluded cases, where automatic estimation can still be performed based on visible regions and geometric constraints; however, it also means that fully invisible staple foods cannot yet be handled in a completely automated manner.	This limits the degree of automation of the proposed pipeline under extreme occlusion conditions and may reduce its practicality in fully unattended dietary assessment scenarios.
Height-Field-Based Volume Segmentation	The height field uses the bowl-rim plane as the default parameter domain, making it more suitable for layered or approximately continuous food surfaces.	Its ability to represent suspended foods, soup-like mixtures, or complex stacking structures is limited.
Nutritional Composition Mapping	Food density and unit nutritional composition values are usually obtained from databases or measured averages.	Differences caused by cooking methods, moisture content, and individual recipes are difficult to fully capture.

## Data Availability

The original contributions presented in this study are included in the article/Supplementary Materials. Further inquiries can be directed to the corresponding authors.

## Supplementary Materials

The following supporting information can be downloaded at https://www.mdpi.com/article/10.3390/nu18132119/s1: File S1: Semantic segmentation graphs of different models, File S2: Height field pseudo-color image, File S3: RGB-D images.

## Abbreviations

The following abbreviations are used in this manuscript:

FAR	*Food area ratio*
UFP	UEC Food Pix Complete
MRF	Multi-scale Relationship-aware Feature fusion Module
TSDF	Truncated Signed Distance Function
RGB-TSDF	RGB-Truncated Signed Distance Function
PMAE	*Percentage Mean Absolute Error*
mIoU	*mean Intersection over Union*
bF1	*Boundary F1 Score*
mAcc	*mean Accuracy*
MAE	*Mean Absolute Error*
MAPE	*Mean Absolute Percentage Error*
mDice	*mean Dice Coefficient*

## References

[1] Zheng J., Wang J., Shen J., An R. (2024). Artificial intelligence applications to measure food and nutrient intakes: Scoping review. J. Med. Internet Res..

[2] Jayan H., Min W., Guo Z. (2025). Applications of artificial intelligence in food industry. Foods.

[3] Aghababaei A., Aghababaei F., Pignitter M., Hadidi M. (2025). Artificial intelligence in agro-food systems: From farm to fork. Foods.

[4] Muñoz B., Martínez-Arroyo A., Acevedo C., Aguilar E. (2025). Lightweight DeepLabv3+ for Semantic Food Segmentation. Foods.

[5] Yang Y., Jia W., Bucher T., Zhang H., Sun M. (2019). Image-based food portion size estimation using a smartphone without a fiducial marker. Public Health Nutr..

[6] Jia W., Li B., Xu Q., Chen G., Mao Z.-H., McCrory M.A., Baranowski T., Burke L.E., Lo B., Anderson A.K. (2024). Image-based volume estimation for food in a bowl. J. Food Eng..

[7] Jia W., Li B., Zheng Y., Mao Z.-H., Sun M. (2023). Estimating amount of food in a circular dining bowl from a single image. Proceedings of the 8th International Workshop on Multimedia Assisted Dietary Management.

[8] Lee K.-S. (2024). Multi-spectral food classification and caloric estimation using predicted images. Foods.

[9] Han Y., Cheng Q., Wu W., Huang Z. (2023). Dpf-nutrition: Food nutrition estimation via depth prediction and fusion. Foods.

[10] Zhao Y., Zhu P., Jiang Y., Xia K. (2024). Visual nutrition analysis: Leveraging segmentation and regression for food nutrient estimation. Front. Nutr..

[11] Feng Y., Wang Y., Wang X., Zhao B., Bi J., Han S., Xiao Z., Luo Y. (2025). RGB-D food nutrient estimation supported by FLAVA contrastive learning. J. Food Compos. Anal..

[12] Lo F.P.-W., Sun Y., Qiu J., Lo B. (2018). Food volume estimation based on deep learning view synthesis from a single depth map. Nutrients.

[13] Theera-Ampornpunt N., Treepong P. (2025). Visual Food Ingredient Prediction Using Deep Learning with Direct F-Score Optimization. Foods.

[14] Xiao Z., Li Y., Deng Z. (2025). Food image segmentation based on deep and shallow dual-branch network. Multimed. Syst..

[15] Okamoto K., Yanai K. UEC-FoodPIX Complete: A large-scale food image segmentation dataset. Proceedings of the International Conference on Pattern Recognition.

[16] Li Y., Huang S., Chen Y., Ding Y., Zhao P., Hu Q., Zhang X. (2024). RGBTSDF: An Efficient and Simple Method for Color Truncated Signed Distance Field (TSDF) Volume Fusion Based on RGB-D Images. Remote Sens..

[17] Wang W., Min W., Li T., Dong X., Li H., Jiang S. (2022). A review on vision-based analysis for automatic dietary assessment. Trends Food Sci. Technol..

[18] Zheng X., Liu C., Gong Y., Yin Q., Jia W., Sun M. (2023). Food volume estimation by multi-layer superpixel. Math. Biosci. Eng..

[19] Kwan Z., Zhang W., Wang Z., Ng A.B., See S. (2025). Nutrition estimation for dietary management: A transformer approach with depth sensing. IEEE Trans. Multimed..

[20] Baban A., Erep T.R., Chaari L. (2023). mid-deeplabv3+: A novel approach for image semantic segmentation applied to African food dietary assessments. Sensors.

[21] Lin C.-H., Huang J.-T. (2025). Nutritional assessment system integrating semantic segmentation and point cloud modeling techniques. Sci. Rep..

[22] Ghiasi G., Fowlkes C.C. Laplacian pyramid reconstruction and refinement for semantic segmentation. Proceedings of the European Conference on Computer Vision.

[23] Takikawa T., Acuna D., Jampani V., Fidler S. Gated-scnn: Gated shape cnns for semantic segmentation. Proceedings of the IEEE/CVF International Conference on Computer Vision.

[24] Zhou Q., Wang L., Gao G., Kang B., Ou W., Lu H. (2024). Boundary-guided lightweight semantic segmentation with multi-scale semantic context. IEEE Trans. Multimed..

[25] Yang Z., Cao Z., Cao J., Chen Z., Peng C. (2024). Multibranch semantic image segmentation model based on edge optimization and category perception. PLoS ONE.

[26] Liu R., Lehman J., Molino P., Petroski Such F., Frank E., Sergeev A., Yosinski J. (2018). An intriguing failing of convolutional neural networks and the coordconv solution. Adv. Neural Inf. Process. Syst..

[27] Fu X., Zhao S., Wang C., Tang X., Tao D., Li G., Jiao L., Dong D. (2024). Green fruit detection with a small dataset under a similar color background based on the improved YOLOv5-AT. Foods.

[28] Lin Q., Chen X., Chen C., Garibaldi J.M. (2024). Boundary-wise loss for medical image segmentation based on fuzzy rough sets. Inf. Sci..

[29] AlMughrabi A., Haroon U., Marques R., Radeva P. VolTex: Food volume estimation using text-guided segmentation and neural surface reconstruction. Proceedings of the Computer Vision and Pattern Recognition Conference.

[30] Ji X., Song K., Sun L., Lu H., Zhang H., Feng Y. (2026). A Method for Reconstructing and Predicting the Volume of Bowl-Type Tableware and Its Application in Dietary Analysis. Symmetry.

[31] Dehais J., Anthimopoulos M., Shevchik S., Mougiakakou S. (2016). Two-view 3D reconstruction for food volume estimation. IEEE Trans. Multimed..

[32] Weder S., Schonberger J., Pollefeys M., Oswald M.R. Routedfusion: Learning real-time depth map fusion. Proceedings of the IEEE/CVF Conference on Computer Vision and Pattern Recognition.

[33] Guo C., Zhang L., Shen Y., Zhou Y. Chunkfusion: A learning-based RGB-D 3D reconstruction framework via chunk-wise integration. Proceedings of the ICASSP 2022-2022 IEEE International Conference on Acoustics, Speech and Signal Processing (ICASSP).

[34] AlMughrabi A., Haroon U., Marques R., Radeva P. (2024). Voleta: One-and few-shot food volume estimation. arXiv.

[35] Gonzalez B., Garcia G., Velastin S.A., GholamHosseini H., Tejeda L., Farias G. (2024). Automated food weight and content estimation using computer vision and AI algorithms. Sensors.

[36] Tanabe H., Yanai K. Calorievol: Integrating volumetric context into multimodal large language models for image-based calorie estimation. Proceedings of the International Conference on Multimedia Modeling.

[37] Charrondiere U.R., Haytowitz D., Stadlmayr B. (2012). FAO/INFOODS density database, version 2.0. Proceedings of the Food and Agriculture Organization of the United Nations Technical Workshop Report.

[38] Fukagawa N.K., McKillop K., Pehrsson P.R., Moshfegh A., Harnly J., Finley J. (2022). USDA’s FoodData Central: What is it and why is it needed today?. Am. J. Clin. Nutr..

[39] Maier-Hein L., Reinke A., Godau P., Tizabi M.D., Buettner F., Christodoulou E., Glocker B., Isensee F., Kleesiek J., Kozubek M. (2024). Metrics reloaded: Recommendations for image analysis validation. Nat. Methods.

[40] Cheng B., Girshick R., Dollár P., Berg A.C., Kirillov A. Boundary IoU: Improving object-centric image segmentation evaluation. Proceedings of the IEEE/CVF Conference on Computer Vision and Pattern Recognition.

[41] Chen L.-C., Zhu Y., Papandreou G., Schroff F., Adam H. Encoder-decoder with atrous separable convolution for semantic image segmentation. Proceedings of the European Conference on Computer Vision (ECCV).

[42] Yu C., Gao C., Wang J., Yu G., Shen C., Sang N. (2021). Bisenet v2: Bilateral network with guided aggregation for real-time semantic segmentation. Int. J. Comput. Vis..

[43] Xie E., Wang W., Yu Z., Anandkumar A., Alvarez J.M., Luo P. (2021). SegFormer: Simple and efficient design for semantic segmentation with transformers. Adv. Neural Inf. Process. Syst..

[44] Ronneberger O., Fischer P., Brox T. U-net: Convolutional networks for biomedical image segmentation. Proceedings of the International Conference on Medical Image Computing and Computer-Assisted Intervention.

[45] Fang Y., Yang S., Wang X., Li Y., Fang C., Shan Y., Feng B., Liu W. Instances as queries. Proceedings of the IEEE/CVF International Conference on Computer Vision.

[46] Lazarow J., Xu W., Tu Z. Instance segmentation with mask-supervised polygonal boundary transformers. Proceedings of the IEEE/CVF Conference on Computer Vision and Pattern Recognition.

[47] Cheng B., Misra I., Schwing A.G., Kirillov A., Girdhar R. Masked-attention mask transformer for universal image segmentation. Proceedings of the IEEE/CVF Conference on Computer Vision and Pattern Recognition.

[48] Li F., Zhang H., Xu H., Liu S., Zhang L., Ni L.M., Shum H.-Y. Mask dino: Towards a unified transformer-based framework for object detection and segmentation. Proceedings of the IEEE/CVF Conference on Computer Vision and Pattern Recognition.

[49] Spencer J., Tosi F., Poggi M., Arora R.S., Russell C., Hadfield S., Bowden R., Zhou G., Li Z., Rao Q. The third monocular depth estimation challenge. Proceedings of the IEEE/CVF Conference on Computer Vision and Pattern Recognition.

[50] Chen Y., He J., Vinod G., Raghavan S., Czarnecki C., Ma J., Mahmud T.I., Coburn B., Mao D., Nair S. (2024). MetaFood3D: 3D food dataset with nutrition values. arXiv.

[51] Zhang Y., Mehta S., Caspi A. (2021). Rethinking semantic segmentation evaluation for explainability and model selection. arXiv.

[52] Thames Q., Karpur A., Norris W., Xia F., Panait L., Weyand T., Sim J. Nutrition5k: Towards automatic nutritional understanding of generic food. Proceedings of the IEEE/CVF Conference on Computer Vision and Pattern Recognition.

[53] Shao W., Hou S., Jia W., Zheng Y. (2022). Rapid non-destructive analysis of food nutrient content using swin-nutrition. Foods.

[54] Shao W., Min W., Hou S., Luo M., Li T., Zheng Y., Jiang S. (2023). Vision-based food nutrition estimation via RGB-D fusion network. Food Chem..

[55] Kuang Z., Gao H., Yu J., Sun D., Zhao J., Sun L. (2026). RDINet: A Deep Learning Model Integrating RGB-D and Ingredient Features for Food Nutrition Estimation. Appl. Sci..

