# Supplementary figures and images for: Image-Based Prediction of Food Weight and Nutritional Composition in Bowl-Served Meals Using Semantic Segmentation and Multi-View 3D Reconstruction
Source: Nutrients. 2026 Jun 30;18(13):2119. doi: 10.3390/nu18132119 (PMC13363716; doi:10.3390/nu18132119)

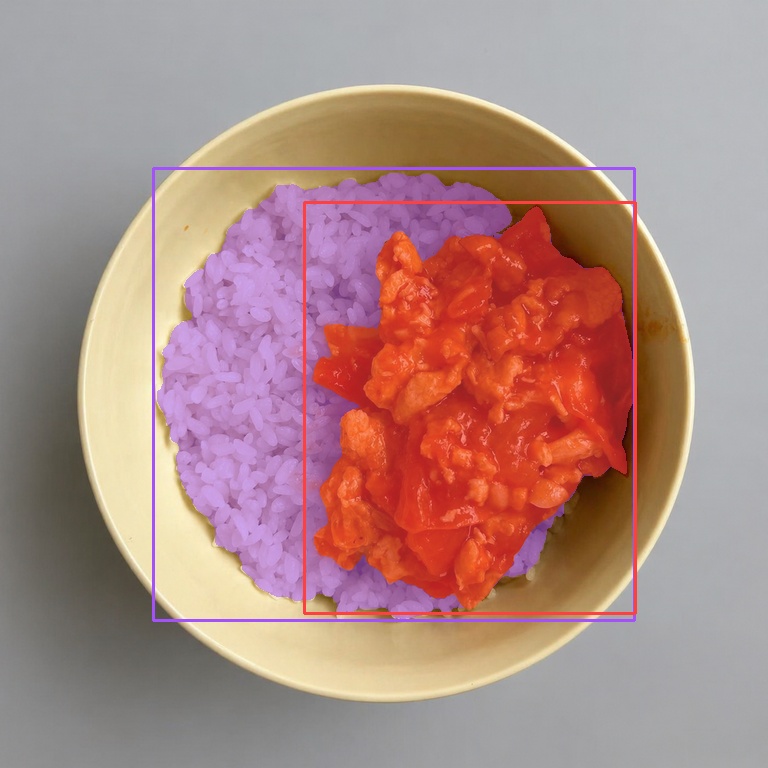

Supplement: Supplementary file 1 [file nutrients-18-02119-s001.zip › S1.Semantic segmentation graphs of different models/NO.1 -1.jpg]

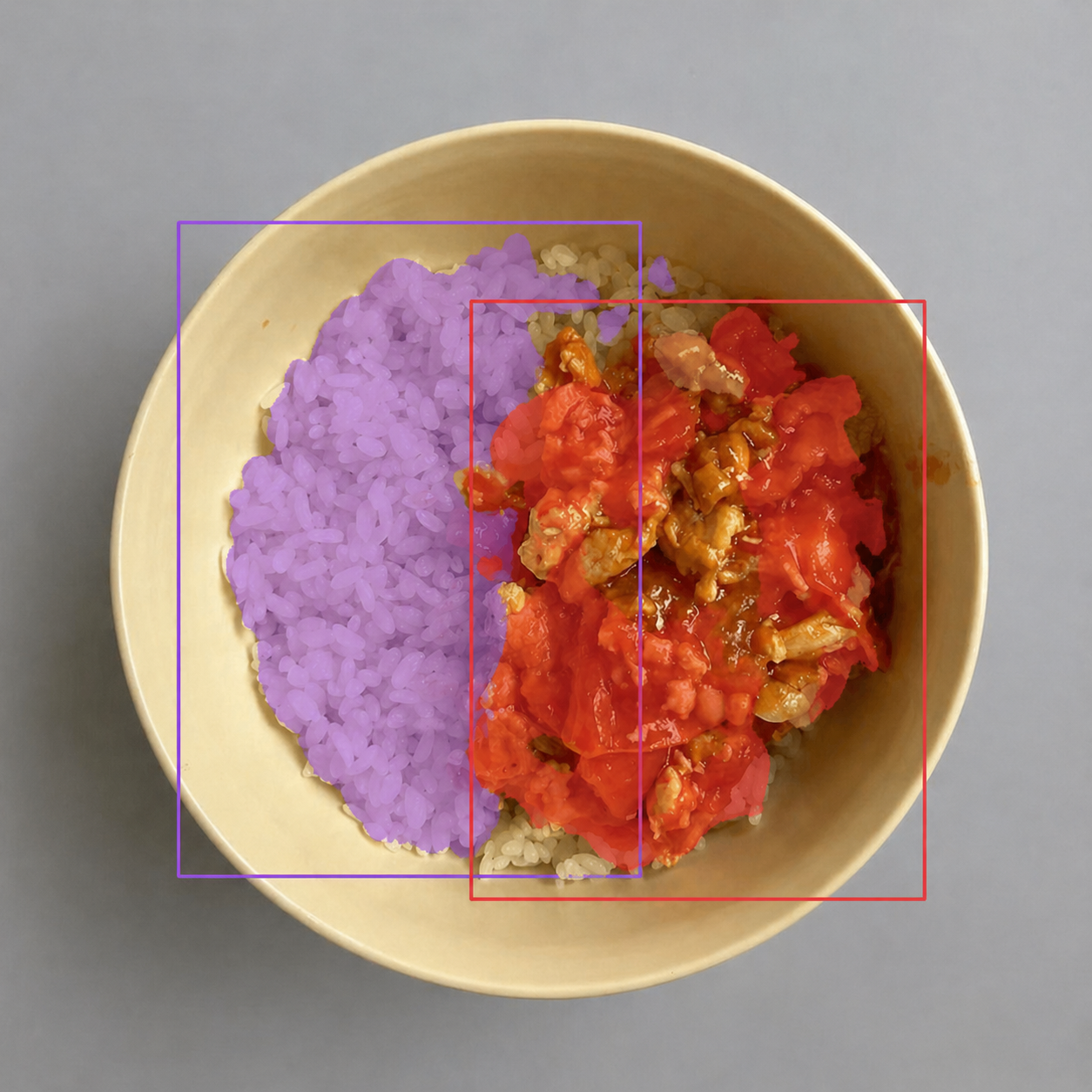

Supplement: Supplementary file 1 [file nutrients-18-02119-s001.zip › S1.Semantic segmentation graphs of different models/No.1-2.png]

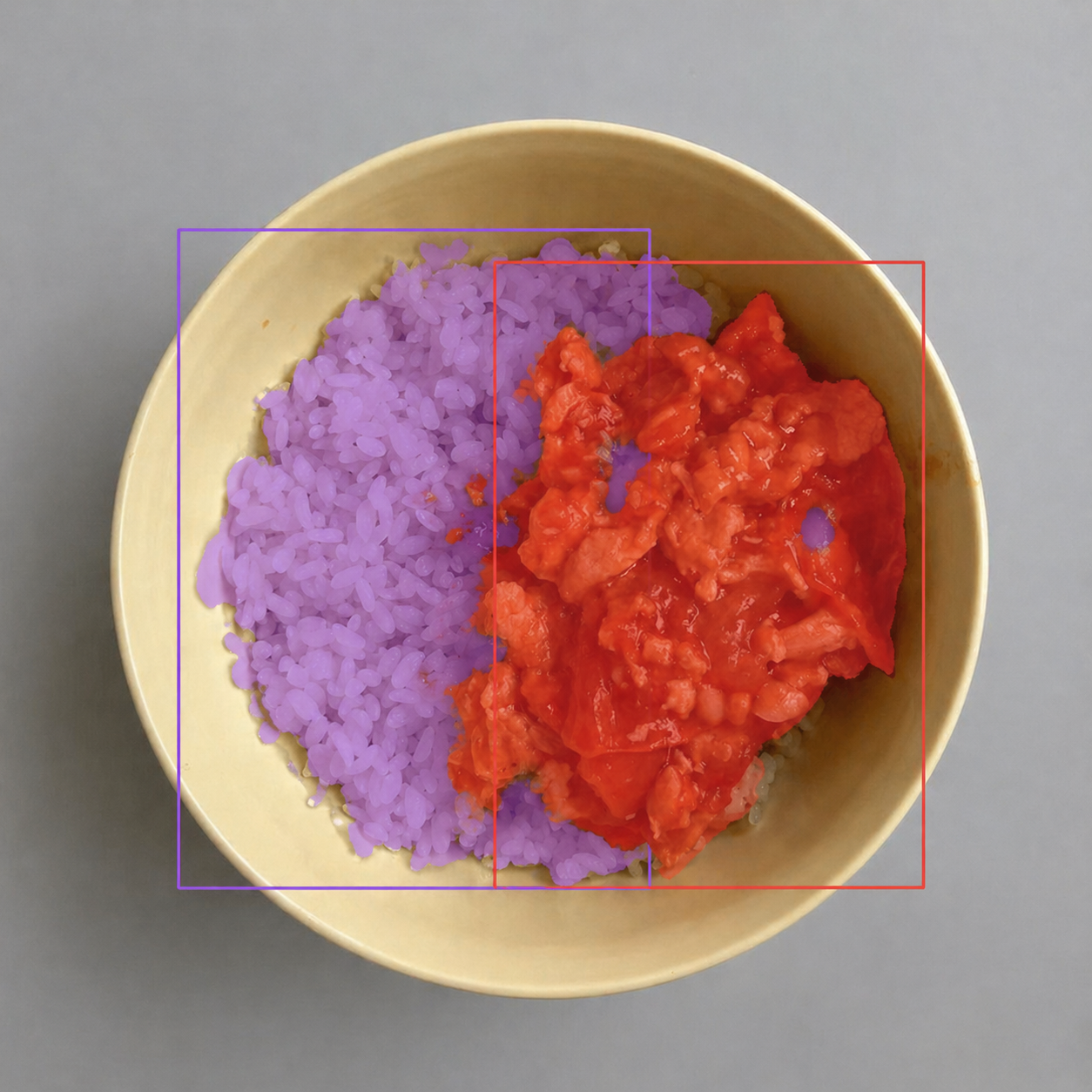

Supplement: Supplementary file 1 [file nutrients-18-02119-s001.zip › S1.Semantic segmentation graphs of different models/No.1-3.png]

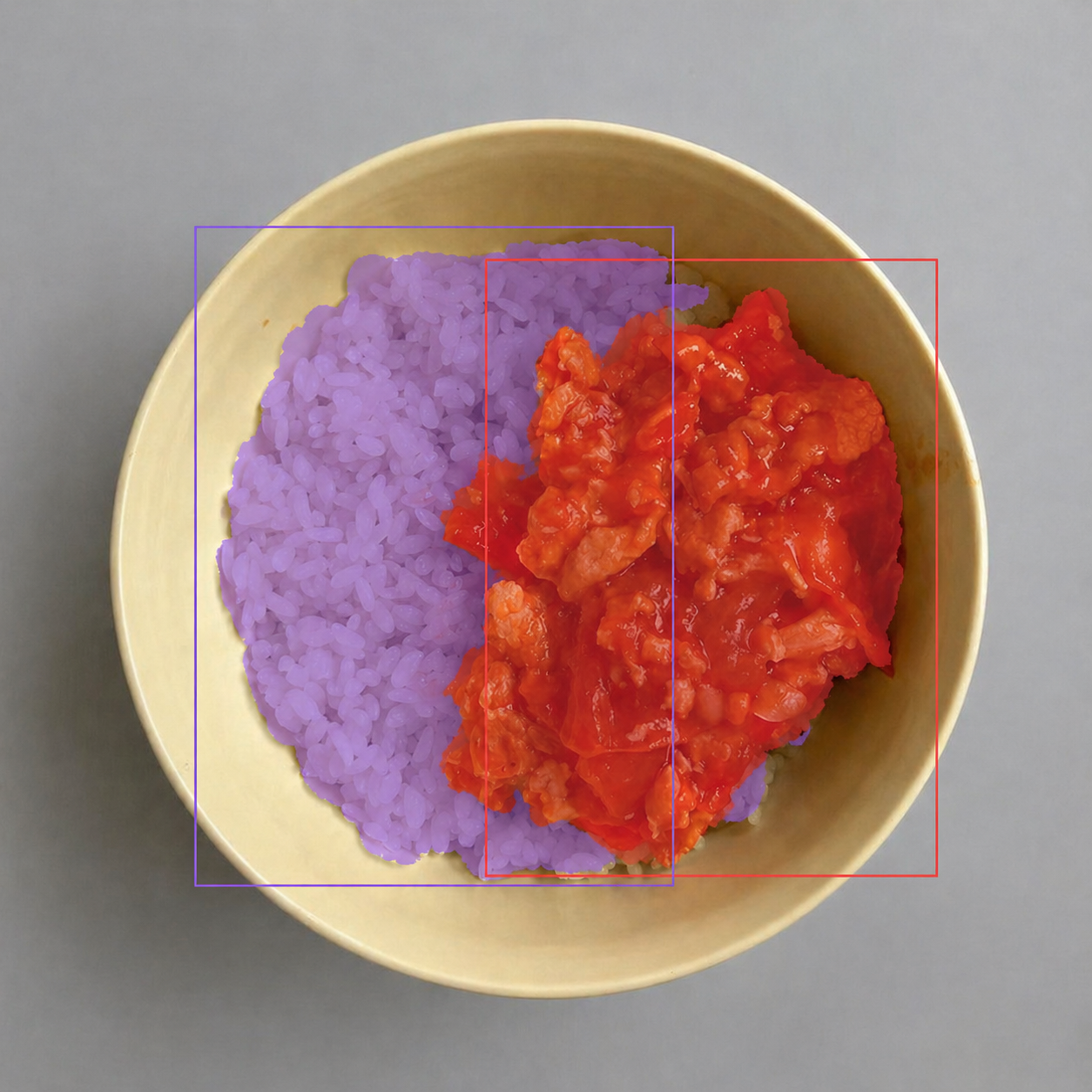

Supplement: Supplementary file 1 [file nutrients-18-02119-s001.zip › S1.Semantic segmentation graphs of different models/No.1-4.png]

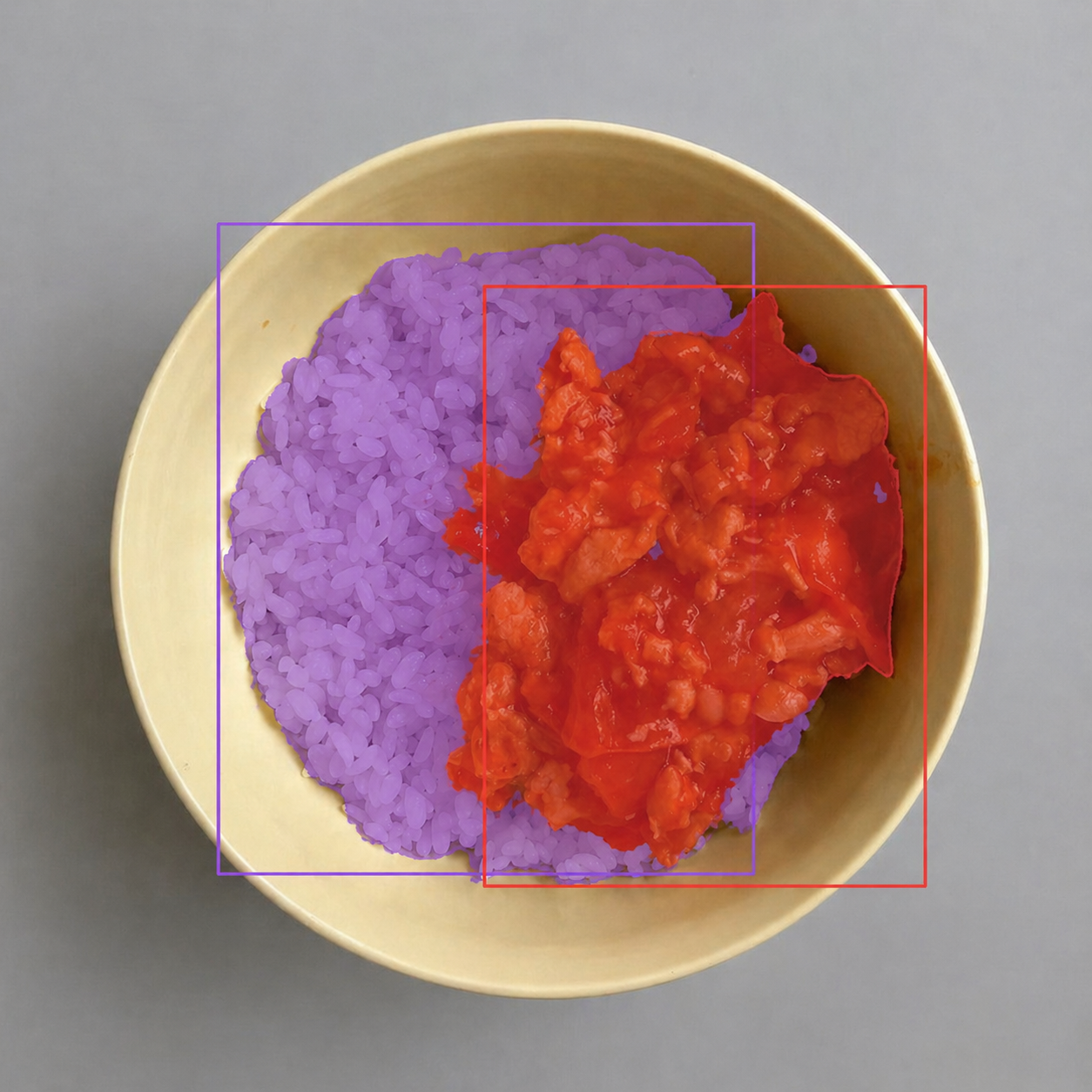

Supplement: Supplementary file 1 [file nutrients-18-02119-s001.zip › S1.Semantic segmentation graphs of different models/No.1-5.png]

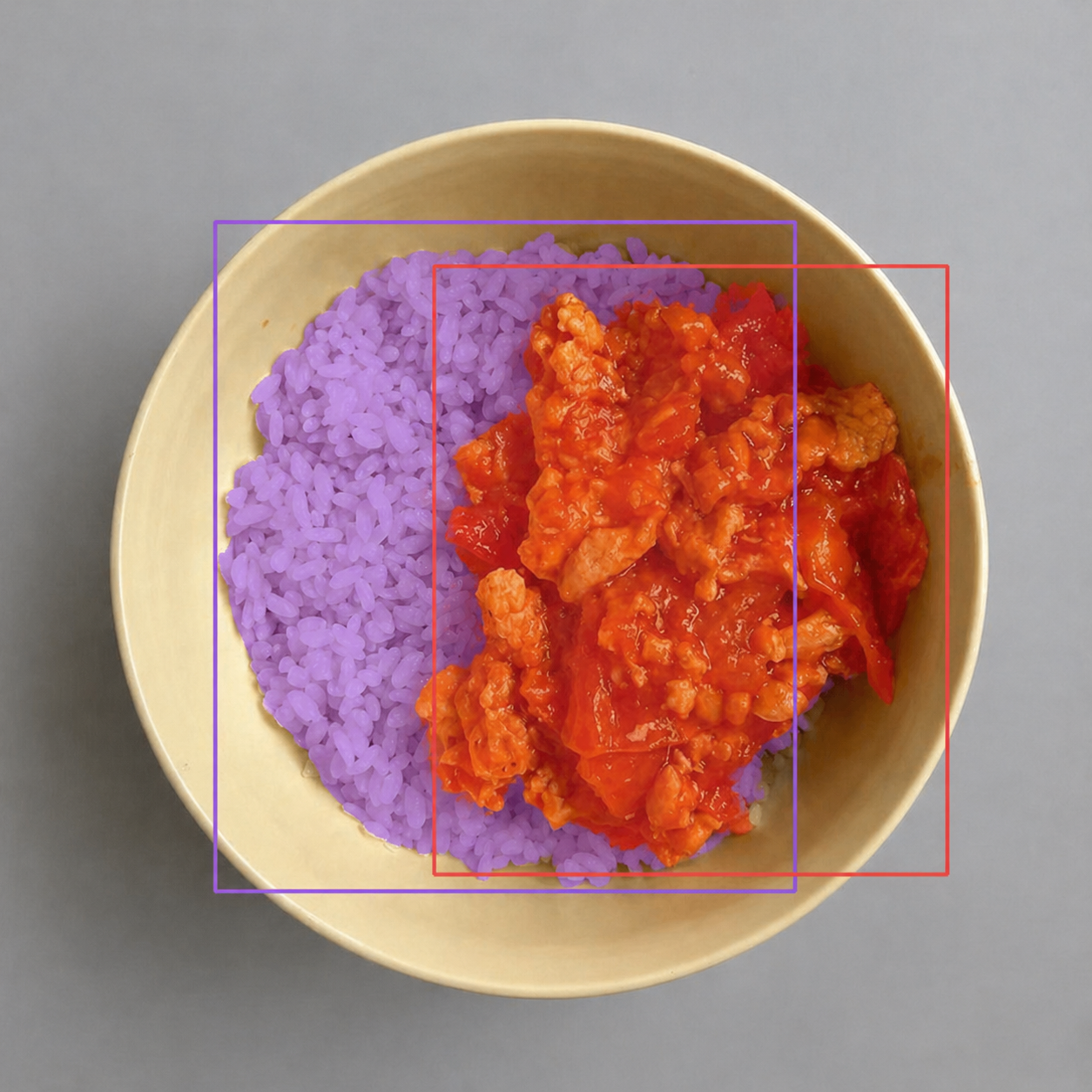

Supplement: Supplementary file 1 [file nutrients-18-02119-s001.zip › S1.Semantic segmentation graphs of different models/No.1-6.png]

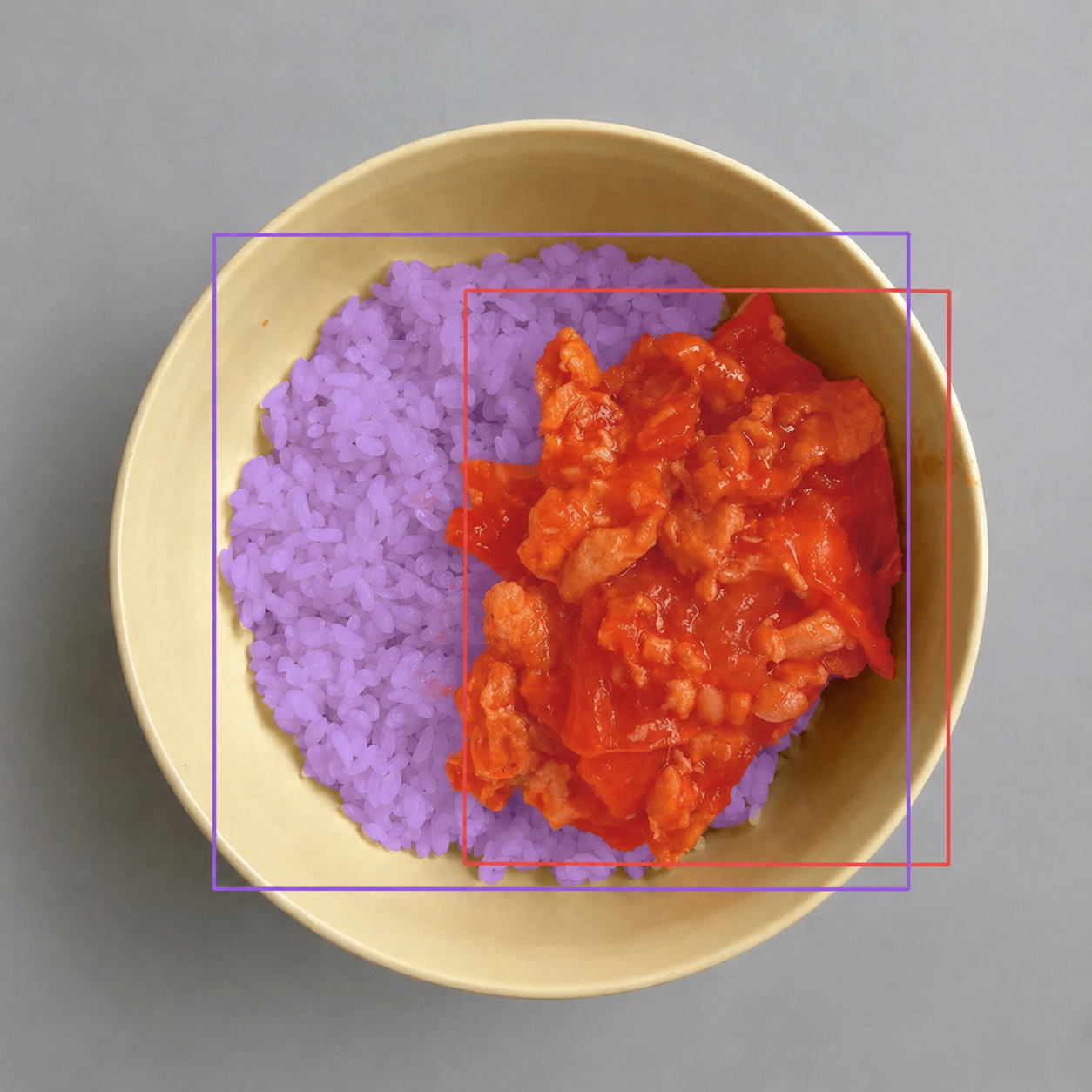

Supplement: Supplementary file 1 [file nutrients-18-02119-s001.zip › S1.Semantic segmentation graphs of different models/No.1-7.png]

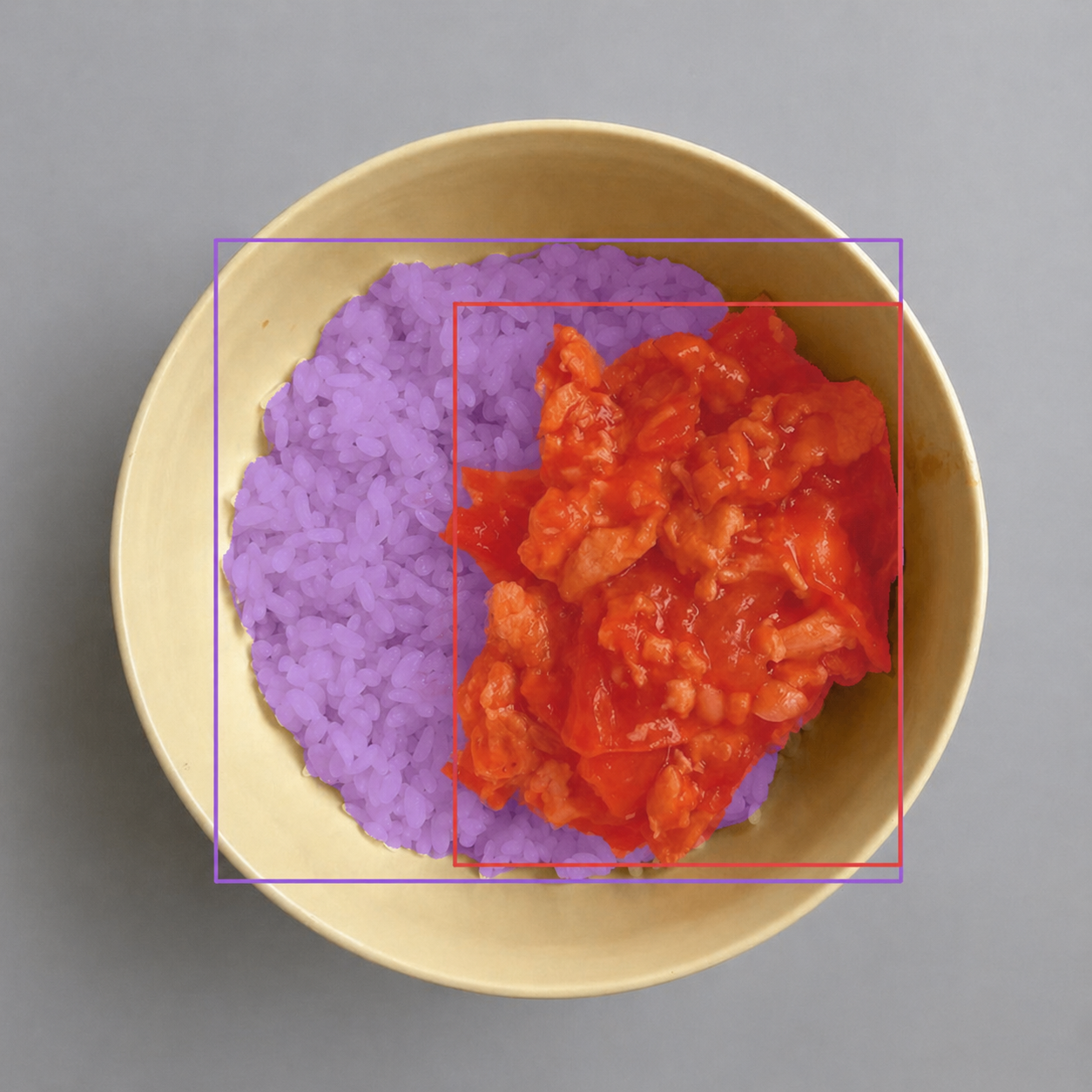

Supplement: Supplementary file 1 [file nutrients-18-02119-s001.zip › S1.Semantic segmentation graphs of different models/No.1-8.png]

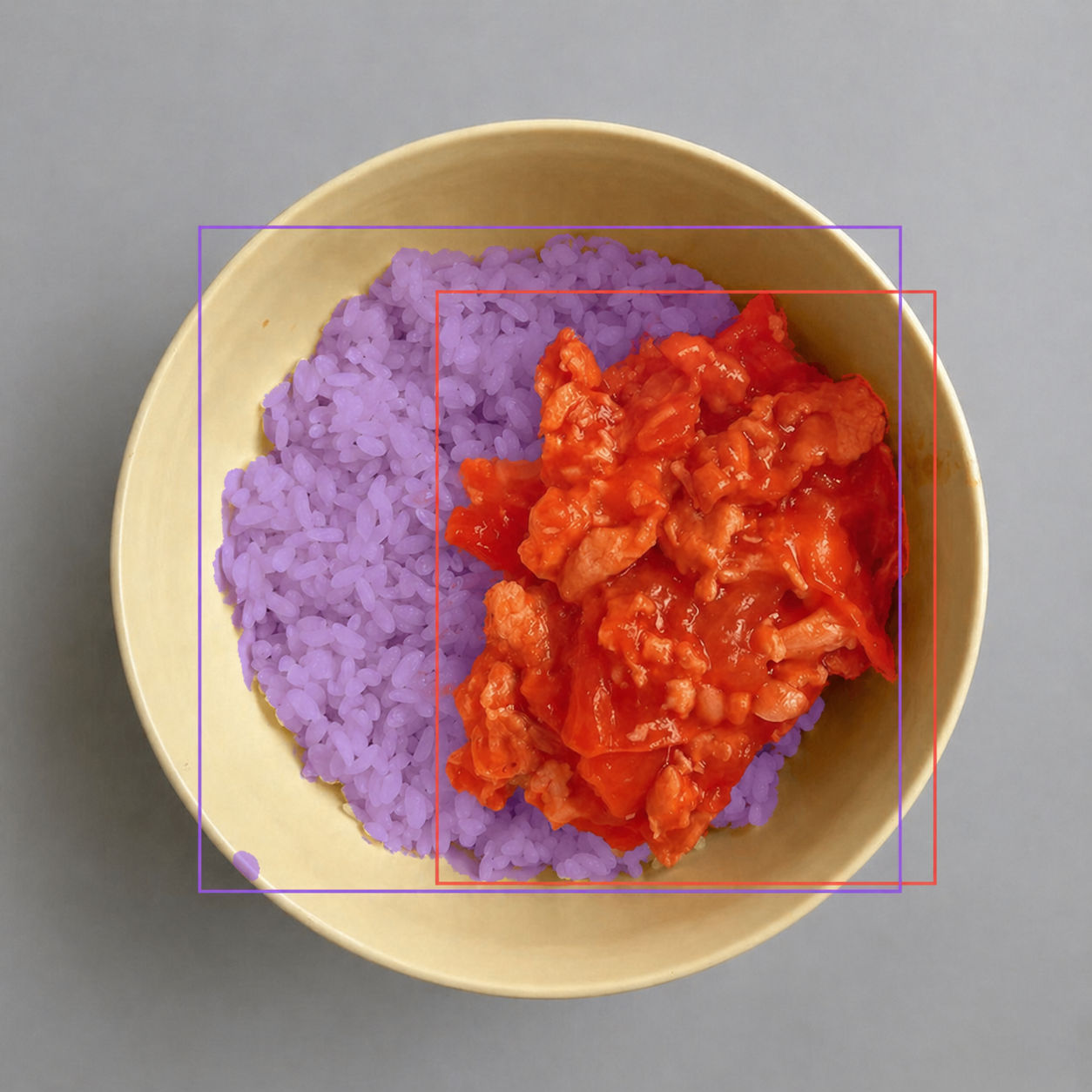

Supplement: Supplementary file 1 [file nutrients-18-02119-s001.zip › S1.Semantic segmentation graphs of different models/No.1-9.png]

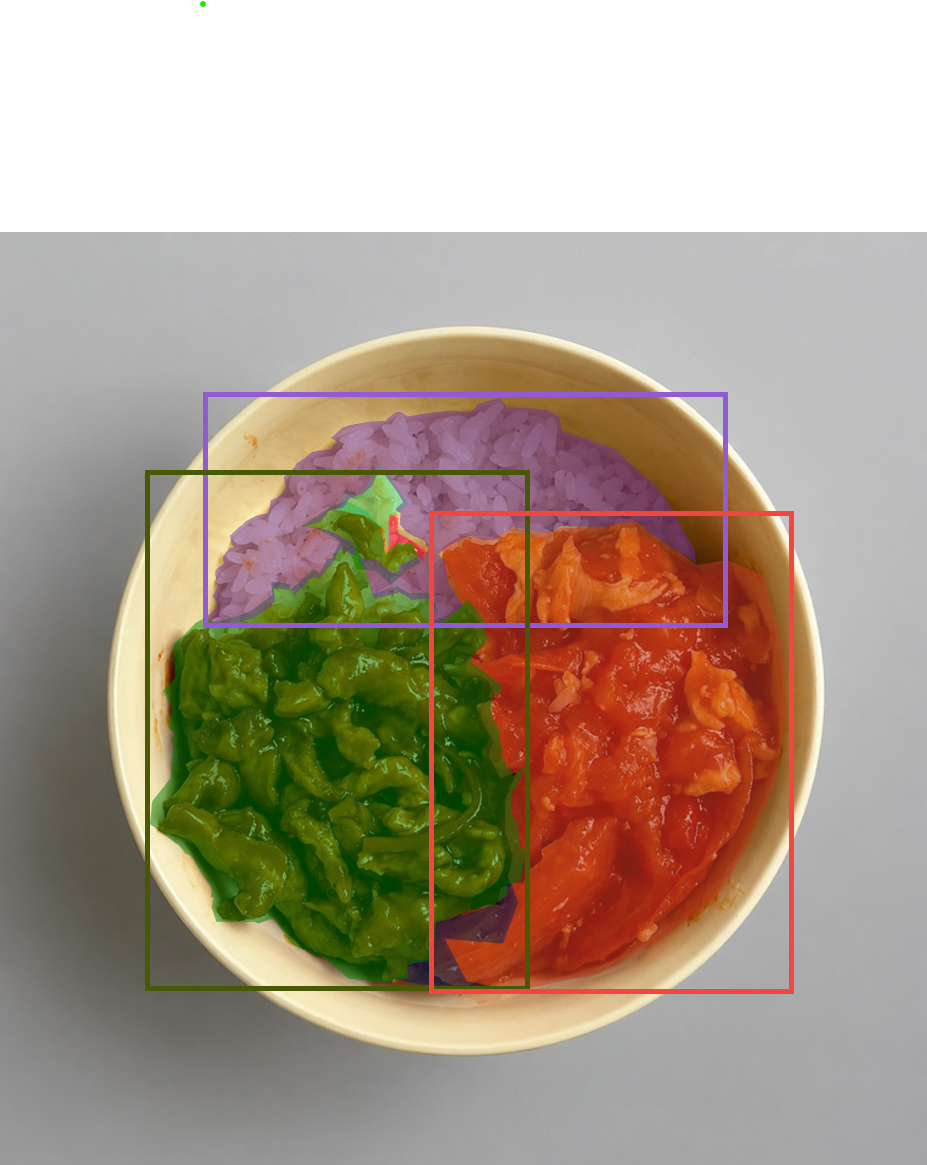

Supplement: Supplementary file 1 [file nutrients-18-02119-s001.zip › S1.Semantic segmentation graphs of different models/No.2-1.png]

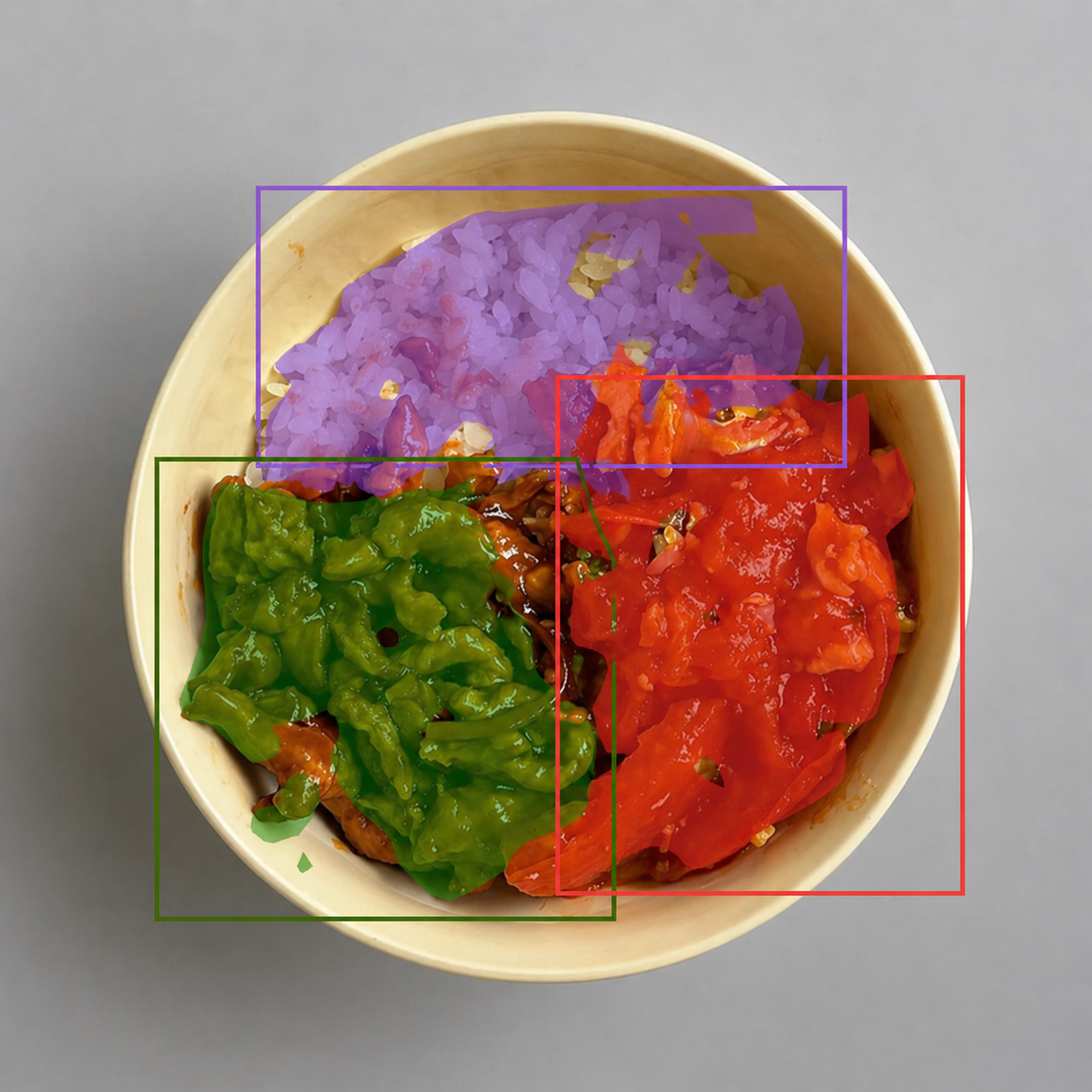

Supplement: Supplementary file 1 [file nutrients-18-02119-s001.zip › S1.Semantic segmentation graphs of different models/No.2-2.png]

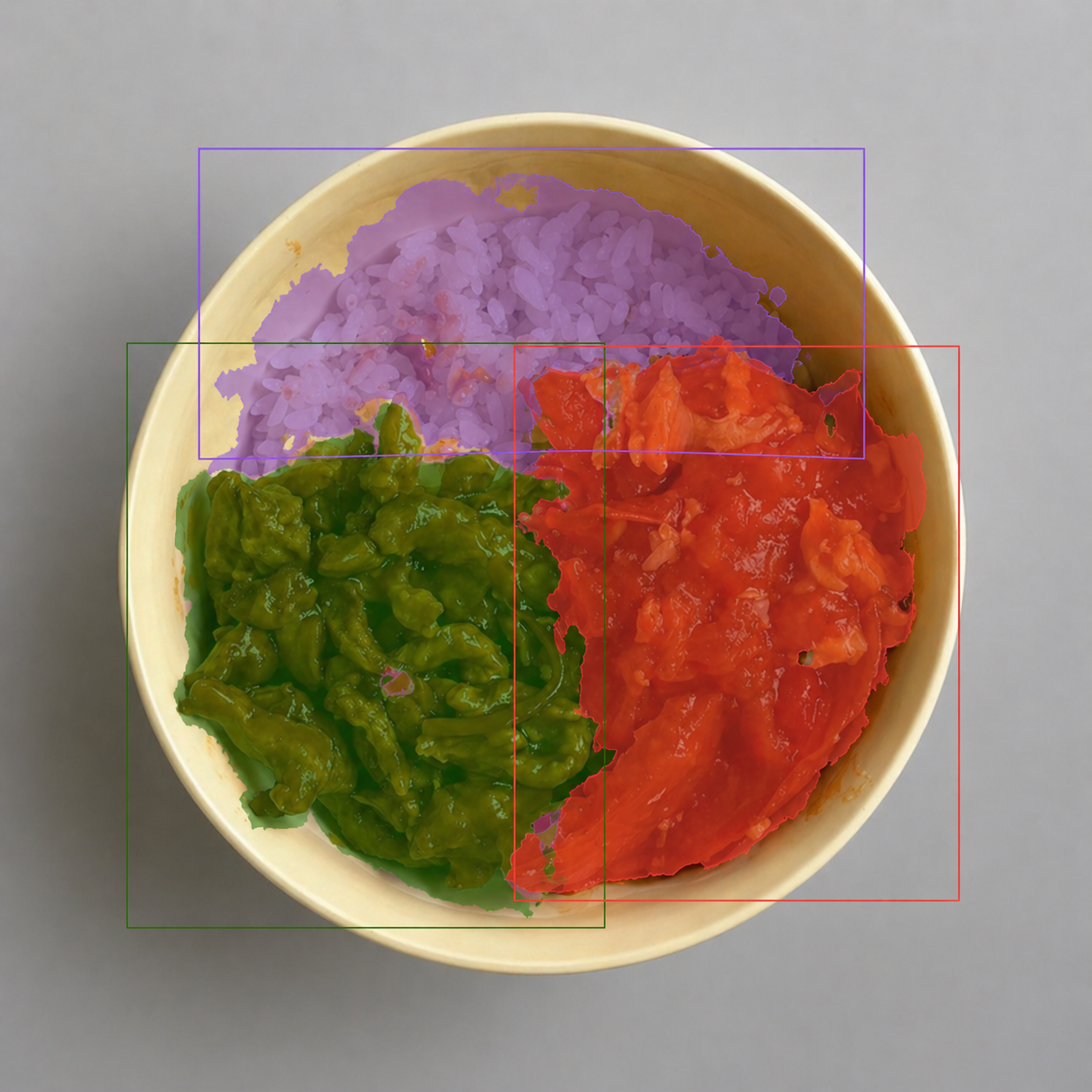

Supplement: Supplementary file 1 [file nutrients-18-02119-s001.zip › S1.Semantic segmentation graphs of different models/No.2-3.png]

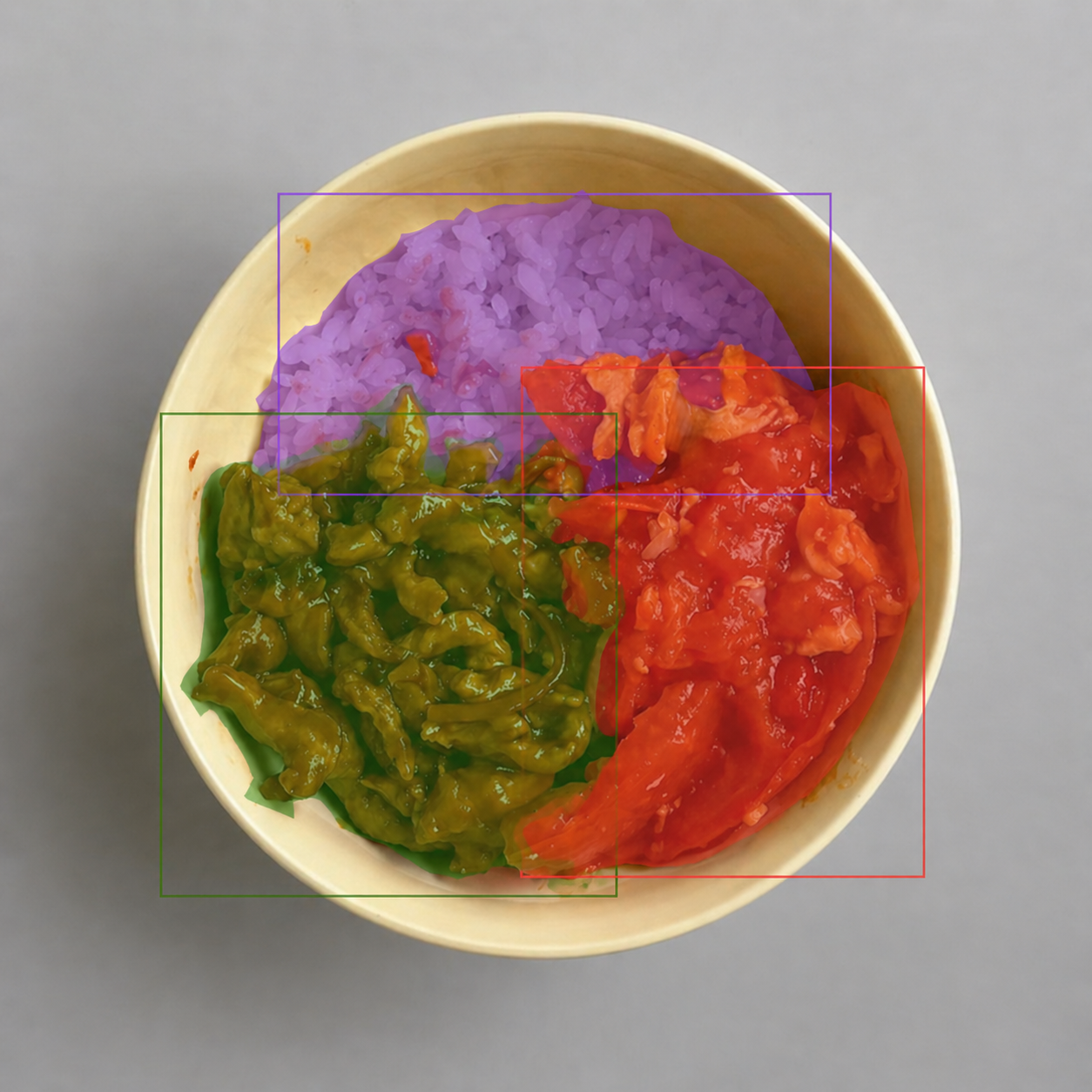

Supplement: Supplementary file 1 [file nutrients-18-02119-s001.zip › S1.Semantic segmentation graphs of different models/No.2-4.png]

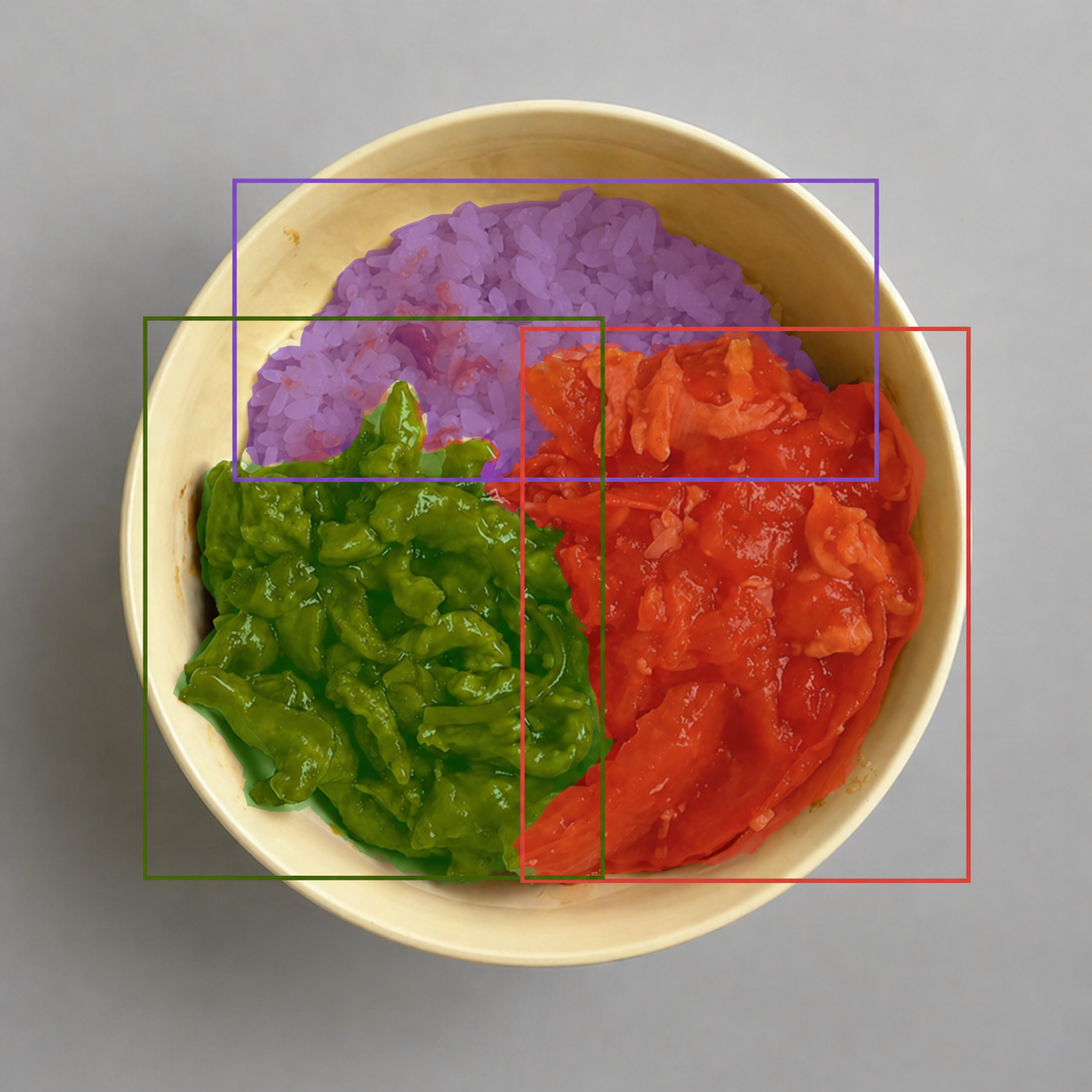

Supplement: Supplementary file 1 [file nutrients-18-02119-s001.zip › S1.Semantic segmentation graphs of different models/No.2-5.png]

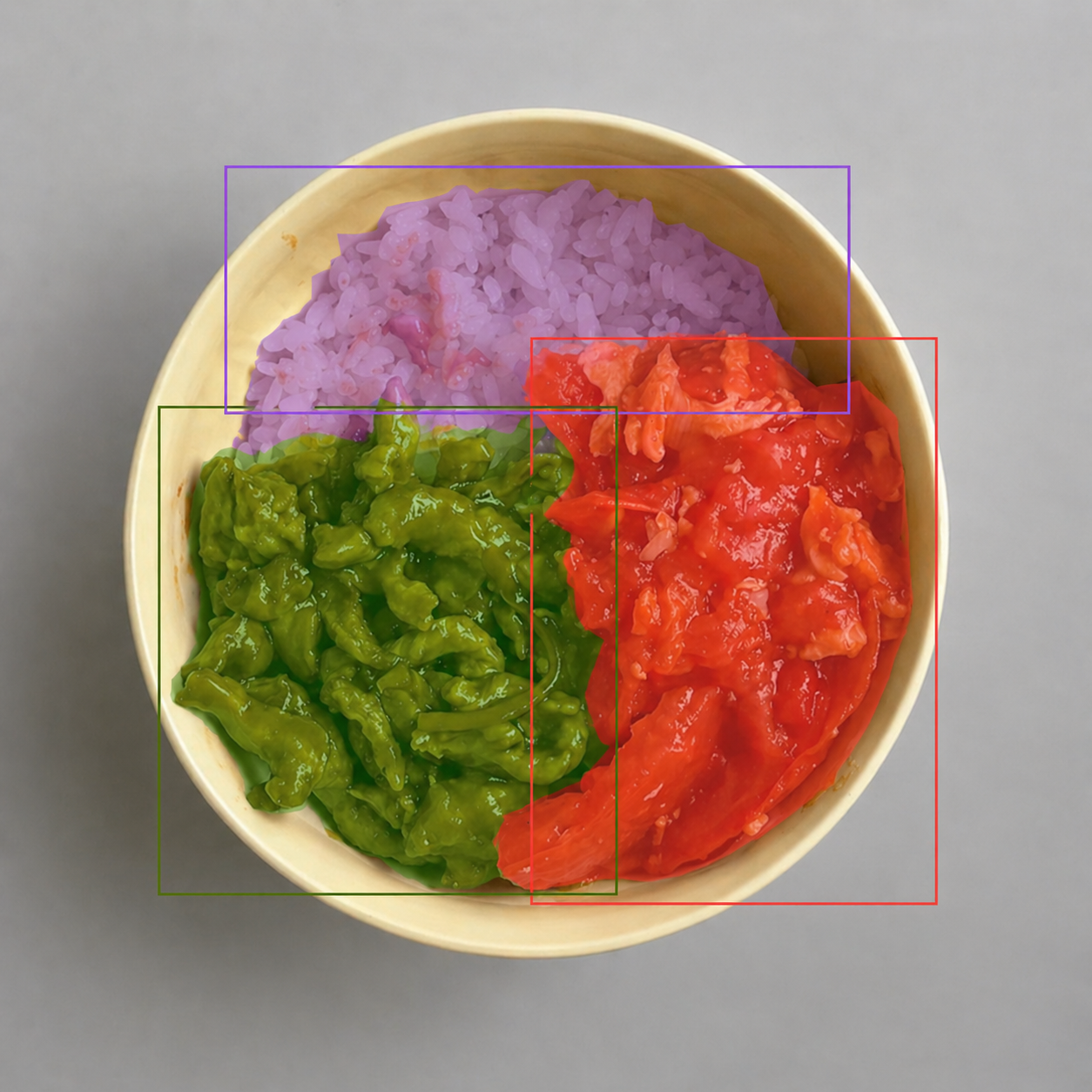

Supplement: Supplementary file 1 [file nutrients-18-02119-s001.zip › S1.Semantic segmentation graphs of different models/No.2-6.png]

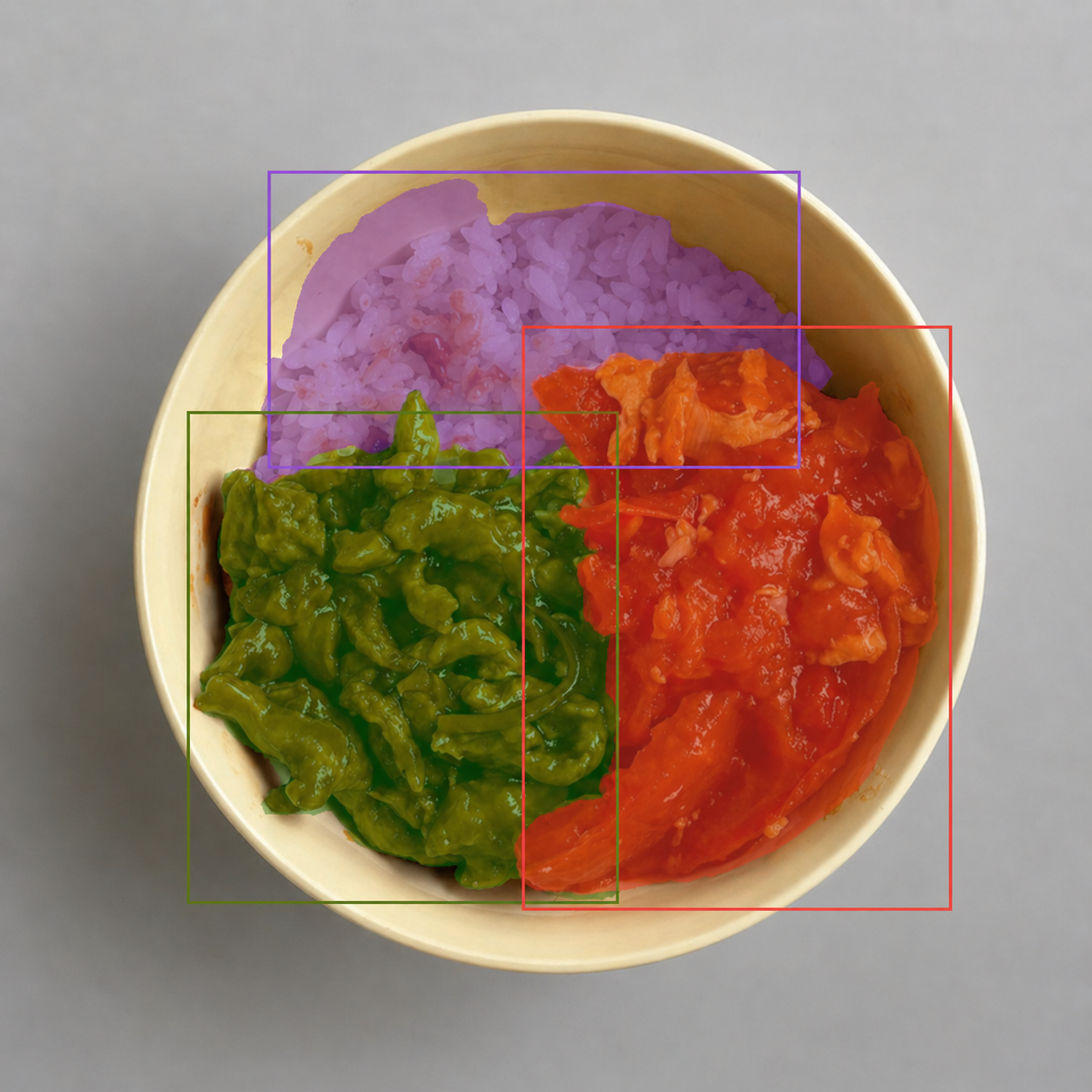

Supplement: Supplementary file 1 [file nutrients-18-02119-s001.zip › S1.Semantic segmentation graphs of different models/No.2-7.png]

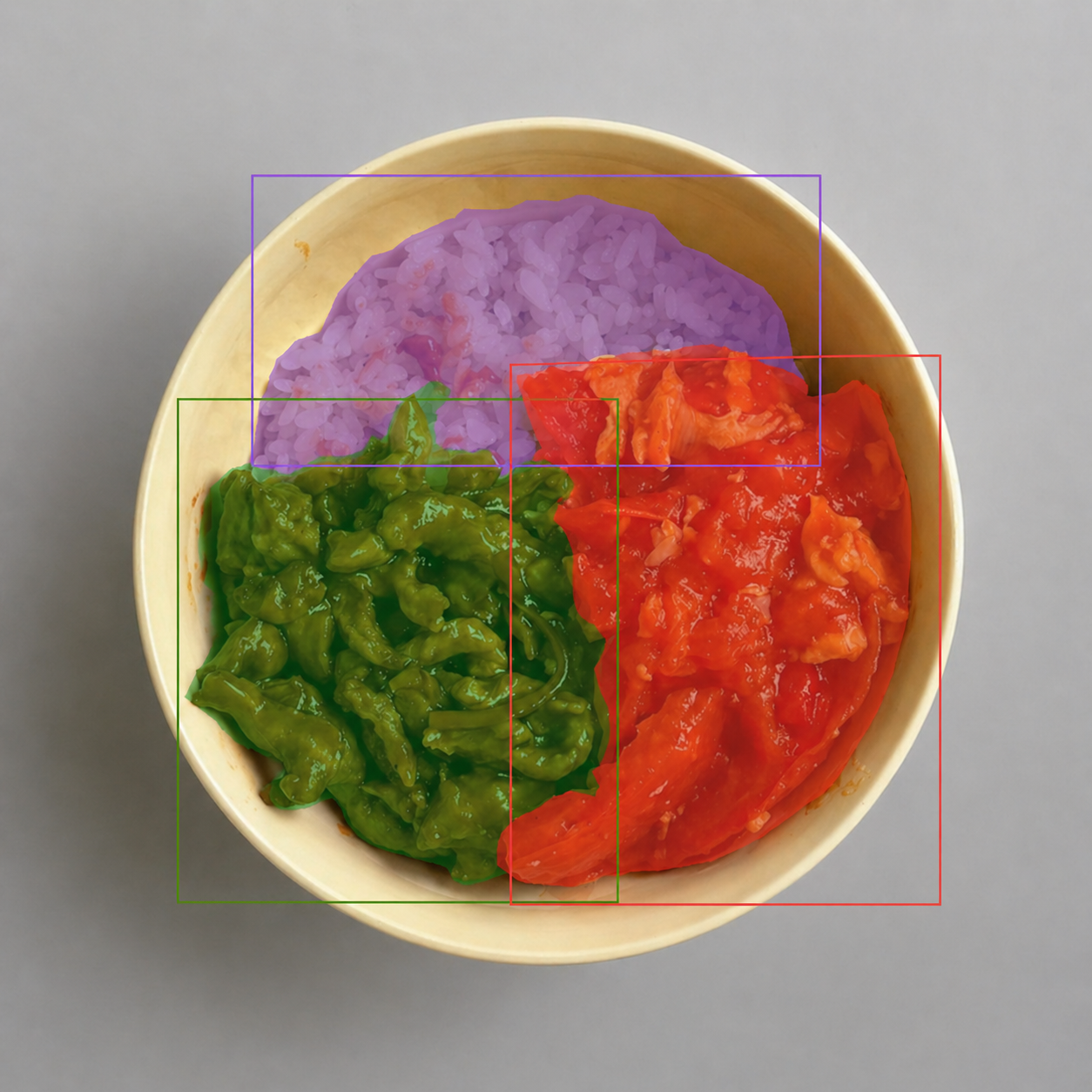

Supplement: Supplementary file 1 [file nutrients-18-02119-s001.zip › S1.Semantic segmentation graphs of different models/No.2-8.png]

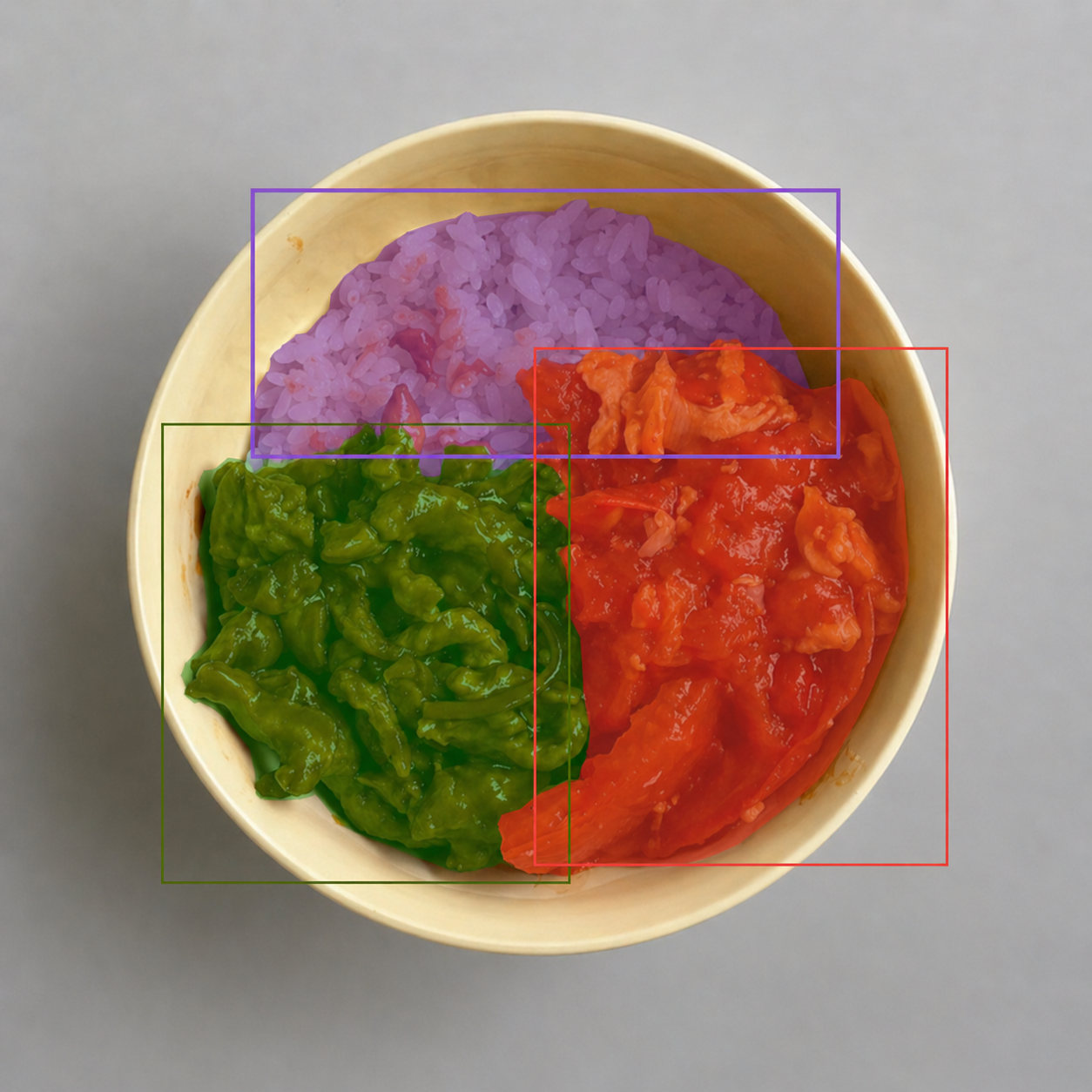

Supplement: Supplementary file 1 [file nutrients-18-02119-s001.zip › S1.Semantic segmentation graphs of different models/No.2-9.png]

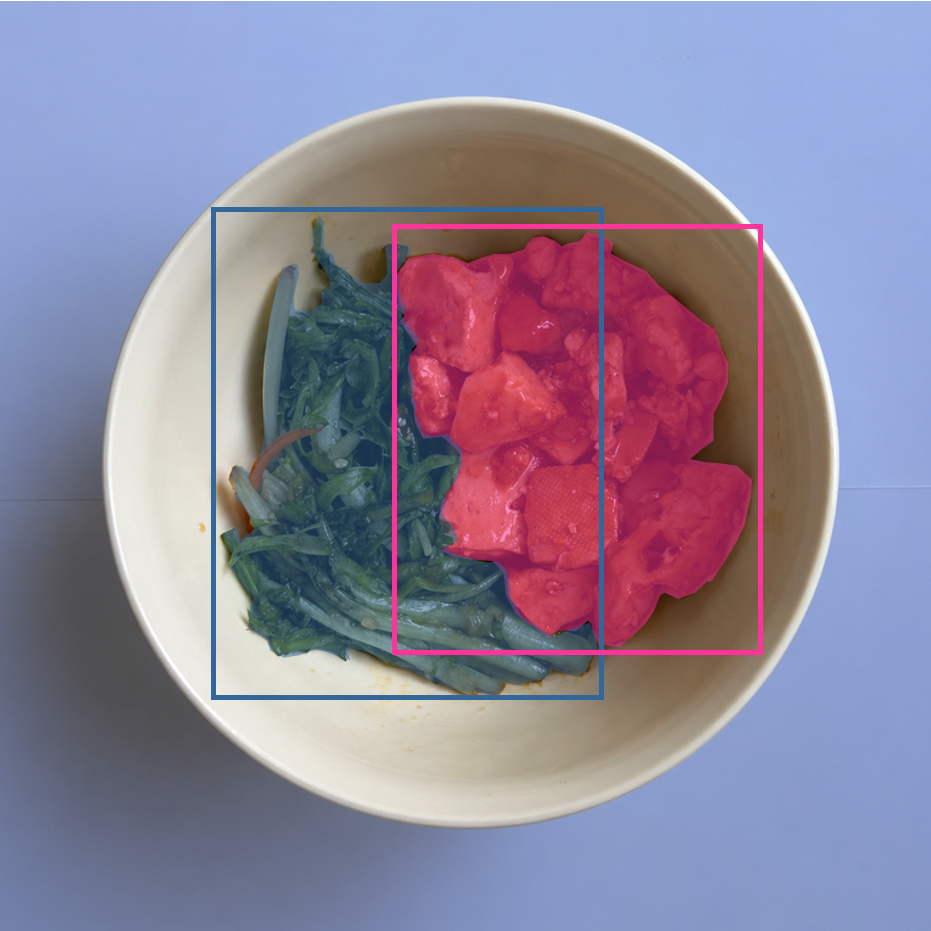

Supplement: Supplementary file 1 [file nutrients-18-02119-s001.zip › S1.Semantic segmentation graphs of different models/No.3-1.png]

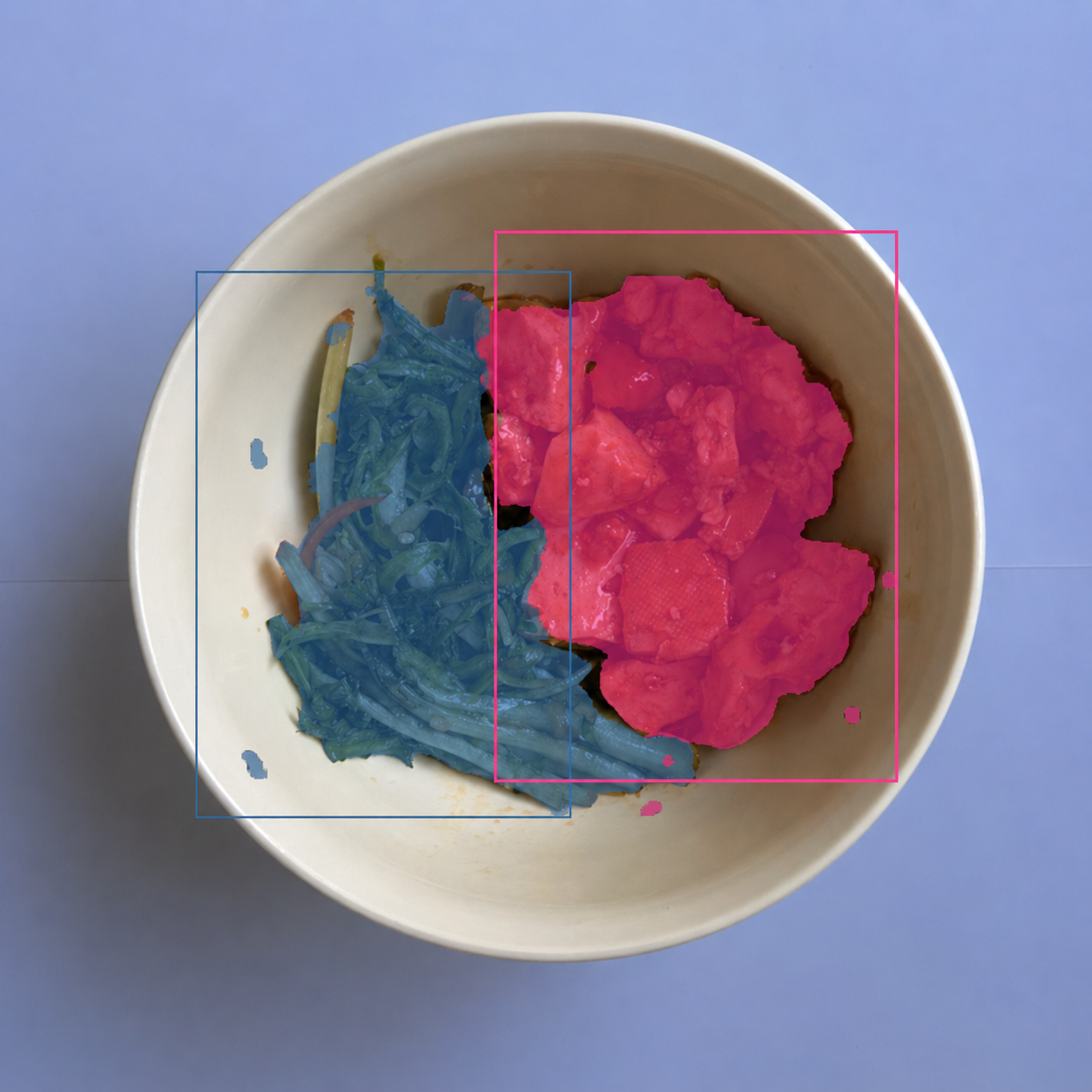

Supplement: Supplementary file 1 [file nutrients-18-02119-s001.zip › S1.Semantic segmentation graphs of different models/No.3-2.png]

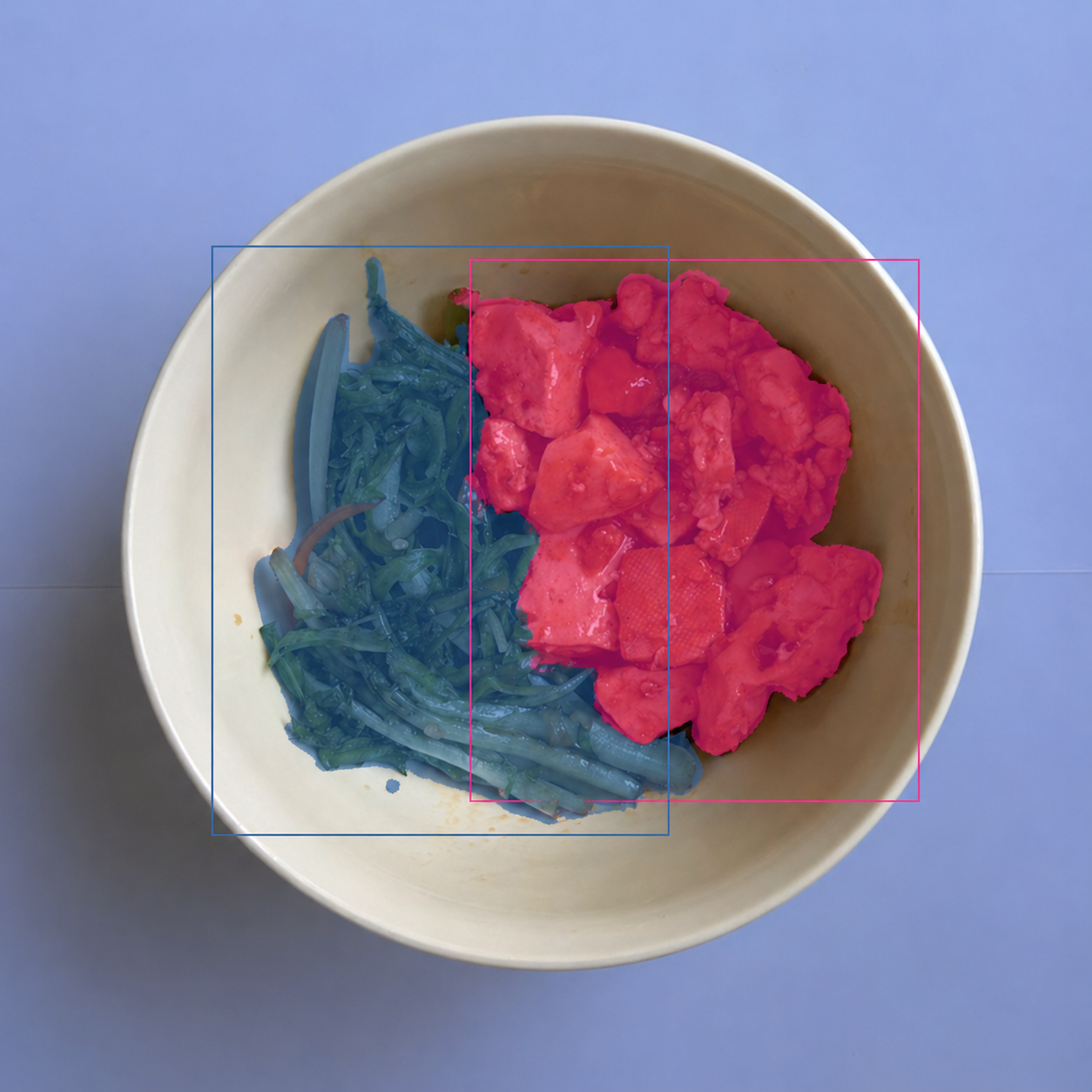

Supplement: Supplementary file 1 [file nutrients-18-02119-s001.zip › S1.Semantic segmentation graphs of different models/No.3-3.png]

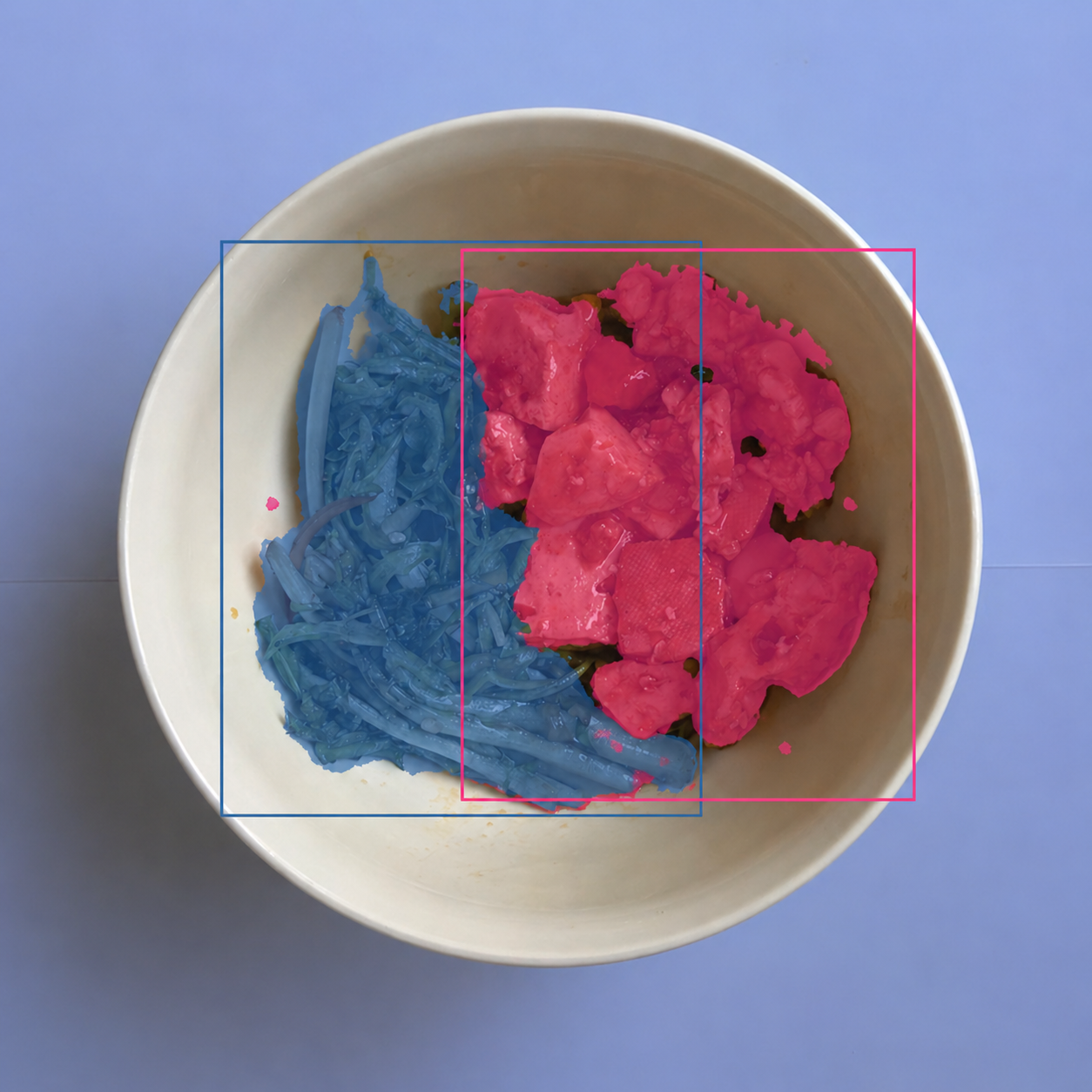

Supplement: Supplementary file 1 [file nutrients-18-02119-s001.zip › S1.Semantic segmentation graphs of different models/No.3-4.png]

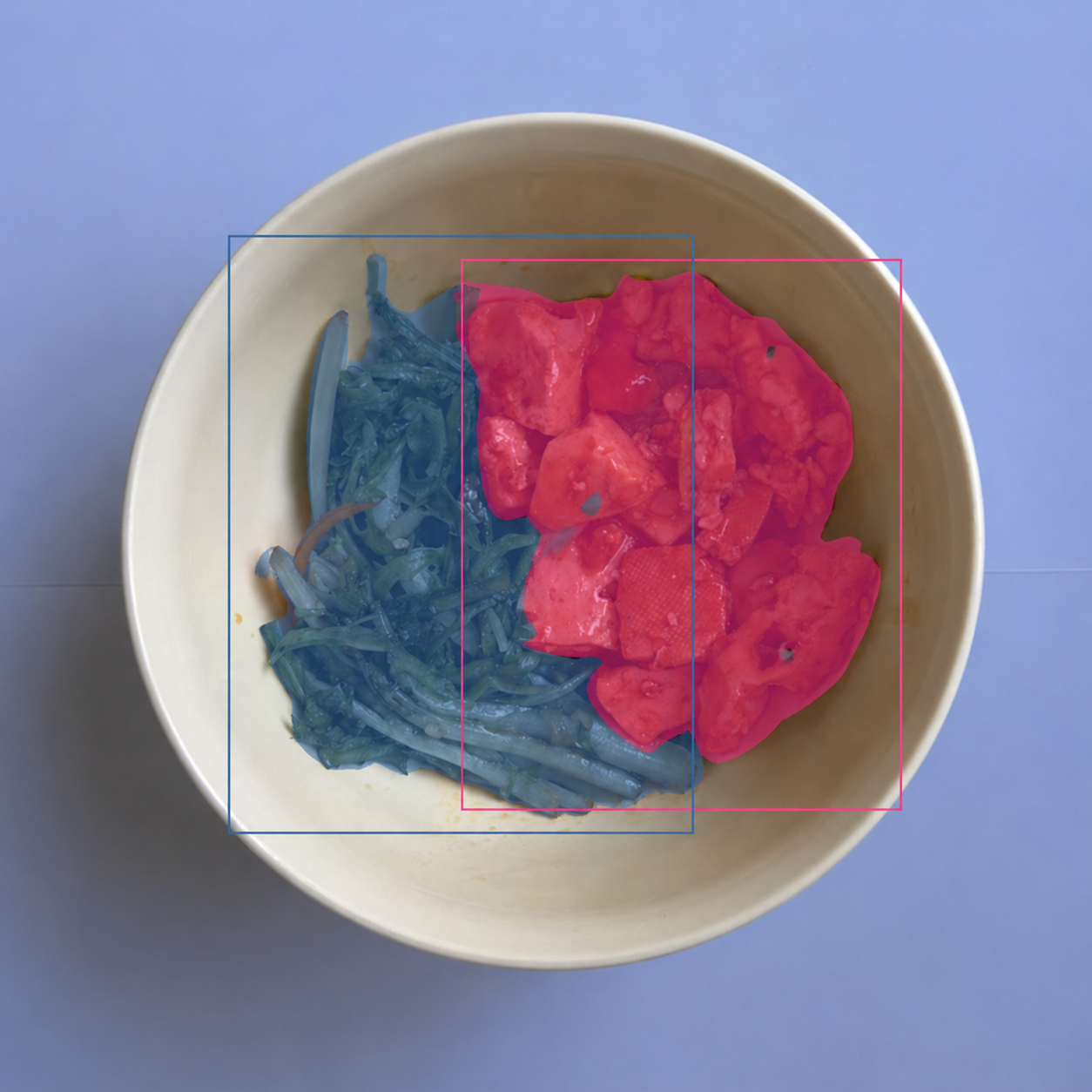

Supplement: Supplementary file 1 [file nutrients-18-02119-s001.zip › S1.Semantic segmentation graphs of different models/No.3-5.png]

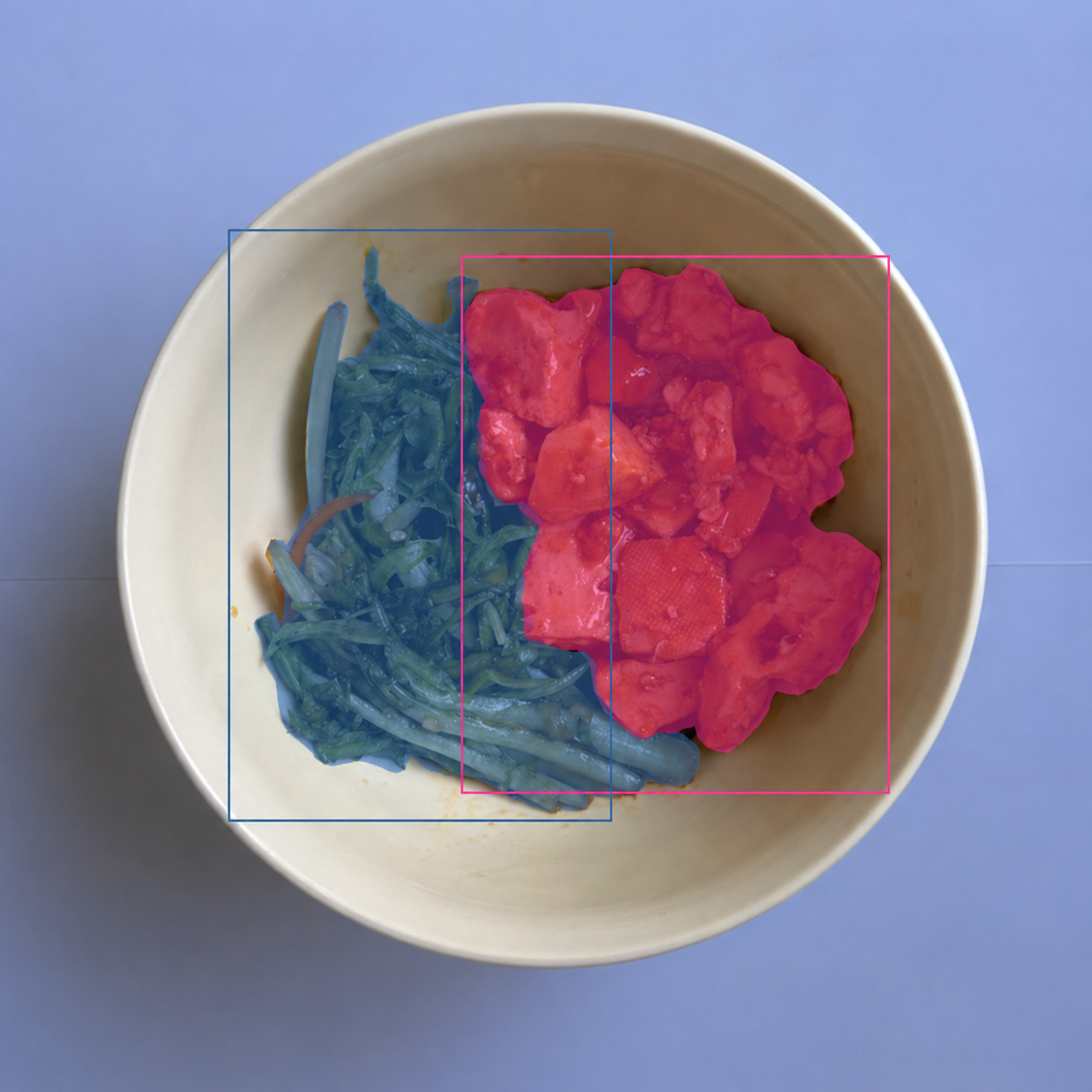

Supplement: Supplementary file 1 [file nutrients-18-02119-s001.zip › S1.Semantic segmentation graphs of different models/No.3-6.png]

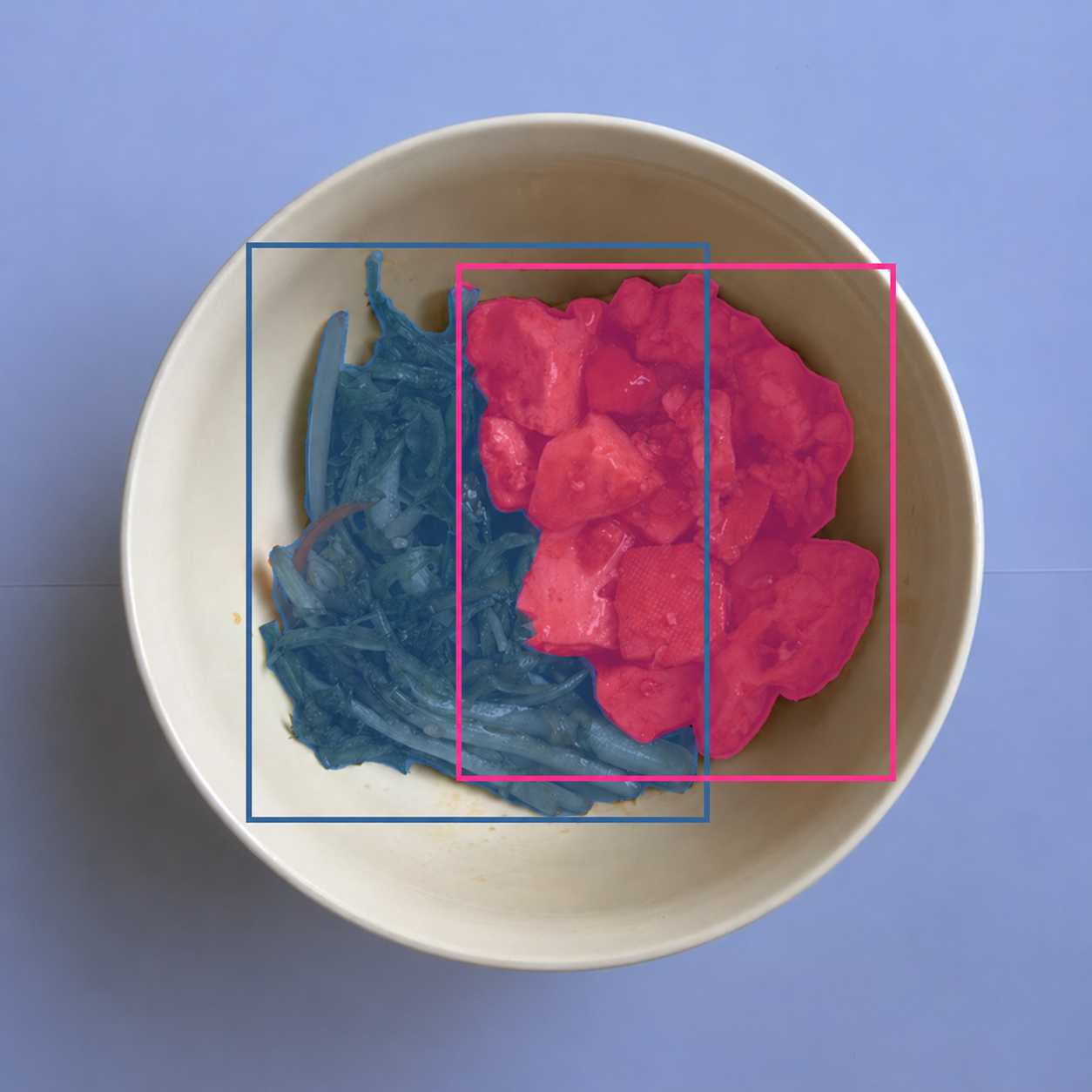

Supplement: Supplementary file 1 [file nutrients-18-02119-s001.zip › S1.Semantic segmentation graphs of different models/No.3-7.png]

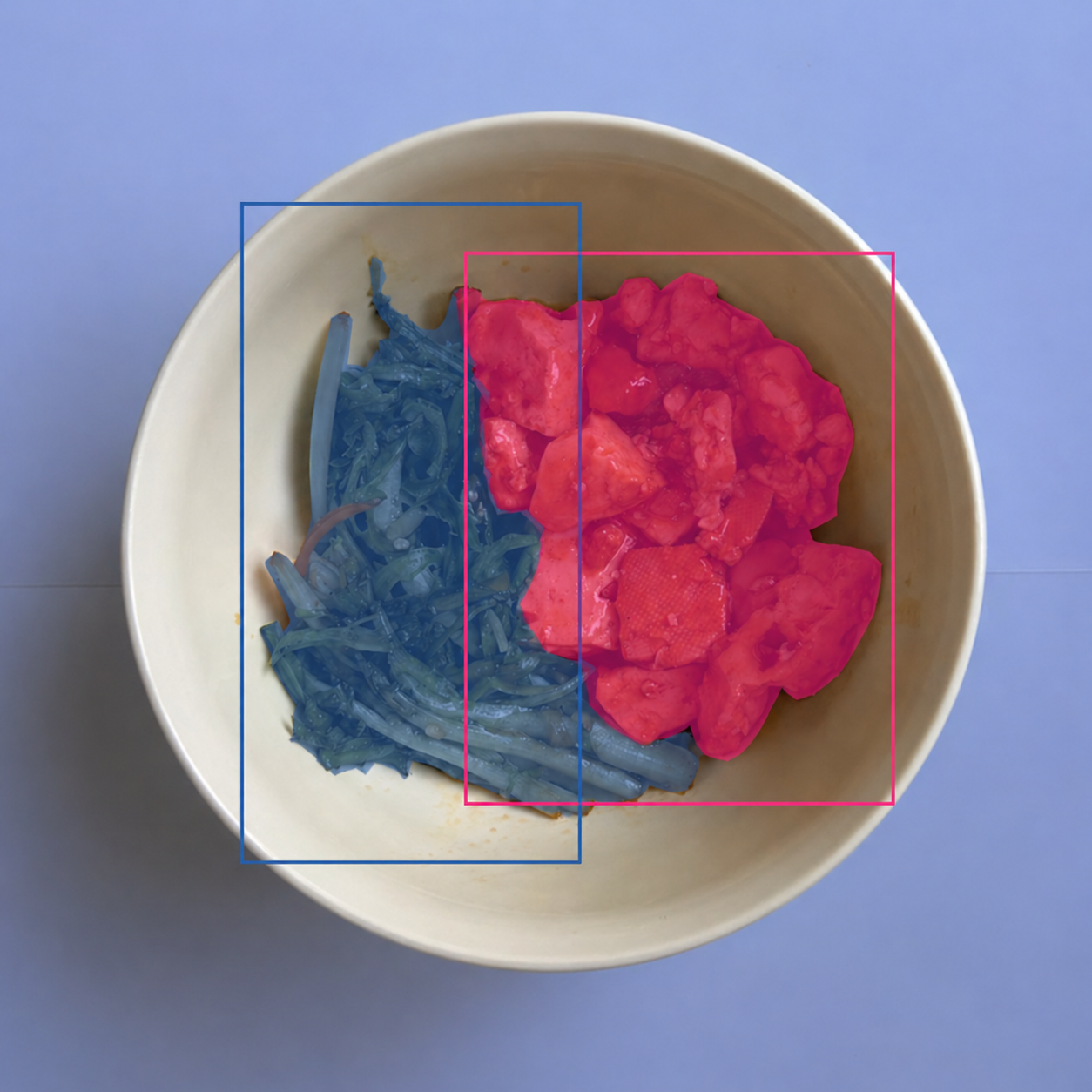

Supplement: Supplementary file 1 [file nutrients-18-02119-s001.zip › S1.Semantic segmentation graphs of different models/No.3-8.png]

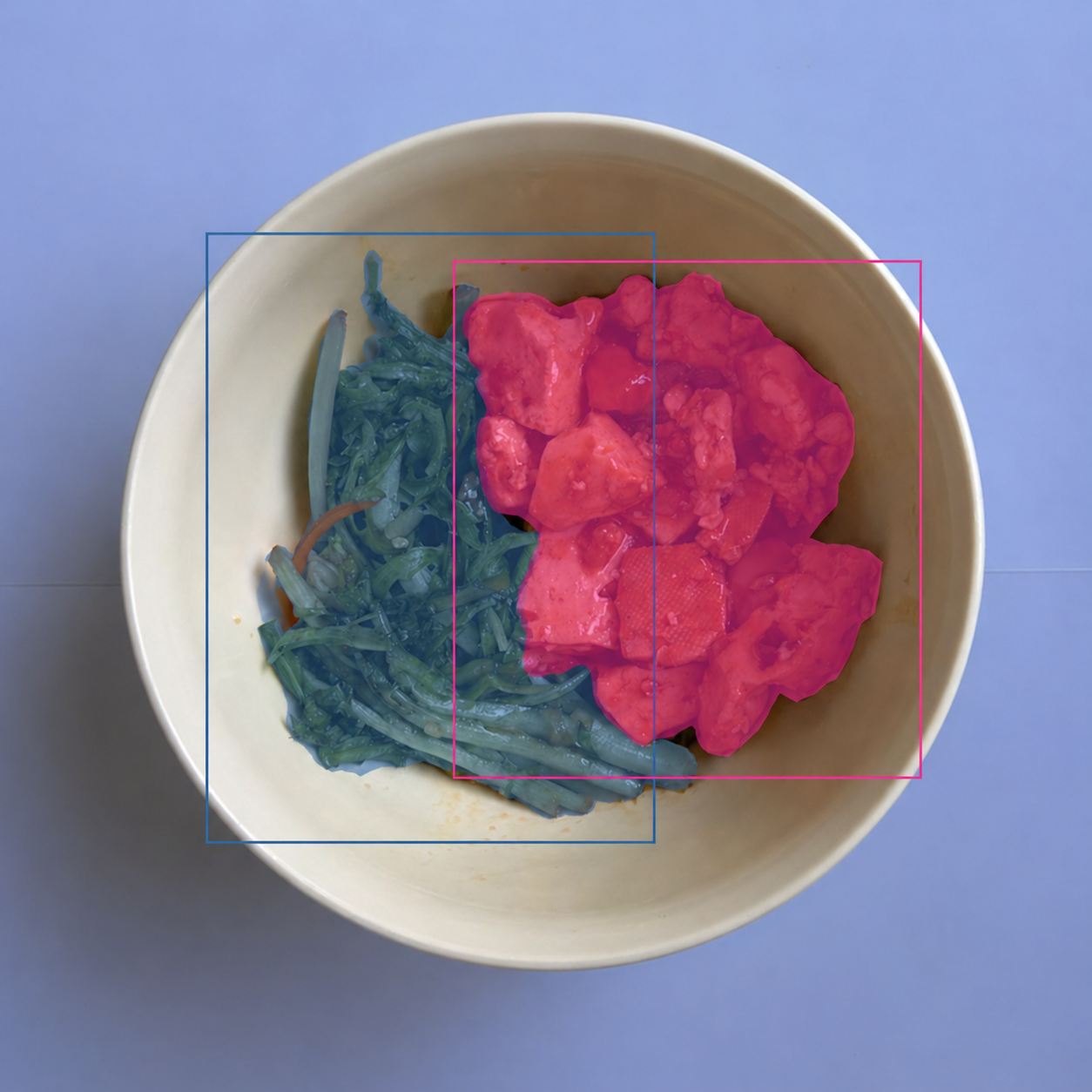

Supplement: Supplementary file 1 [file nutrients-18-02119-s001.zip › S1.Semantic segmentation graphs of different models/No.3-9.png]

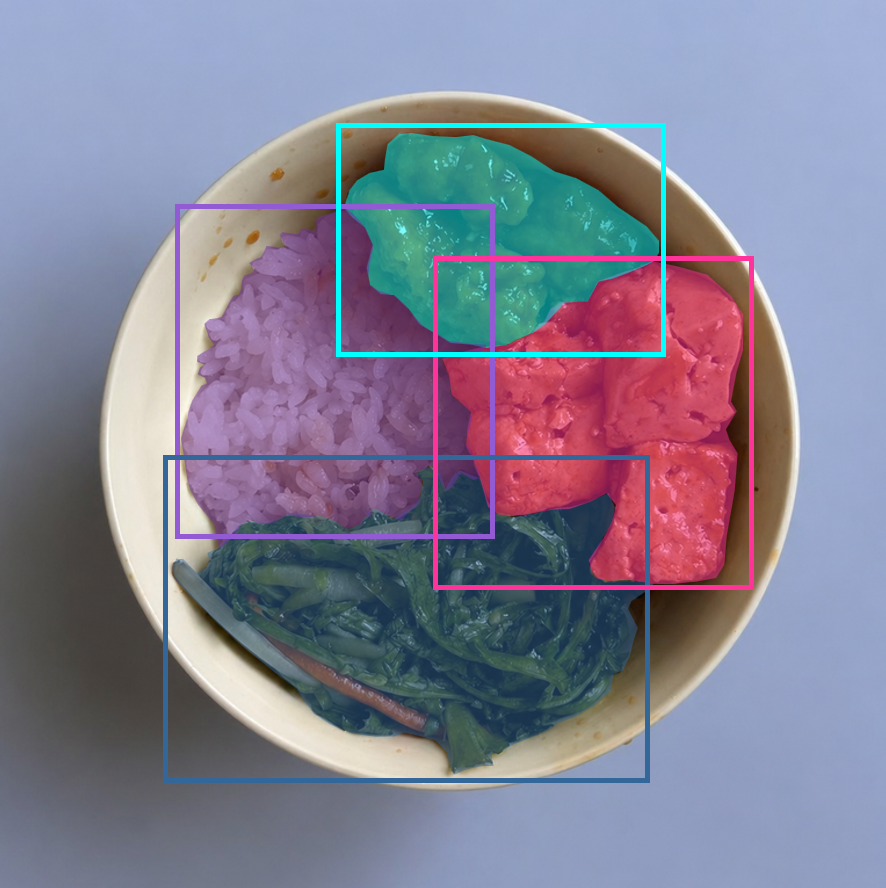

Supplement: Supplementary file 1 [file nutrients-18-02119-s001.zip › S1.Semantic segmentation graphs of different models/No.4-1.png]

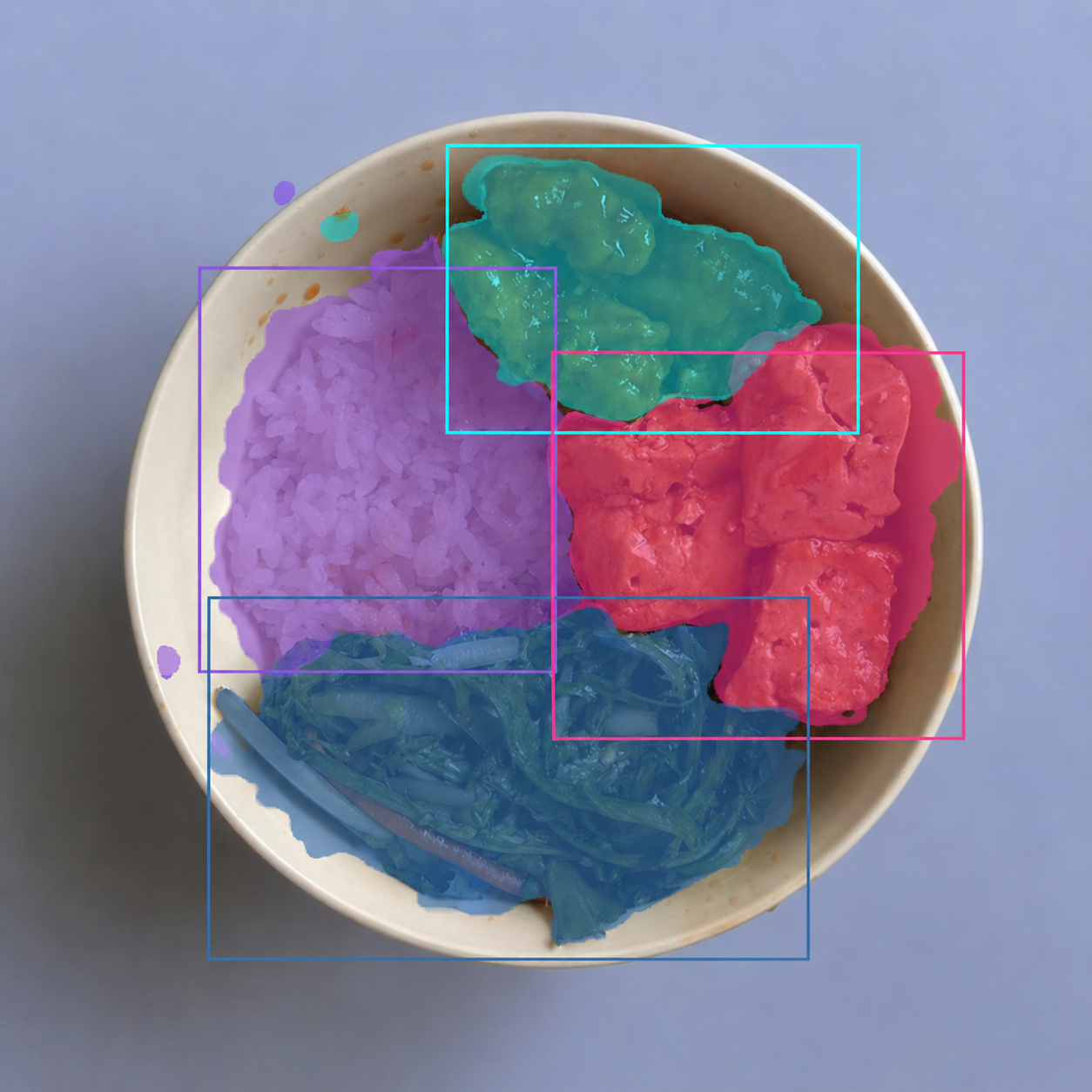

Supplement: Supplementary file 1 [file nutrients-18-02119-s001.zip › S1.Semantic segmentation graphs of different models/No.4-2.png]

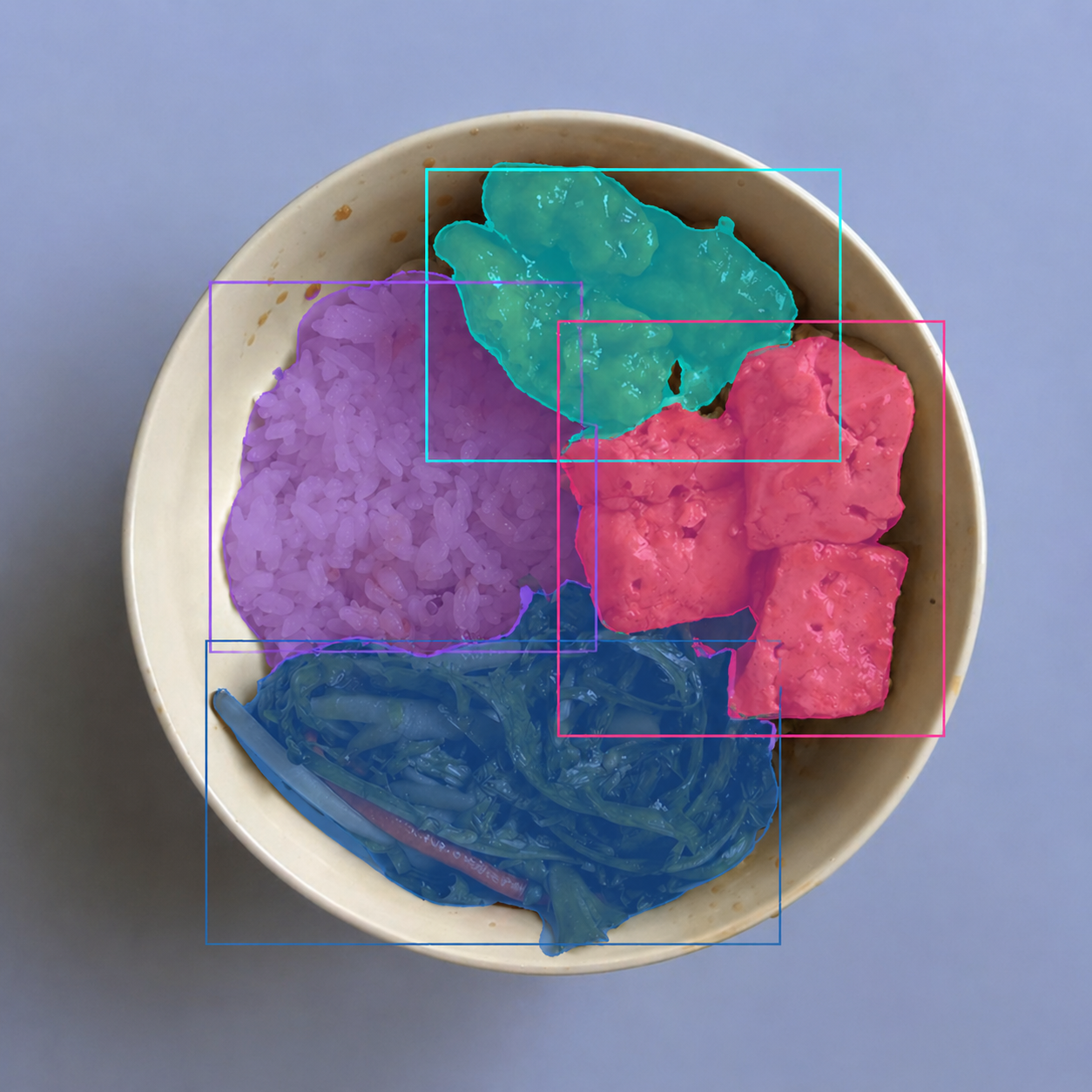

Supplement: Supplementary file 1 [file nutrients-18-02119-s001.zip › S1.Semantic segmentation graphs of different models/No.4-3.png]

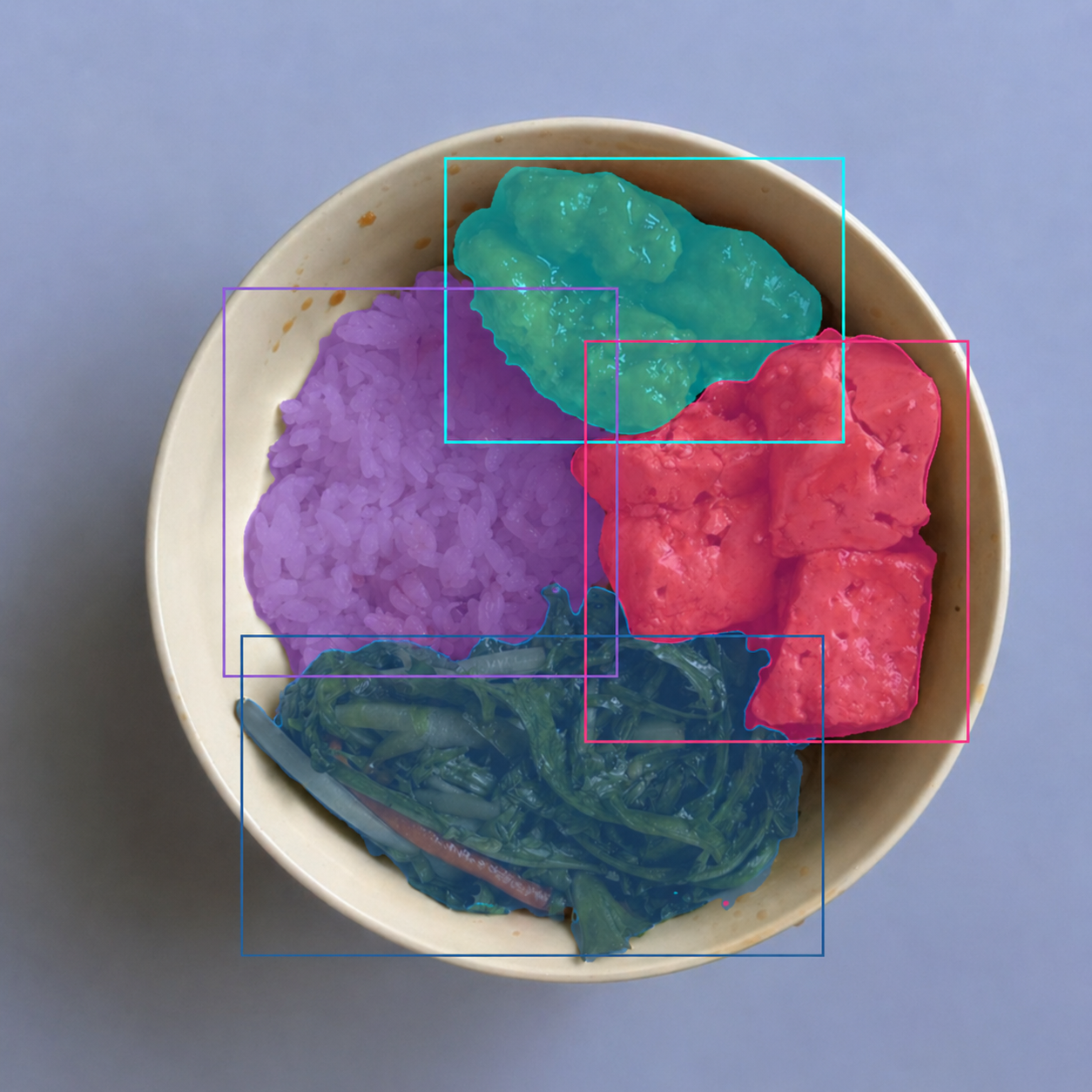

Supplement: Supplementary file 1 [file nutrients-18-02119-s001.zip › S1.Semantic segmentation graphs of different models/No.4-4.png]

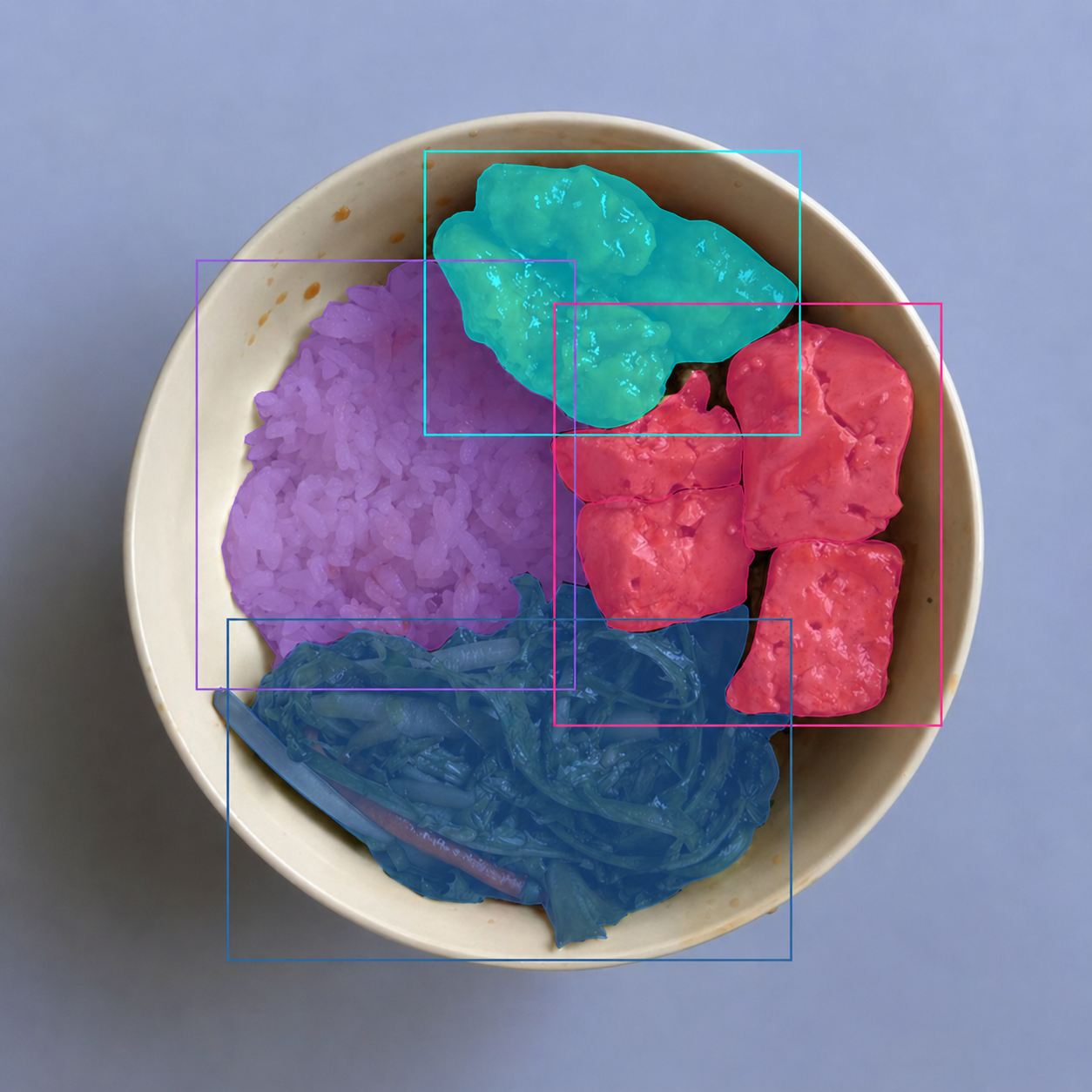

Supplement: Supplementary file 1 [file nutrients-18-02119-s001.zip › S1.Semantic segmentation graphs of different models/No.4-5.png]

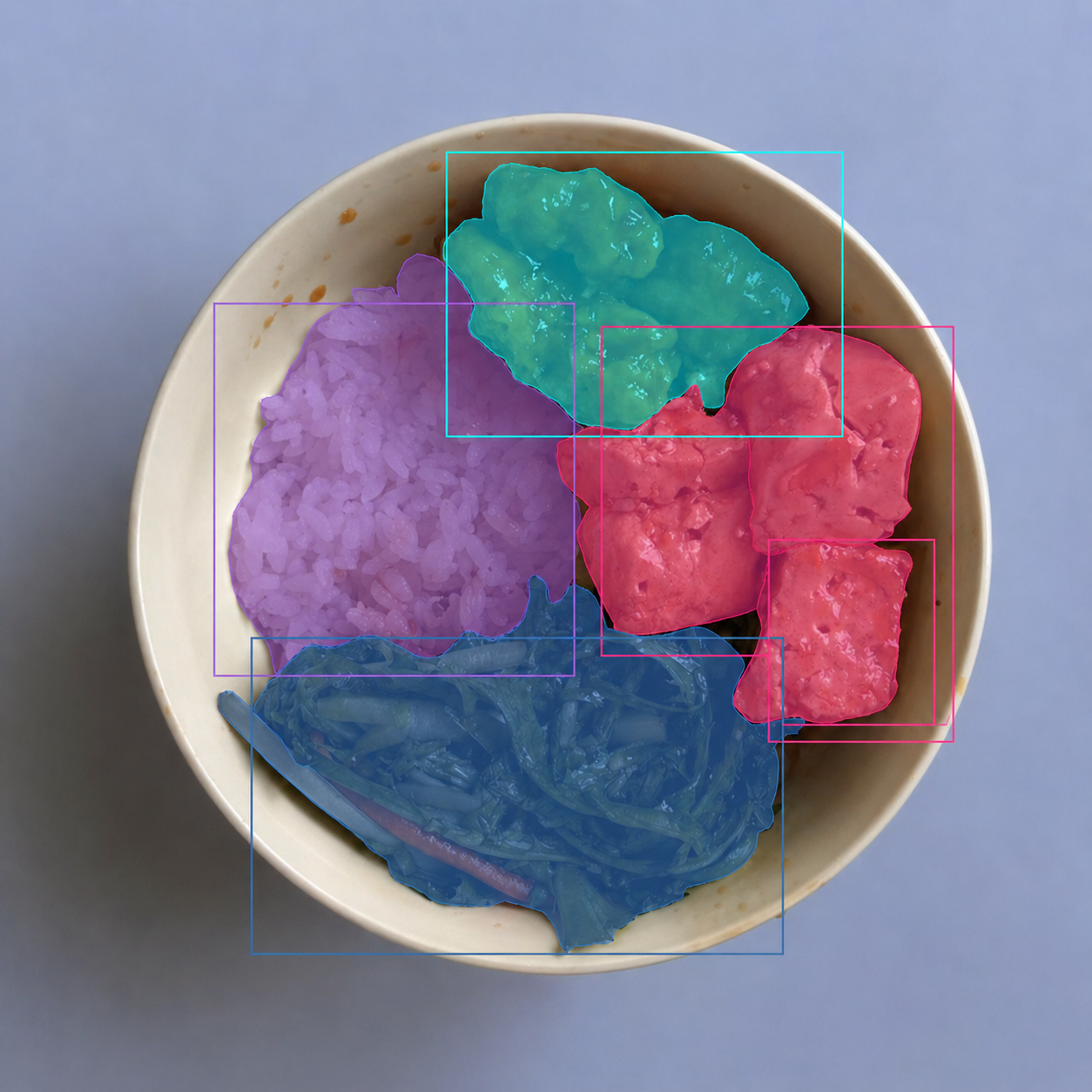

Supplement: Supplementary file 1 [file nutrients-18-02119-s001.zip › S1.Semantic segmentation graphs of different models/No.4-6.png]

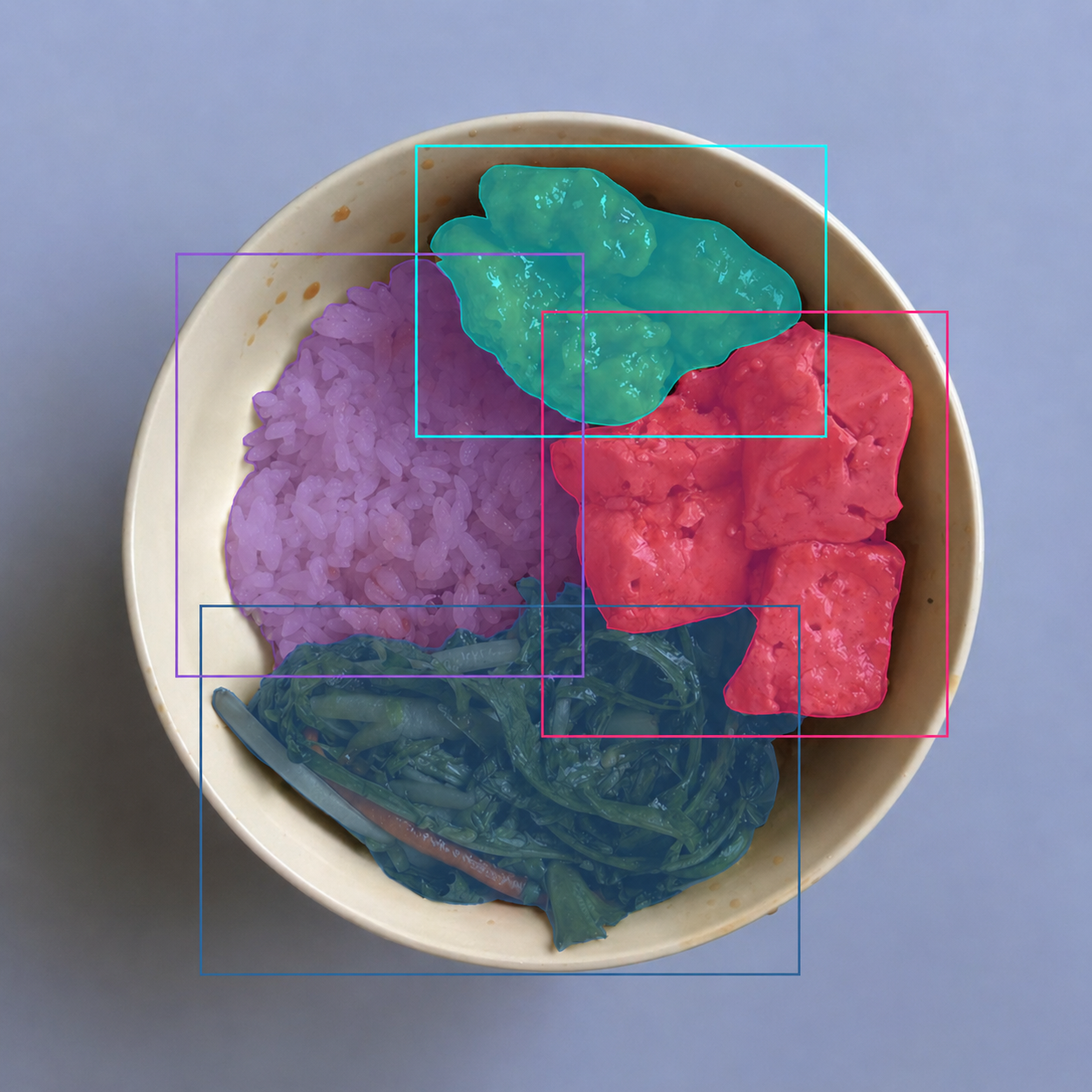

Supplement: Supplementary file 1 [file nutrients-18-02119-s001.zip › S1.Semantic segmentation graphs of different models/No.4-7.png]

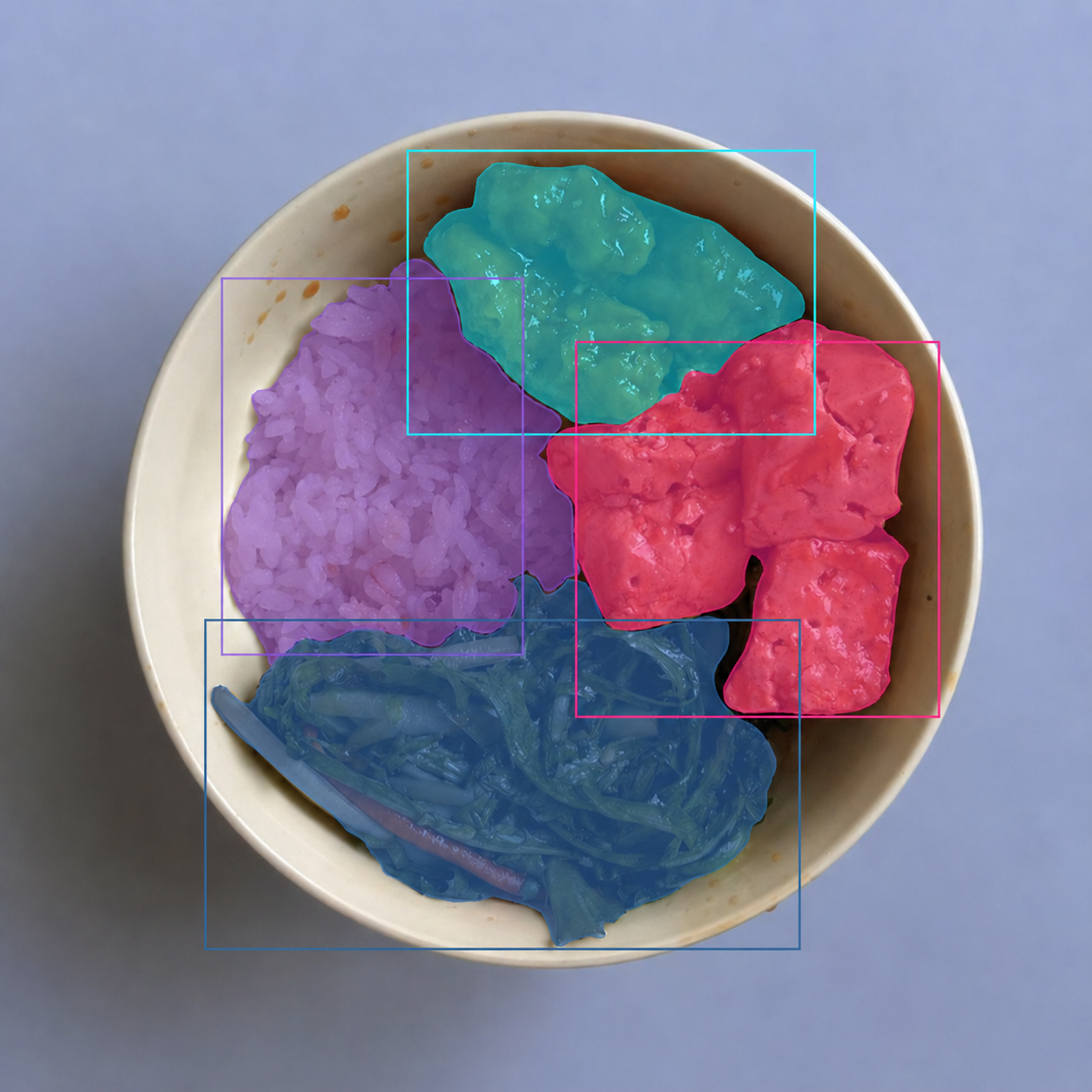

Supplement: Supplementary file 1 [file nutrients-18-02119-s001.zip › S1.Semantic segmentation graphs of different models/No.4-8.png]

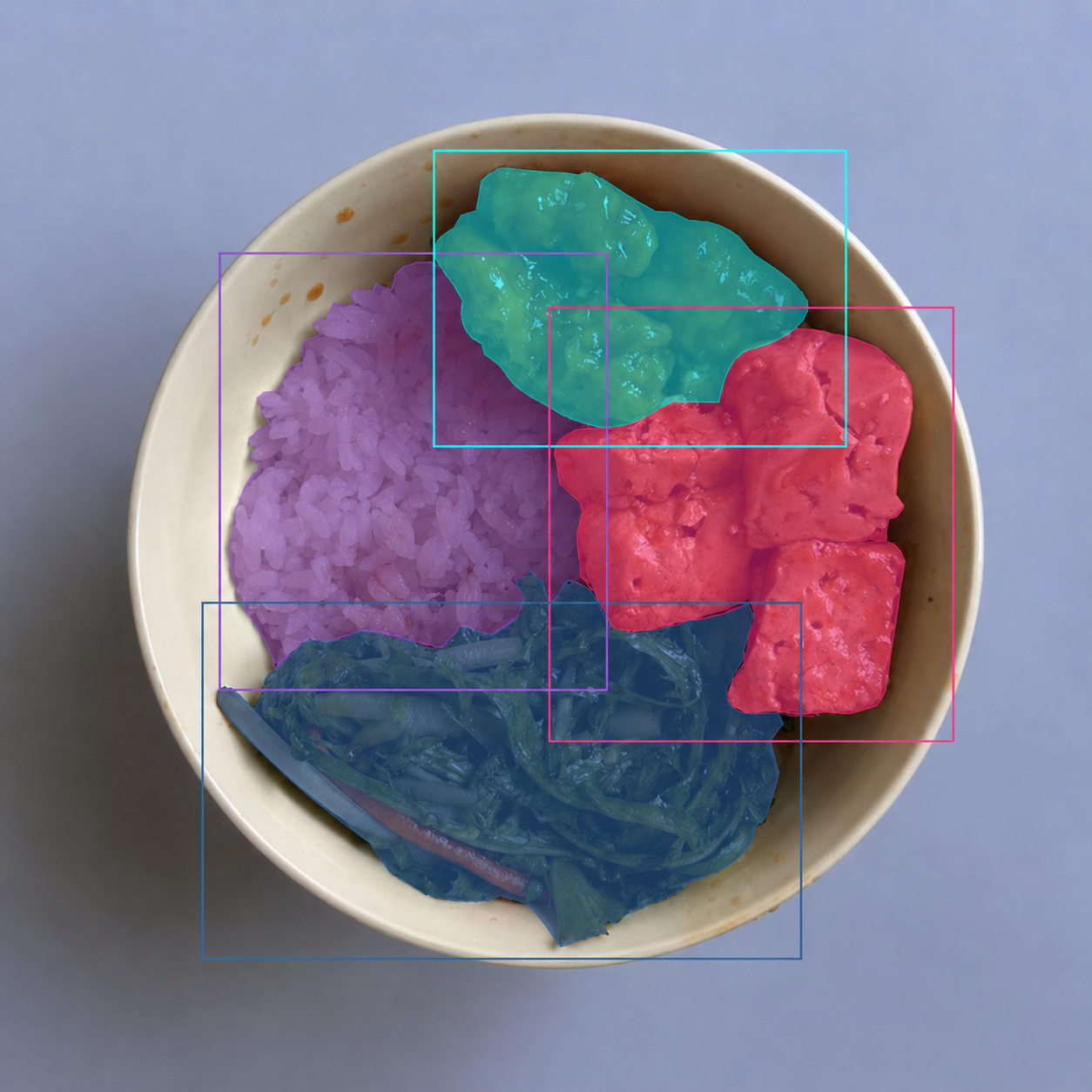

Supplement: Supplementary file 1 [file nutrients-18-02119-s001.zip › S1.Semantic segmentation graphs of different models/No.4-9.png]

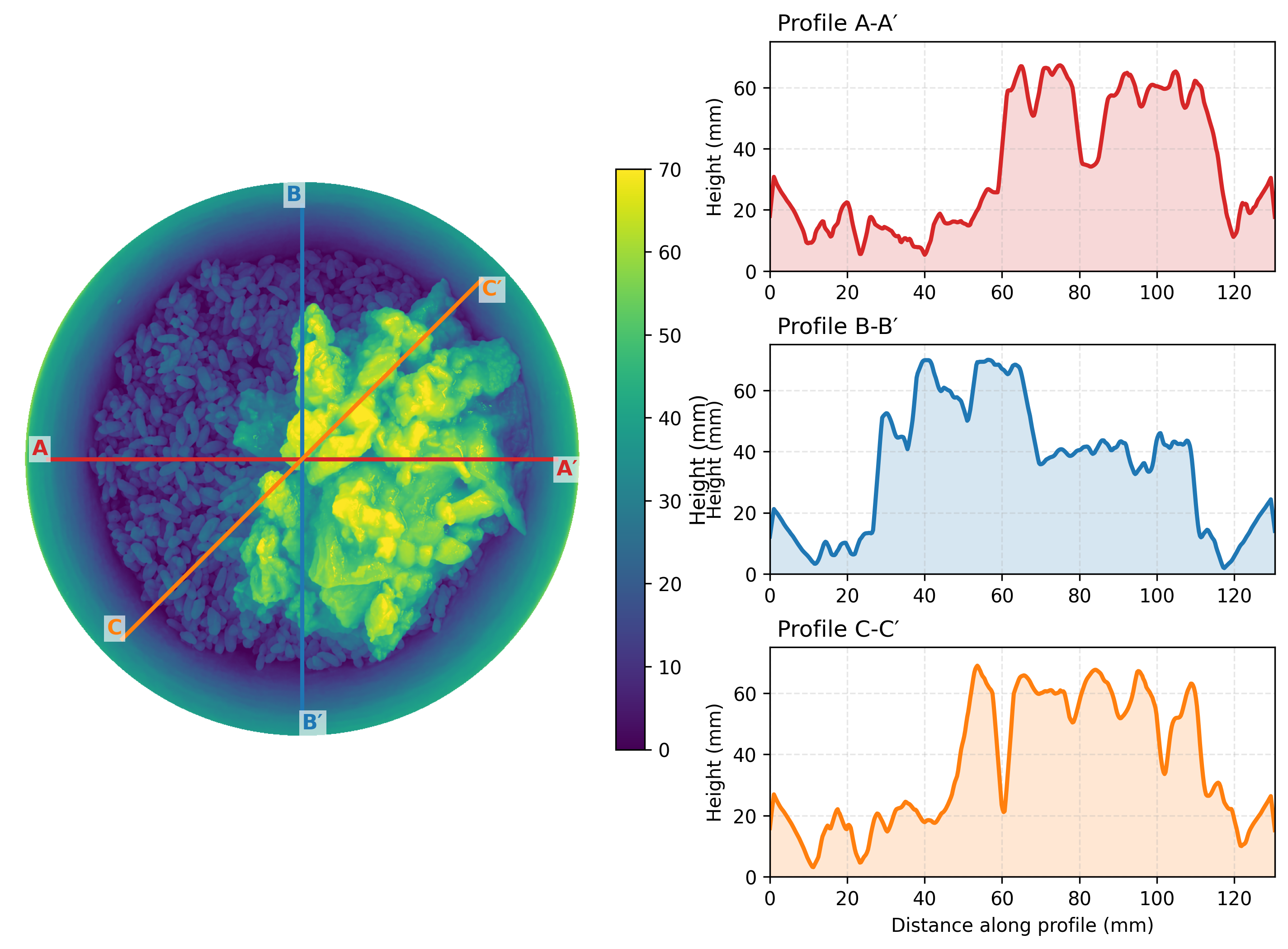

Supplement: Supplementary file 1 [file nutrients-18-02119-s001.zip › S2.Height field pseudo-color images/height_profile_combo1.png]

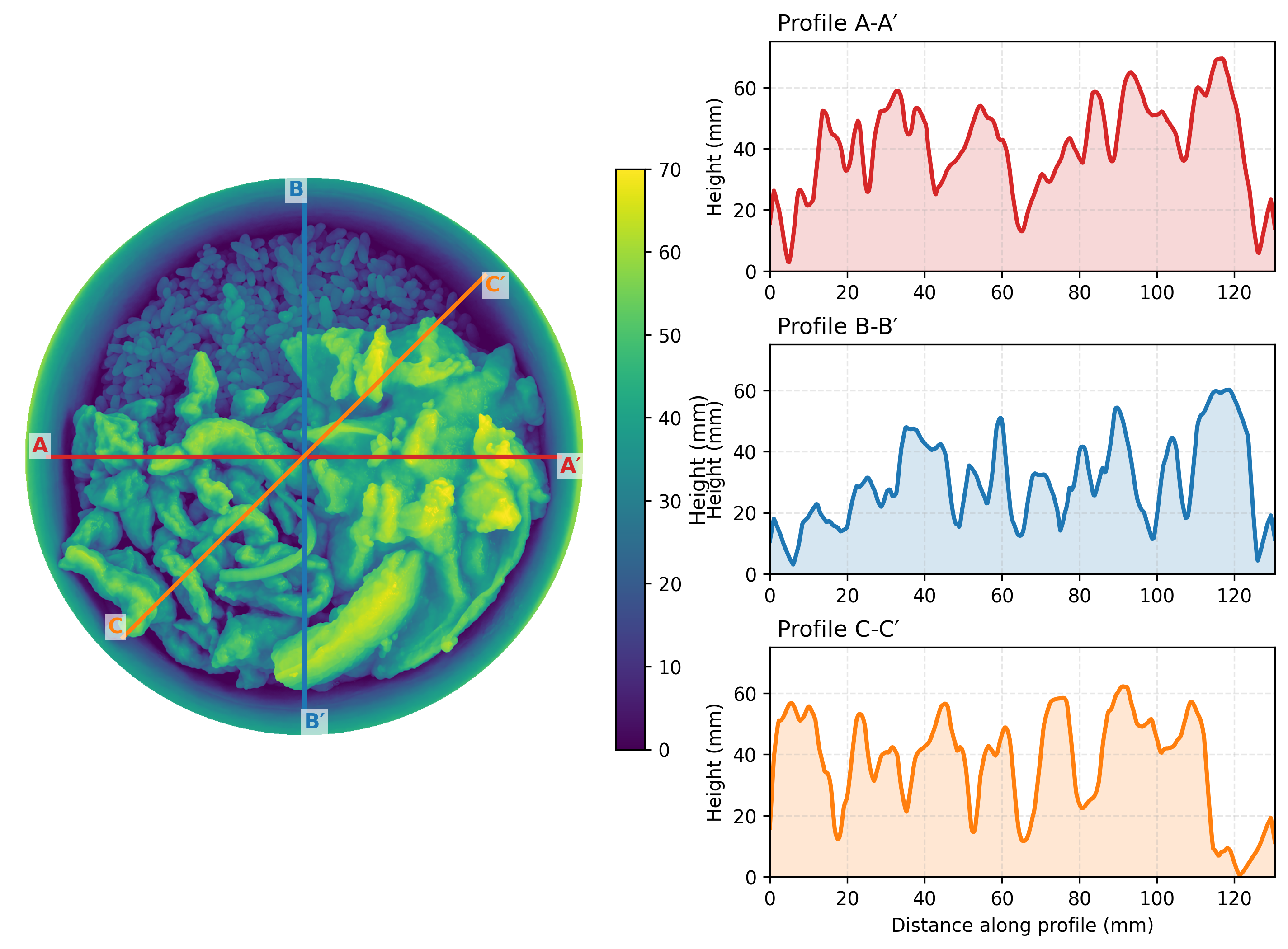

Supplement: Supplementary file 1 [file nutrients-18-02119-s001.zip › S2.Height field pseudo-color images/height_profile_combo2.png]

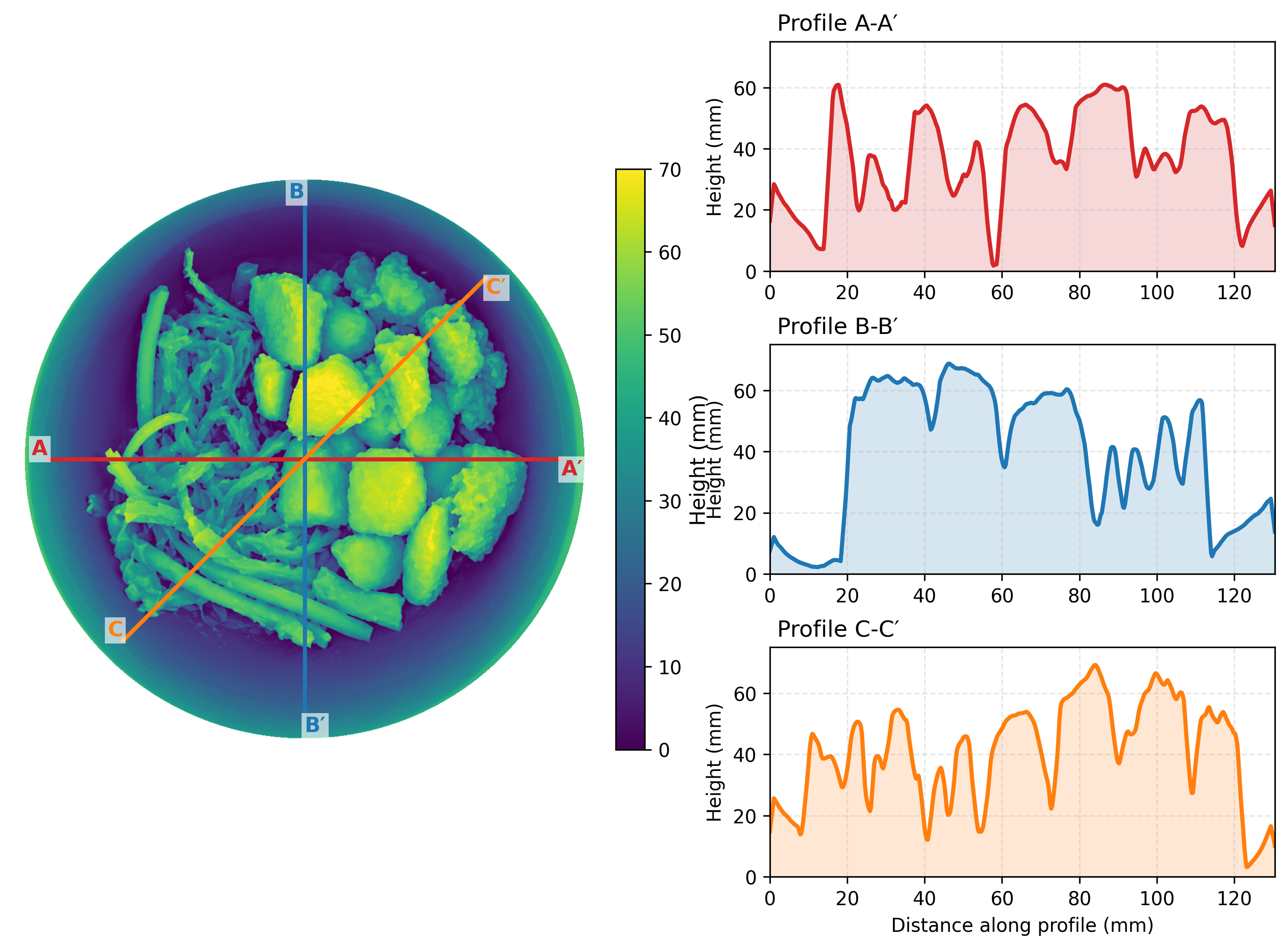

Supplement: Supplementary file 1 [file nutrients-18-02119-s001.zip › S2.Height field pseudo-color images/height_profile_combo3.png]

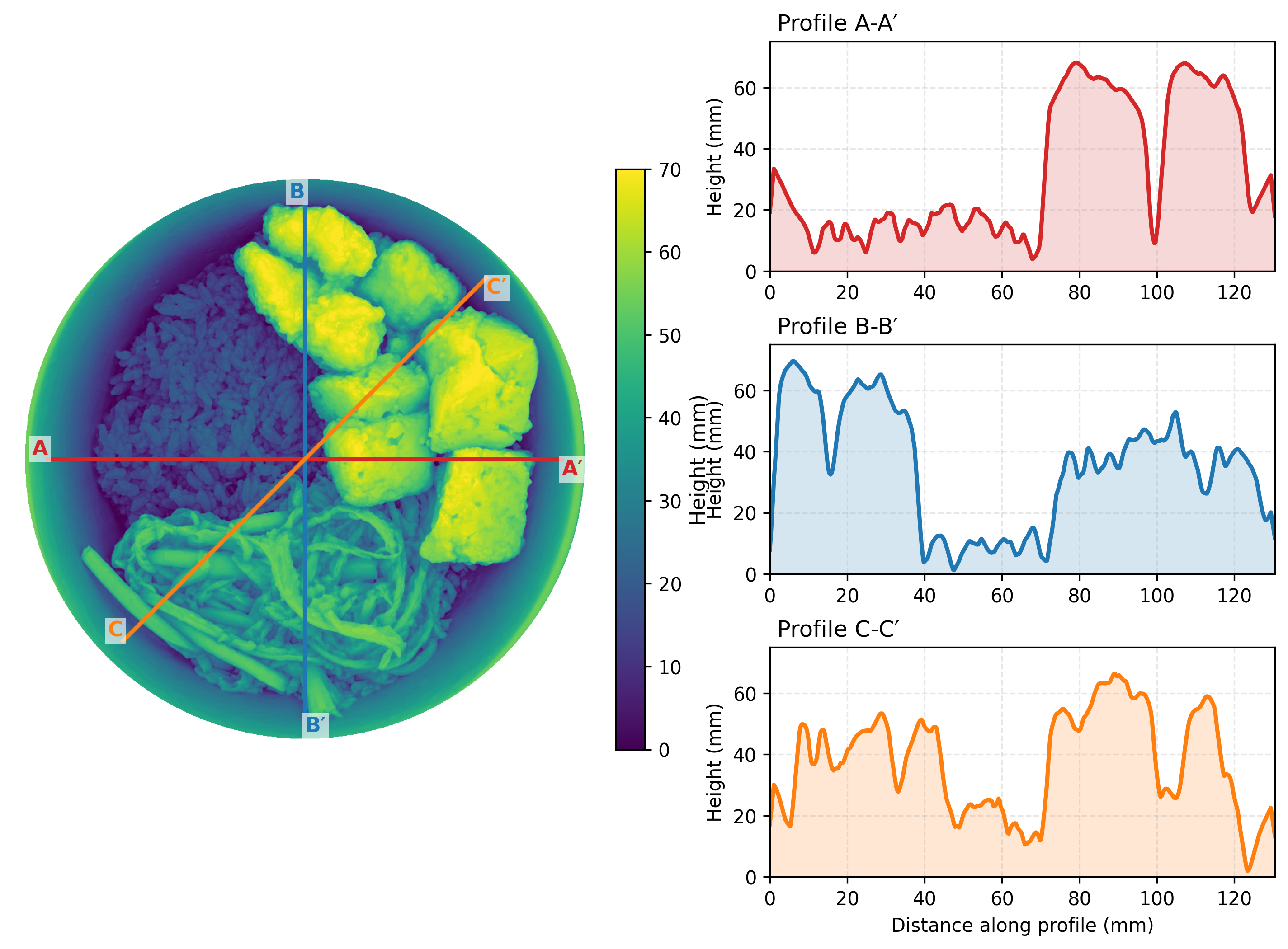

Supplement: Supplementary file 1 [file nutrients-18-02119-s001.zip › S2.Height field pseudo-color images/height_profile_combo4.png]

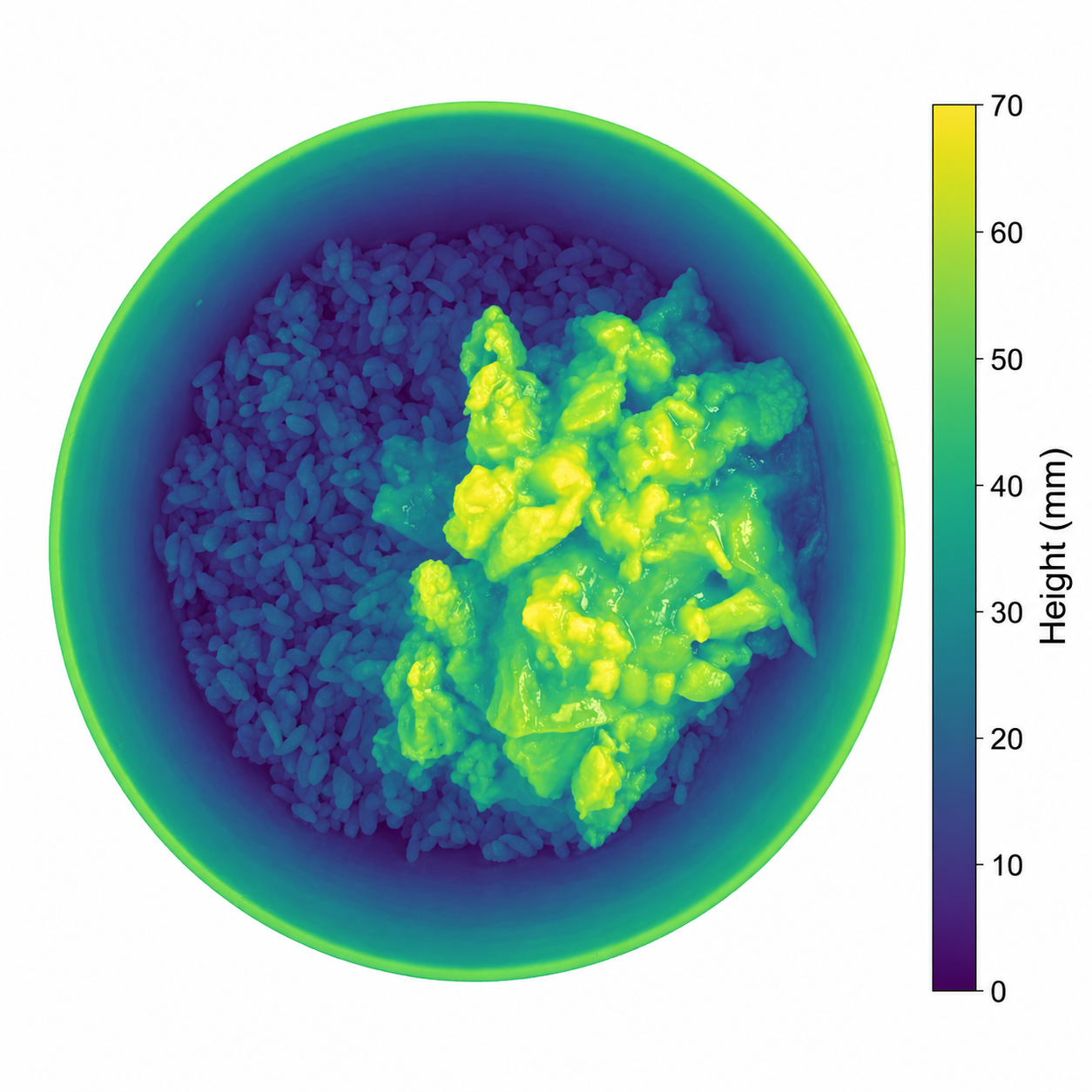

Supplement: Supplementary file 1 [file nutrients-18-02119-s001.zip › S2.Height field pseudo-color images/NO.1G.png]

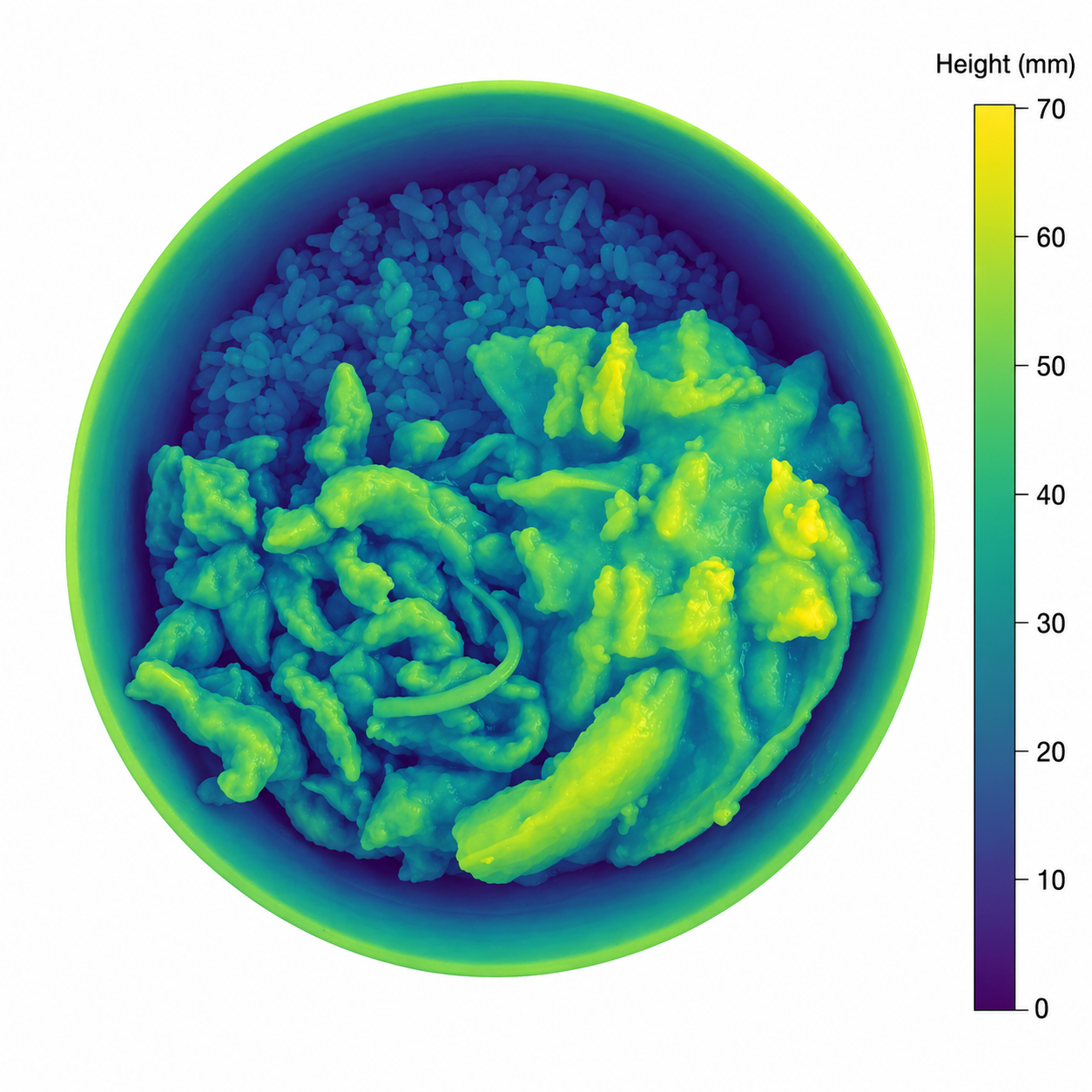

Supplement: Supplementary file 1 [file nutrients-18-02119-s001.zip › S2.Height field pseudo-color images/NO.2G.png]

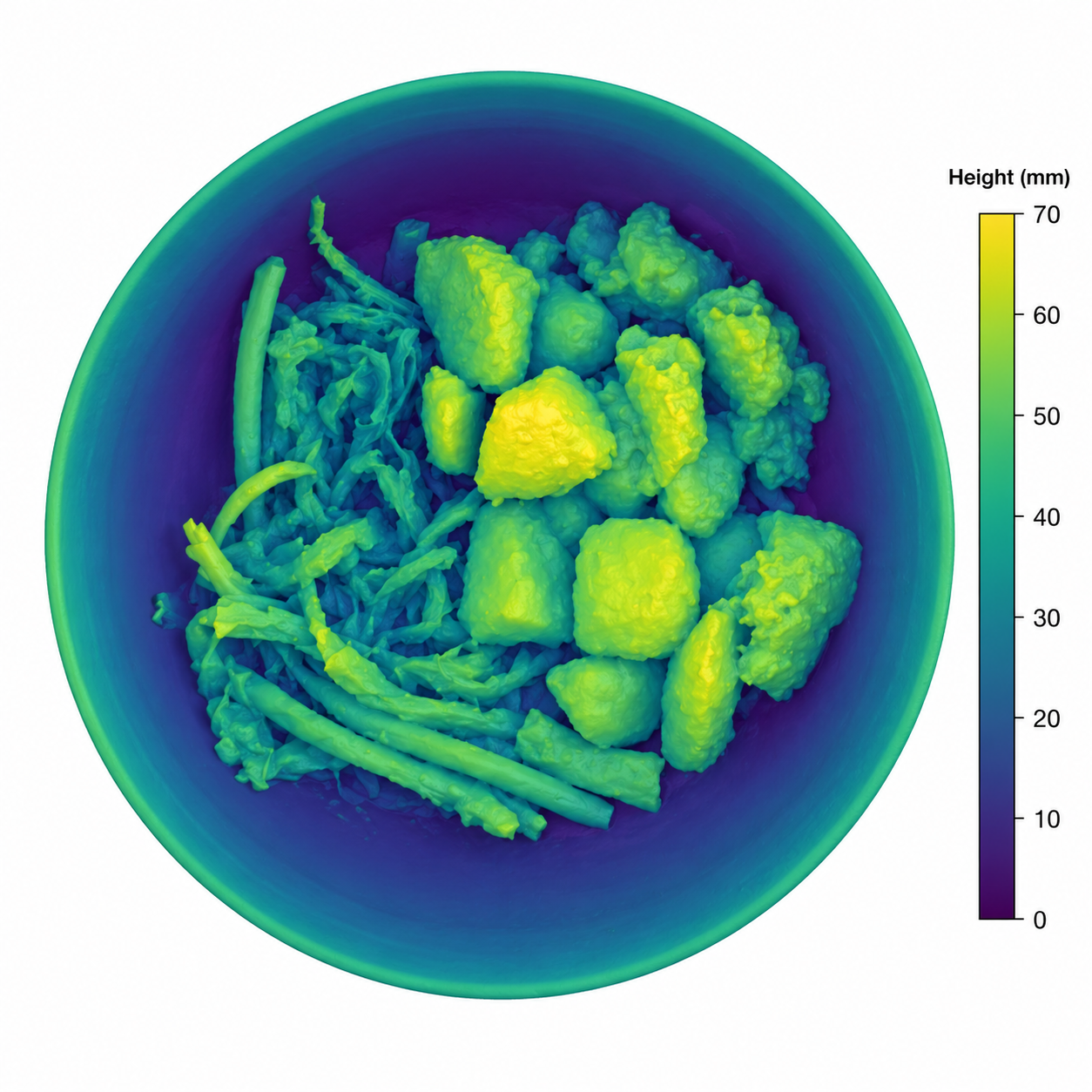

Supplement: Supplementary file 1 [file nutrients-18-02119-s001.zip › S2.Height field pseudo-color images/NO.3G.png]

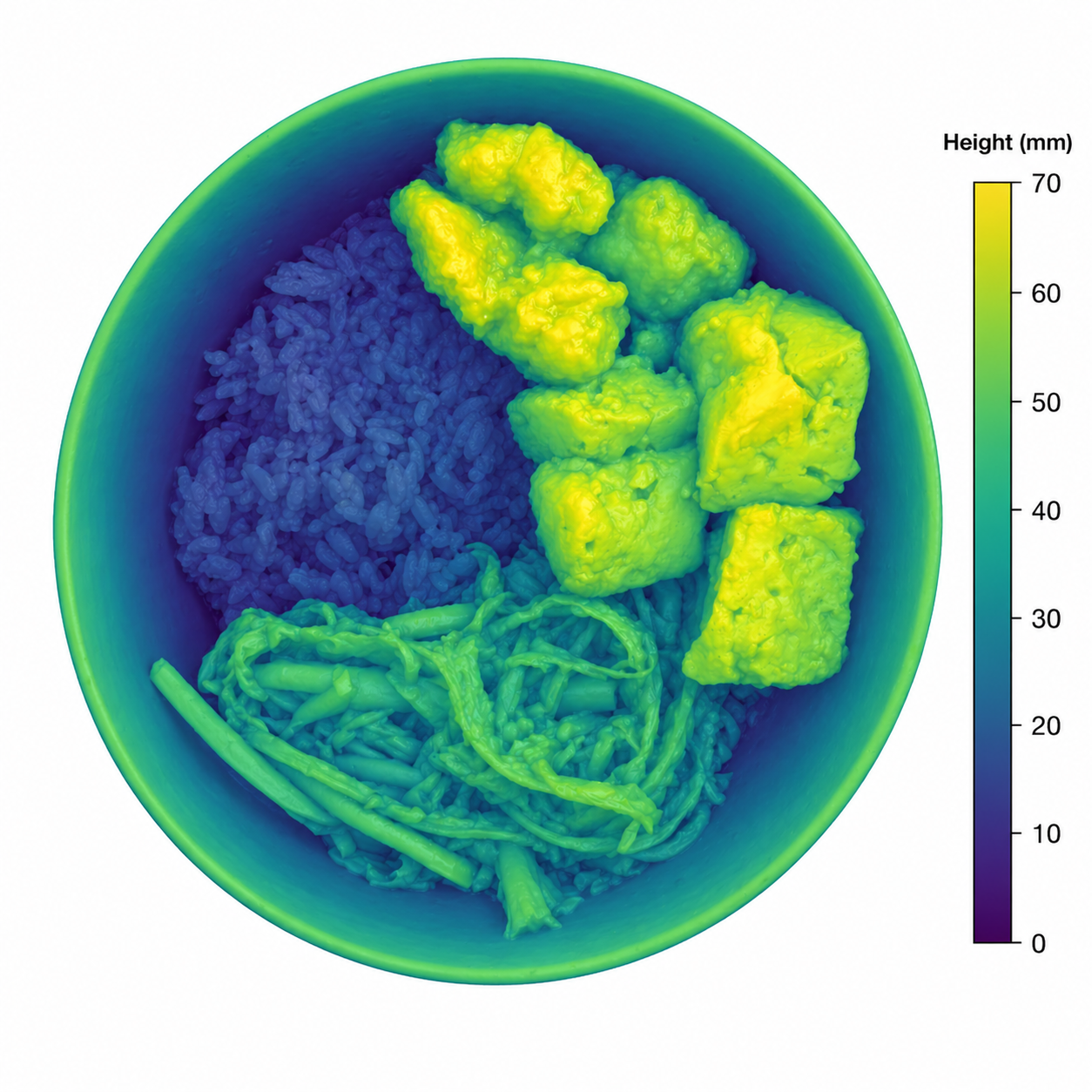

Supplement: Supplementary file 1 [file nutrients-18-02119-s001.zip › S2.Height field pseudo-color images/NO.4G.png]

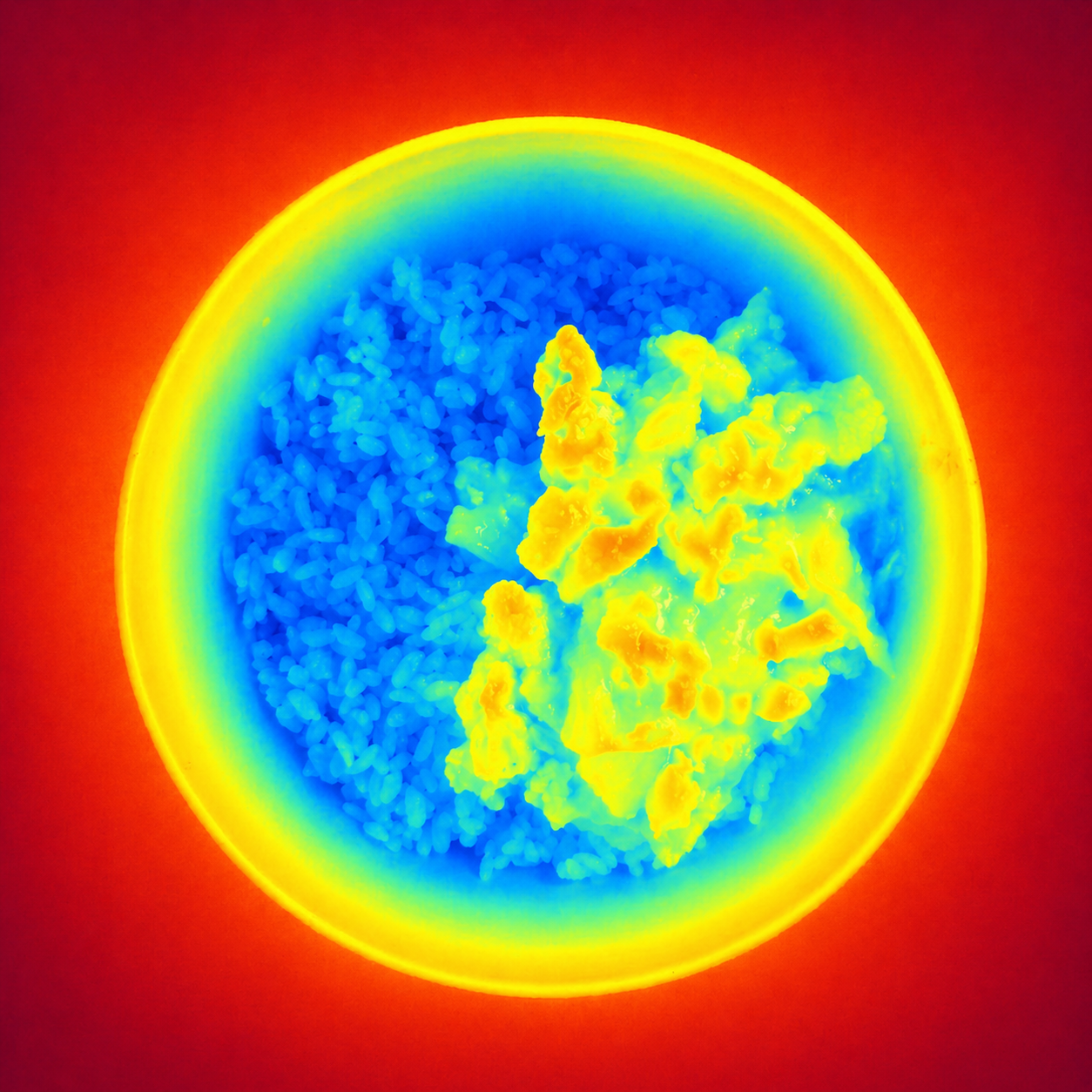

Supplement: Supplementary file 1 [file nutrients-18-02119-s001.zip › S3.RGB-D images/No.1.png]

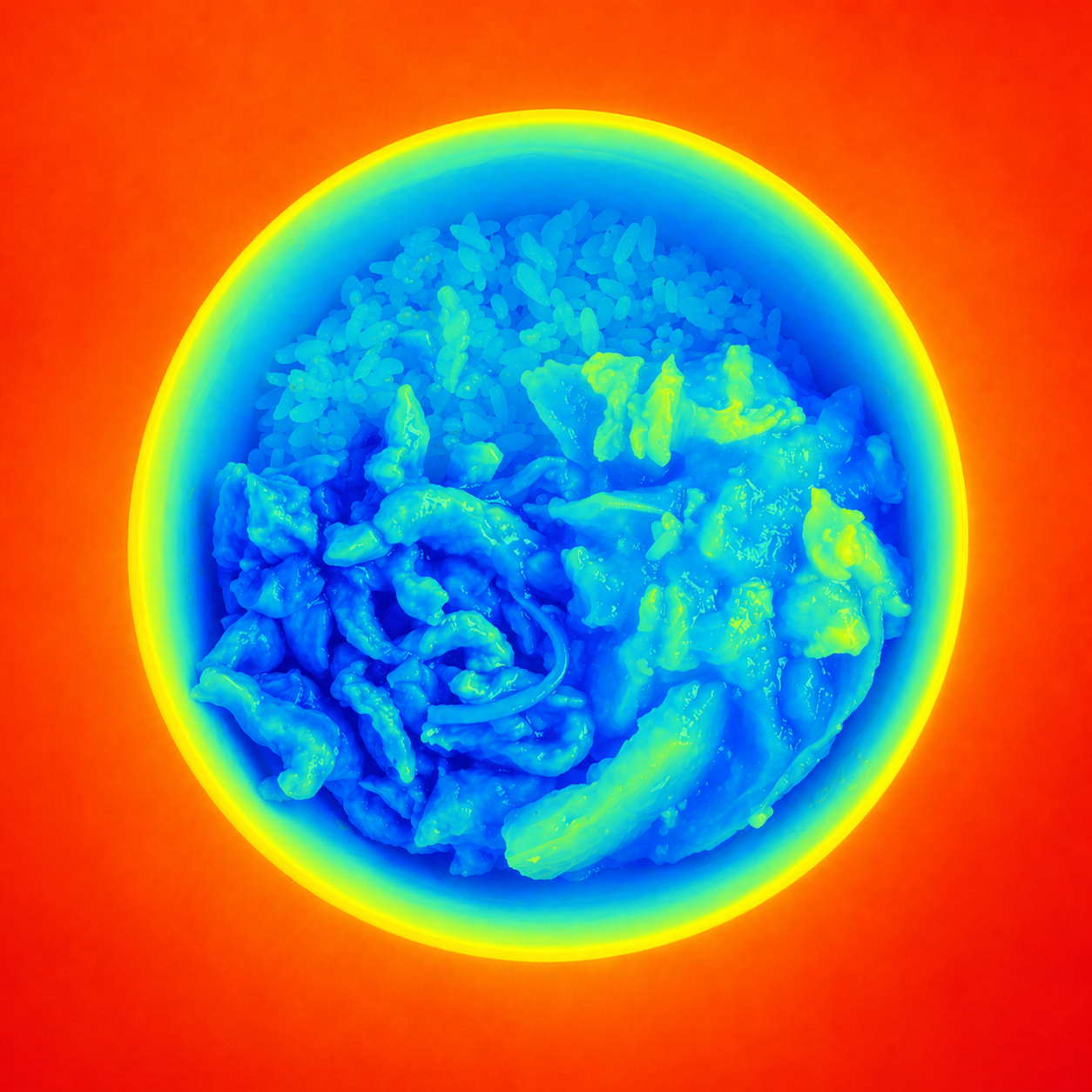

Supplement: Supplementary file 1 [file nutrients-18-02119-s001.zip › S3.RGB-D images/NO.2.png]

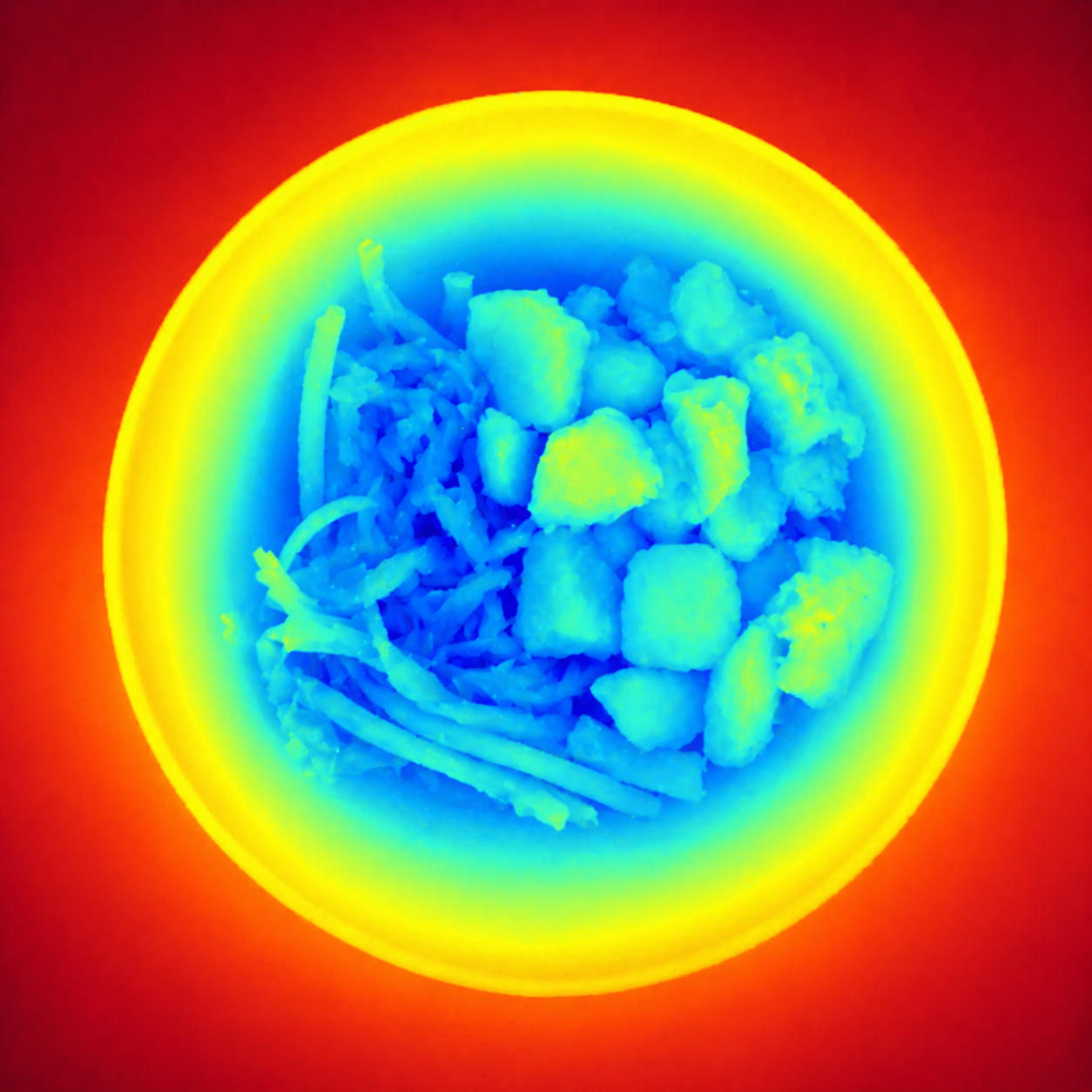

Supplement: Supplementary file 1 [file nutrients-18-02119-s001.zip › S3.RGB-D images/NO.3.png]

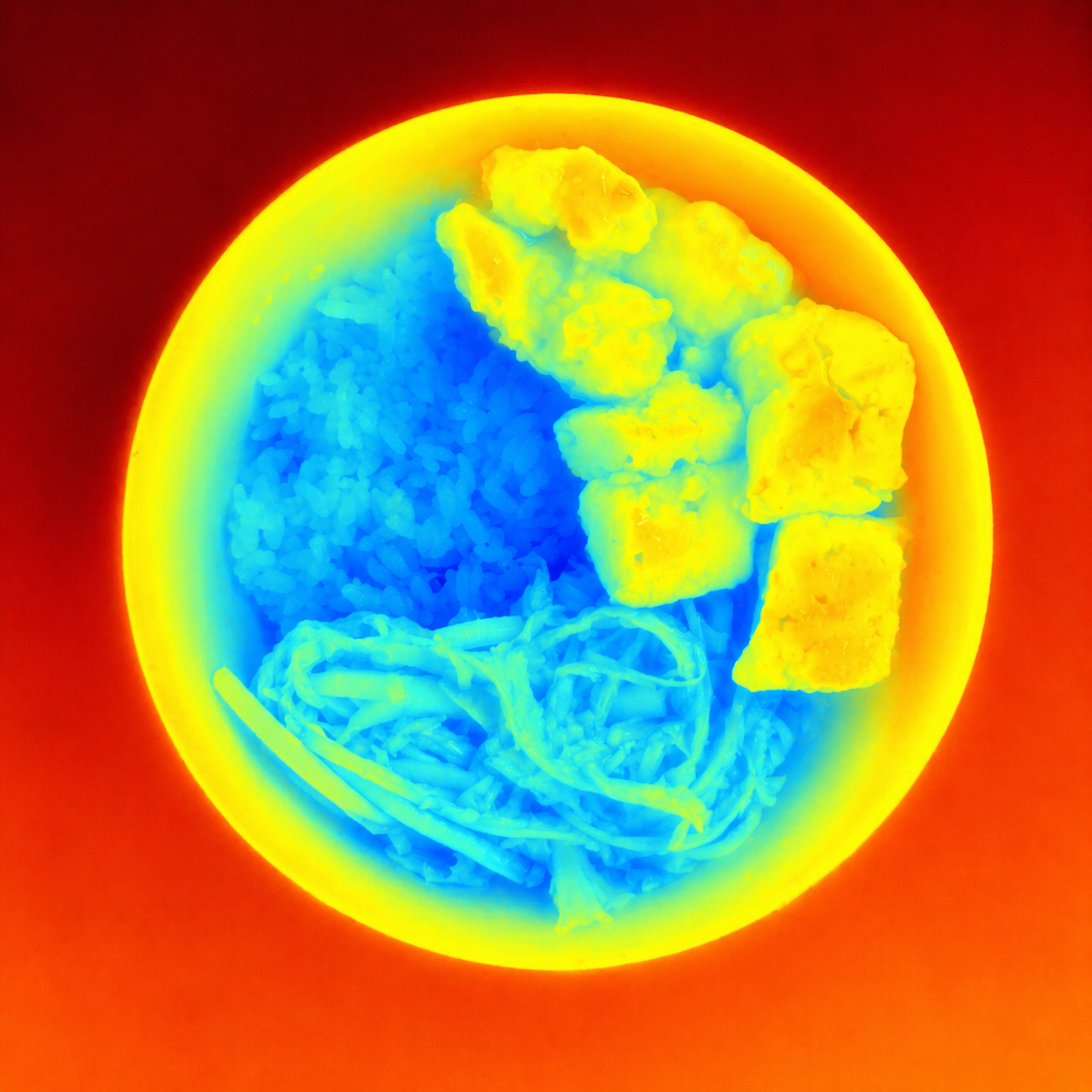

Supplement: Supplementary file 1 [file nutrients-18-02119-s001.zip › S3.RGB-D images/NO.4.png]
